# A Focus on Natural Autophagy Modulators as Potential Host‐Directed Weapons Against Emerging and Re‐Emerging Viruses

**DOI:** 10.1002/med.70003

**Published:** 2025-07-16

**Authors:** Ilaria Cursaro, Sara Rossi, Stefania Butini, Sandra Gemma, Gabriele Carullo, Giuseppe Campiani

**Affiliations:** ^1^ Department of Biotechnology, Chemistry and Pharmacy University of Siena Siena Italy

**Keywords:** antivirals, autophagy, host‐targeting antivirals, natural products

## Abstract

Autophagy is a highly conserved intracellular process involved in maintaining homeostasis and in the degradation of damaged organelles and external pathogens. Nature provides complex and varied reservoirs of scaffolds and chemical entities that may have a pivotal role in the search for new therapeutic leads. Among them, phytocompounds have been amply exploited in the search for new autophagy modulators to tackle diseases like cancer and degenerative disorders, while their use as potential antiviral agents has been explored to a limited extent. The modulation of autophagy in viral infections may play a dual and contraposing role. Depending on the replication mechanism of the virus, it may serve as an adjuvant in the innate immune response of the host, or it may be hijacked by viruses, favoring their replication. This review is intended to present an overview of antiviral natural compounds and extracts capable of modulating autophagy, and it seeks to provide a solid foundation for researchers to further investigate the mechanisms of autophagy modulation during viral infections and to identify diverse molecular entities for antiviral drug discovery.

## 1 Introduction

Autophagy is a highly regulated cellular process that involves the degradation and recycling of damaged or unnecessary cellular components. The term derives from Greek, meaning “self‐eating,” and describes the cell's ability to sequester and degrade portions of its cytoplasm within autophagosomes, which are then fused with lysosomes to break down and recycle the engulfed contents [1]. Autophagy is closely related to apoptosis, another key process in cellular homeostasis, but while autophagy functions as a survival mechanism by recycling cellular components under various conditions, apoptosis is a programmed cell death pathway that removes damaged or unnecessary cells and components [2]. These two mechanisms are distinct but interconnected, and both are essential for maintaining the homeostatic balance, especially in the context of viral infections and infectious diseases [3].

Targeting autophagy presents a promising antiviral strategy thanks to its role in viral replication, pathogenicity, and infectivity. In fact, viruses often manipulate autophagy by inducing or inhibiting the initiation process and the machinery functional complexes to create a favorable environment for their replication and to evade the host immune responses; hence, the pharmacological regulation of autophagy can lead to the disruption of these viral processes, limiting infection and disease progression [4, 5].

Natural compounds have gained significant attention as modulators of autophagy due to their broad and diverse biological activities and relatively low toxicity [6]. These regulators, derived from plants, fungi, marine algae, and organisms, offer a potential avenue for the design of new antiviral agents by targeting the host's autophagy machinery [7].

This review provides an overview of the interplay between autophagy and viral infections, with an emphasis on plant‐derived molecules with potential antiviral activity by targeting the host autophagy machinery through induction, exploitation, inhibition, or evasion. This study reviews known natural autophagy modulators presenting an antiviral activity supported by either biological or in silico studies, focusing on the mechanisms involved in the manipulation of the host degradative pathway. Data were collected through a comprehensive analysis of the literature, which was performed using electronic databases such as PubMed, Scopus, and Google Scholar. The search strategy included keywords related to the object topic, including peer‐reviewed articles, reviews, and in silico studies that explore the relationship between autophagy and viral infections. Published articles were considered for inclusion, with no restrictions on publication date. The selection of natural compounds was based on their documented effects on autophagy and antiviral activity, ensuring a focus on both well‐established and emerging research findings, highlighting (1) the investigation of the autophagy modulation properties of isolated natural compounds with an antiviral aim, (2) the exploitation of isolated natural compounds as benchmark autophagy modulators in antiviral experimental settings, and (3) relevant insights into the autophagy modulation capabilities of isolated natural compounds in non‐antiviral research, in which authors identified their potential for either future repurposing of natural compounds or the development of new semisynthetic derivatives hampering viral replication in host‐targeting antiviral endeavors. The ultimate aim is to revise current knowledge of natural autophagy modulators in viral infections, identify potential gaps, and outline perspectives for future research.

### 1.1 Role of Autophagy

Autophagy and the ubiquitin (Ub)–proteasome system (UPS) are distinct, parallel, and conserved mechanisms that play a crucial role in balancing synthesis and degradation of cellular contents and in host immune responses, in different physiological, stressed, or pathogenic conditions [2, 8].

The degradation path of potentially harmful or reusable cellular waste materials, as damaged proteins or cytosolic organelles, involves the activation of the autophagic machinery that helps guarantee cellular homeostasis, while also preventing the threat of external stimuli or pathogens. Autophagy is correlated with important host–virus interactions. During microbial infections, the host autophagic machinery is involved in viral infection responses by degrading viral components, by regulating the intensity of the inflammatory response, or by promoting viral antigen presentation through histocompatibility complexes. However, autophagy plays a controversial role in viral infections, as it also provides lipid membranes and vectors for viral maturation and exit from the host cells, increasing viral load [9, 10].

This dual and contrasting role of autophagy can be clarified by classifying viruses into two categories. The first lists viruses that evade or inhibit autophagy, as this degradation pathway is activated to combat viral infections by engulfing cytoplasmic virions or viral particles and delivering them to lysosomes for degradation. A notable example is the herpes simplex virus (HSV) that negatively regulates autophagy. The second includes viruses that induce and hijack this machinery to evade the host immune system or exploit the machinery for viral replication [11]. Hepatitis C virus (HCV) is known to induce this process through multiple signaling pathways. Additionally, autophagy also promotes the immune signaling cascade for the inflammatory response and facilitates the antigen presentation, so viruses have evolved to inhibit or evade this mechanism.

### 1.2 Autophagy: Classification and Functions

Since its first identification in the '60s by Duve et al. [12], progress in the understanding of the autophagic machinery has led to the identification of the genes involved in this process in yeast species, allowing the recognition of the autophagy‐related genes (ATGs) and proteins. Further studies have led to a breakthrough in this important mechanism, whose deregulation and consequent modulation have been exploited as a therapeutic strategy in many pathologies, such as cancer [13, 14, 15], cardiomyopathies, viral infections, adipose tissue, and skeletal muscle issues, but also neuro‐ and retinal degeneration [16, 17].

**Figure 1:**
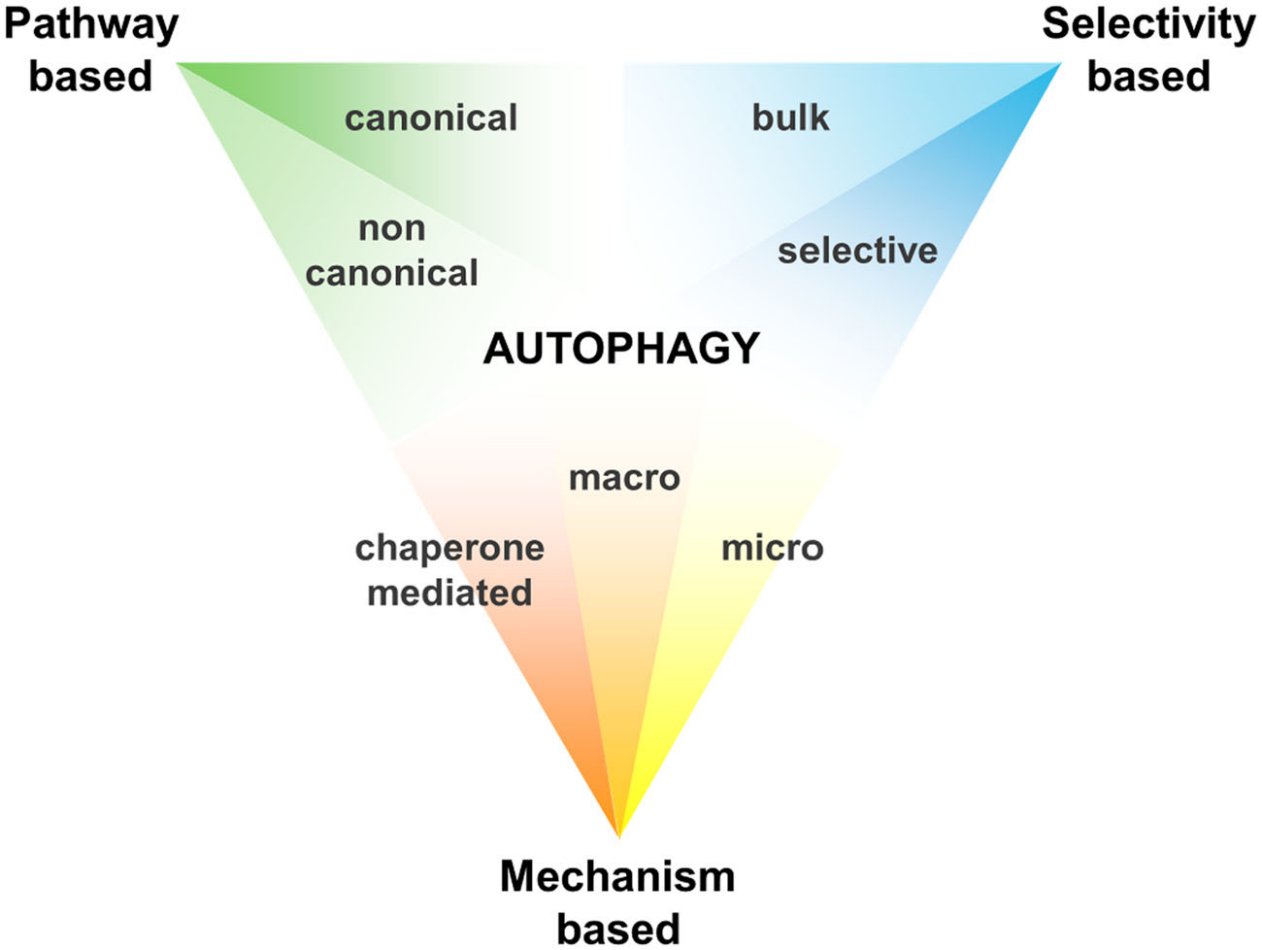
Autophagy can be classified based on the pathway, the mechanism, or the selectivity. These mechanisms are not mutually exclusive but are interrelated, with a significant overlap between categories, reflecting the complex and multifaceted nature of the regulation of this machinery. [Color figure can be viewed at wileyonlinelibrary.com]

Autophagy can be classified into categories (Figure 1) based on

- the specific delivery mechanism involved (chaperone‐mediated, micro‐, and macro‐autophagy) [18, 19]:
- the pathway involved (canonical and noncanonical);
- the selectivity of the process (bulk and selective autophagy, which will be further described).

Chaperone‐mediated autophagy occurs through the translocation of cytoplasm components with transporters, without the formation of the double‐membrane vesicles. Micro‐autophagy, further classified in selective or nonselective autophagy depending on the entry mechanism inside the lysosomes, directly takes place via invaginations of the lysosomal membrane and subsequent degradation inside the lysosome. Macro‐autophagy is a highly conserved intracellular mechanism in both plants and animals; it is mediated by the formation of a double‐layered membrane vesicle within the lysosomes or vacuoles, defined as an autophagosome. The autophagosome transports the “cargo” to the lysosome, where it fuses to form an autolysosome, enabling the degradation of cytosolic material. In this review, the main focus will be given to macro‐autophagy, which will simply be noted as autophagy from now on [9]. Selective macro‐autophagy can, in turn, be categorized as xenophagy, mitophagy, pexophagy, aggrephagy, reticulophagy (ER‐phagy), and lipophagy depending on the specific cargo and receptors involved [20].

### 1.3 The Autophagic Machinery

**Figure 2:**
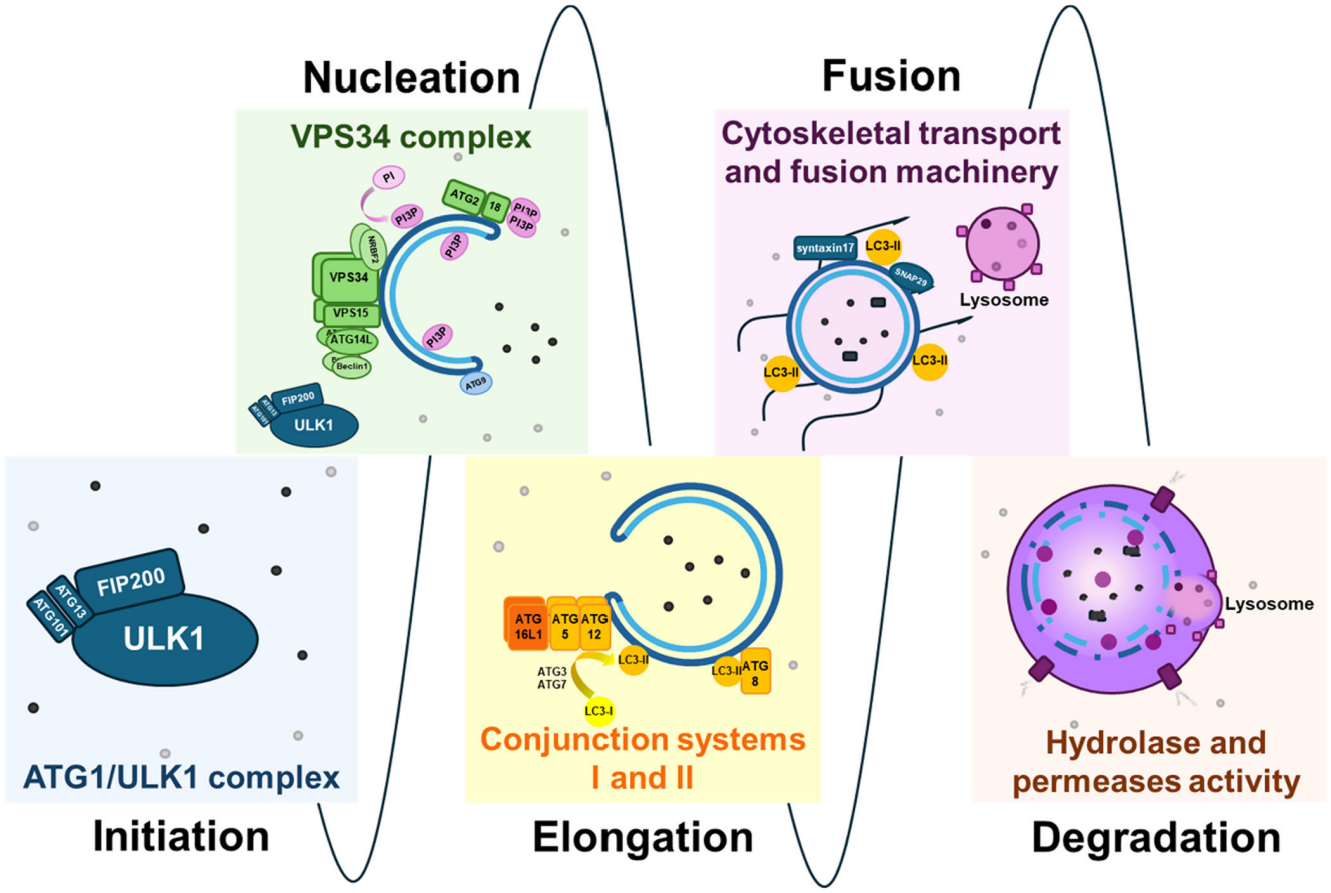
The autophagic mechanism can be divided into five finely regulated and subsequent steps: Initiation, Nucleation, Elongation and Maturation, and Fusion and Degradation, each of which is maneuvered by a specific functional complex. [Color figure can be viewed at wileyonlinelibrary.com]

The main step in the autophagic process is the formation of the autophagosome. This process is regulated by more than 30 ATG proteins that form complexes in a sequential order and allow for the autophagy initiation, nucleation, phagosome expansion, cargo uptake, autophagosome closure, fusion, and content degradation [8, 21]. Some of these ATG proteins are related to the UPS, playing crucial roles in the degradation pathway, yet with different roles [22]. The ATG proteins can be identified into six functional complexes which operate in distinct steps (Figure 2) of the autophagic process:

– ATG1/ULK1 complex (ATG1/unc‐51 like autophagy activating kinase 1 complex), formed by ATG1‐13‐17‐29‐31
– VSP34 (vacuolar protein‐sorting 34) complex or ATG6/Beclin1‐phosphatidylinositol 3‐Kinase complex I (known as PI3K‐C1), formed by Beclin1‐ATG14‐15‐34
– ATG9 membrane protein
– ATG2‐18/PI3P‐binding complex (ATG2‐18/phosphatidylinositol‐3‐phosphate‐binding complex)
– ATG12/ATG5/ATG16L1 ubiquitination‐like conjunction system I
– ATG8/LC3 ubiquitination‐like conjunction system II (ATG8/1 A/1B‐light chain 3)

#### 1.3.1 Initiation

An initiating signal is required to activate the autophagic machinery. This signal can be triggered by different stimuli, leading to bulk or selective autophagy initiation. The former is activated in response to cellular stress, a lack of nutrients, or energy requirements, and is regulated through AMPK (AMP‐activated protein kinase) and mammalian target of rapamycin complex 1 (mTORC1) pathways. The latter can be activated even in the abundance of nutrients, by intracellular pathogens, microbial induction, damaged organelles, or oxidative stress, and is mediated by specific receptors which can be involved in both Ub‐dependent and ‐independent autophagy [23]. Additionally, selective autophagy has been classified according to the conveyed cargo (i.e., aggrephagy, ER‐phagy, lipophagy, mitophagy, nucleophagy, lysophagy, pexophagy, ferritinophagy, and xenophagy). Nonetheless, these different induction signals converge into a common pathway [24] in which the initiation of autophagy is operated through the ATG1/ULK1 complex [23], which is formed by a serine/threonine protein kinase, ATG13, FAK family‐interacting protein of 200 kDa (FIP200), and ATG101. ATG1 was first described in *Saccharomyces cerevisiae* (as APG1p) and is a highly conserved complex among eukaryotes. Orthologs of this complex have also been identified in other species such as *Schizosaccharomyces pombe* and *Caenorhabditis elegans*, clearly demonstrating their role in autophagy initiation. The mammalian homologs which have been identified are ULK1–2, and three other related proteins, ULK3, ULK4, and serine/threonine‐protein kinase‐36 (STK36). Of these five homologues, only ULK1 and 2 are thought to participate in autophagy signaling. The remaining three homologues indeed show similarity in the N‐terminal domain, but present important differences in the C‐terminal domain, which is crucial for binding with FIP200 and ATG13 during the initiation process [25], allowing for the formation of the induction complex.

This first initiation complex is modulated by various signals and complexes depending on the activation of bulk or selective autophagy; among these, mTORC1 is one of the principal regulators of autophagy, responsible for the management of metabolism and cell growth [8]. mTOR is a serine/threonine kinase that is activated by the presence of cytosolic amino acids, growth factor signaling, and energy deprivation. It inhibits autophagy by suppressing the catalytic activity of ULK1 and ATG13 through their phosphorylation [26]. AMPK can participate in autophagy induction by inactivating mTORC1 or by activating ULK1 phosphorylation. Additionally, during selective autophagy cargo receptors come in play regulating the ULK1 recruitment of the autophagic machinery [23], an example are the nuclear dot protein 52 (NDP‐52) and optineurin (OPTN) proteins which are involved in mitophagy initiation. At the same time, ULK1 can per se induce autophagy through phosphorylation of the autophagy and Beclin1 regulator 1 (AMBRA1) and the cargo adaptor protein p62, increasing the binding affinity of p62 for Ub or through phosphorylation of FUN14 Domain Containing 1 (FUNDC1), thereby promoting mitophagy [24, 26].

#### 1.3.2 Nucleation

The recruitment of the ATG proteins in proximity to various organelles throughout the cytoplasm allows for the formation of a double‐membraned organelle called the “omegasome,” that serves as a scaffold, guiding the initial shaping of the phagophore and ensuring proper curvature and expansion of the membrane [21]. During this process, two kinase complexes are involved, the VPS34 complex I and the PI3P‐kinase complex II, which play crucial, yet different, roles during elongation and maturation of the phagophore, respectively [24, 27, 28, 29]. In fact, upon autophagy induction, the ULK1/ATG1 complex moves toward the autophagy initiation sites and controls the enrollment of the VPS34 complex. The recruitment of VPS34 and its translocation are driven by the phosphorylation of Ser15 and other sites of Beclin1 and by the ATG14L subunit [24]. This complex accounts for the release of PI3P and other proteins that manage the formation and expansion of the phagophore, while also recruiting a third important complex, the ubiquitination‐like conjugation system [24, 26]. Specifically, after recruitment, the VPS34 complex interacts with ATG14L and Beclin1 to bind an additional subunit: the nuclear receptor binding factor 2 (NRBF2). This subunit enhances kinase activity and dimerization, although dimerization is not required for enzymatic activation. Further studies are necessary to elucidate this mechanism.

Similarly, the role of the ATG2–18 complex in autophagy remains unclear, although its localization at the edge of the phagophore suggests involvement in the elongation and closure of the double membrane. The regulation of autophagy is also modulated downstream through the binding of different factors to the VPS34 complex. In particular, an inhibitory activity can occur through the binding of Beclin2 to Beclin1, while an enhancement of autophagy activity can be mediated by the binding of AMBRA1 to the VPS34 complex after being activated through phosphorylation by ULK1 [24]. The regulation of this nucleation step largely occurs through the phosphorylation of Beclin1 by several kinases. The activation of autophagy through Beclin1 phosphorylation is obtained through its modification at different sites. Specifically, MAP kinase‐activated protein kinase 2 (MAPKAPK2/3) regulation induced by stress targets Ser90, activating autophagy, as well as the modification at Ser15 by ULK1 that activates the VPS34 complex. Moreover, the modification of Beclin1 at Ser90 and Ser94 is obtained by AMPK upon a lack of glucose, allowing the activation of autophagy. Conversely, the suppression is achieved by its modification at Thr163 and Ser165. Ser90 is also dephosphorylated by protein phosphatase 2A (PP2A) in starvation conditions, restoring the activation of autophagy, which can also be achieved by phosphorylation of Beclin1 by death‐associated protein kinase (DAPK) at Thr119, impeding the binding to Beclin2. Finally, Beclin1 can be phosphorylated by epidermal growth factor receptor (EGFR) at Tyr229, Tyr233, and Tyr352 and by Akt at Ser295, allowing the suppression of autophagy. Other factors and proteins can be modified, allowing for the regulation of autophagy; among these, note that the modification of subunit ATG14L by mTORC1 at Ser3, Ser223, Ser233, Ser383, and Ser440, which mediate suppression of autophagy by impeding the formation of the active VPS34 complex [24].

The double layer of the phagophore can originate from various sources, including endoplasmic reticulum, plasma membrane, recycling endosomes, mitochondria, ATG9 vesicles, coat protein complex II (COPII) vesicles, and Golgi, ultimately forming the phagophore [21].

#### 1.3.3 Elongation and Maturation

The phagophore enlarges by wrapping around the cargo, adjusting its shape, and eventually internalizing the cellular debris, forming a double‐membrane autophagosome. Two conjugation systems are involved in the elongation step, namely conjugation system I and ATG8/LC3. The first conjugation system yields from the association to the phagophore membrane and dissociates after autophagosome complete formation. Conjugation system I is formed through an initial irreversible conjugation of subunits ATG12 and ATG5. This reaction is catalyzed by the E1‐like enzyme ATG7 and the E2‐like enzyme ATG10. After this step, ATG16L1 binds in a noncovalent manner to subunit ATG5, forming the dimerized final complex [21, 30]. This complex then associates with the forming phagophore membrane, where it works as an E3‐like enzyme, functioning as an E3‐like enzyme to facilitate the activation of the second conjugation complex, which in turn enables the elongation and curvature of the phagophore. The formation of the first conjugation complex is governed by various regulator proteins. Among these are the Golgi protein Ras‐related protein 33A (Rab33A), which binds to the ATG16L1 subunit and impedes the formation of the final complex, and the acetyltransferase lysine acetyltransferase 2B (KAT2B/p300), which inhibits the formation of the final complex through the acetylation of the three subunits [21].

The second conjugation system involved is the ATG8/LC3 system. The process begins with the proteolytic activation of LC3, primarily located in the nucleus, into LC3‐I by ATG4. ATG4 also performs the deconjugation step, releasing phosphatidylethanolamine (PE) to halt phagophore expansion. Afterward, lipidation (conjugation with the lipid PE) of LC3‐I into a membrane‐anchored LC3‐II is driven by ATG7 and ATG3, and conjugation complex I, acting as E1 and E2‐like and E3‐like enzymes, respectively [1], affording the expansion of the phagophore and closure of the membrane [31]. Enzymes ATG4 and ATG8 present various isoforms in humans. Four ATG4 isoforms and many ATG8‐like proteins have been identified as *human* paralogs. Among the ATG8 isoforms, the LC3‐I/II and GABA‐A receptor‐associated protein (GABARAP) subfamilies are noteworthy, since they contribute to different steps of autophagosome formation [21]. In addition to these ATG proteins, the transmembrane ATG9 is involved in the transportation of membrane material for the enlargement of the phagophore through the positive regulation mediated by ULK1 and VPS34 and the negative regulation mediated by mitogen‐activated protein kinase 14 (MAPK14/p38α) [21].

The elongation of the phagophore is regulated by ULK1, which inhibits the proteolytic activity of ATG4 through phosphorylation, or can be influenced by the presence of reactive oxygen species (ROS) in close proximity to the ER‐mitochondria contact sites. In addition to this, posttranslational modifications can negatively regulate the autophagic machinery. For instance, protein kinase A (PKA) inactivates LC3, and NUAK family kinase 2 (NUAK2) family and BR serine/threonine‐protein kinase 2 (BRSK2) protein kinase interfere with the formation of the phagophore.

#### 1.3.4 Fusion

The formed autophagosome is transported to its final destination through the cytoskeletal structure for fusion with the lysosome/endosome. During this step, all the ATG complexes involved in nucleation and elongation of the phagophore are released, and the machinery responsible for the lysosomal fusion is recruited. The recruitment of additional proteins and complexes includes the soluble *N*‐ethylmaleimide‐sensitive factor activating protein receptor (SNARE) superfamily proteins on the autophagosome (syntaxin17—STX17 and synaptosomal‐associated protein 29—SNAP29), on the lysosome (vesicle‐associated membrane protein 8—VAMP8), Rab GTPase family members, and the membrane tethering complex (homotypic fusion protein sorting complex—homotypic fusion and protein sorting [HOPS] complex) [11, 31], ultimately forming the fusion complex autolysosome.

#### 1.3.5 Degradation

The autolysosome is characterized by its acidic lumen and contains a range of hydrolytic enzymes that facilitate the degradation of engulfed cellular material. This degradation process results in the breakdown of the internal cargo into smaller molecules. The debris is then transported across the lysosomal membrane via lysosomal permeases into the cytoplasm, where it can be utilized for metabolic processes and energy production.

## 2 Autophagy and Viral Infections

Autophagy may either be triggered at some steps of the viral replicative cycle as a pathogenic infective mechanism or as a host defense mechanism in response to viral protein exposure, fighting the pathogenic invasion by sequestering the viral particles and virions for degradation and activating the host innate response [11]. As a matter of fact, induction can take place during viral adsorption through interactions between viral proteins and host membrane receptors (as cluster of differentiation 46 [CD46] in measles virus [MeV]), through interactions between viral envelope glycoproteins and the host membrane (during human immunodeficiency virus [HIV] entry), or after perturbation of the intracellular homeostasis caused by viral replication HCV ER‐stress induced mTOR pathway [11]. Nonetheless, some viruses have evolved mechanisms to counteract, evade, or even exploit the autophagic machinery for their own benefit, ultimately using it to support their replication and survival [11].

### 2.1 Autophagy Inhibition or Evasion

Given the defensive role of autophagy toward the host, some viruses have evolved the ability to inhibit the machinery through various pathways, as exemplified thereafter. Notably, a limitation of the initiation process through interactions with the type I PI3K‐AKT‐mTOR signaling pathway exists, as reported for Kaposi's sarcoma‐associated herpesvirus (KSHV). Further, inhibition of the type III PI3K‐VPS34‐Beclin1 pathway, as reported for HSV type 1 (HSV‐1), human cytomegalovirus (HCMV), and α‐herpesviruses (α‐HV) [11] may occur.

### 2.2 Induction and Exploitation of Autophagy

The autophagy machinery can be induced by some viruses at a later stage of the process to promote replication and propagation. Studies reported the exploitation of the double membrane vesicles (DMVs) for viral RNA synthesis, and some evidence confirm that this strategy is adopted by various species including *murine* hepatitis virus (MHV), Middle East respiratory syndrome coronavirus (MERS‐CoV), severe acute respiratory syndrome coronavirus (SARS‐CoV), SARS‐CoV‐2, HCV, and coxsackievirus B3 (CVB3). DMVs' induction and formation can have different origins other than from autophagosomes, which still need to be clarified. Some viruses also hijack other membranous vesicles, as lysosomes, for the egress from host cells, even though this process does not seem to be related to autophagy induction. Besides the initiation step of the machinery, viruses modulate the later stages of autophagy by interfering with the autophagosome–lysosome fusion step, resulting in an incomplete degradative mechanism and the accumulation of autophagosomes. To this end, viral proteins modulate several pathways, the prime example being the disruption of the lysosomal fusion mediated by enterovirus D68 (EV‐D68), rhinovirus type C (RV‐C), human parainfluenza virus type 3 (HPIV3), HCV, influenza A virus (IAV), KSHV, Epstein–Barr virus (EBV), and SARS‐CoV‐2. Ultimately, viruses harness this pathway to facilitate the maturation and release of viral particles, enabling the cell‐to‐cell spread of virions within vesicles that contain host receptors and proteins. This process helps the virus evade the host's innate immune response, promoting transmission to other cells within the same host and enhancing infectivity toward new hosts.

### 2.3 Autophagy–Virus Relationships Among Viral Families

The complex interplay between autophagy and viral infections, outlined in the previous paragraphs, reflects a dynamic “*hide and seek game”* between host defense mechanisms and viral survival strategies. As aforementioned, viruses may suppress, evade, induce, or exploit various stages of the autophagy pathway to favor their own replication and survival; these strategies are generally not limited to a single virus but are shared among diverse viral families. The following section highlights key virus families that exemplify these interactions, providing insights into the mechanisms of viral survival over autophagy modulation.

#### 2.3.1 Paramyxoviridae Family

Autophagy is induced in response to several viral infections; key species within this group include MeV [32, 33, 34], Newcastle disease virus (NDV), *Siniperca chuatsi* rhabdovirus (SCRV) [35], HPIV3 [36], bovine parainfluenza virus type 3 (BPIV3) [37], and peste de petits ruminants virus (PPRV) [38]. The induction of autophagy is achieved through different strategies. Among these are the activation of Beclin1 autophagy initiator (in BPIV3 infections [37]), and the autophagy induction during viral entry by host–virus protein interactions (in PPRV [39], MeV and NDV infections). In fact, during viral entry, MeV exploits the host complement regulatory molecule CD46 for binding to the envelope glycoproteins H and F in order to enter the host cell. The interaction between the fusion viral protein (F viral protein) and the host CD46 regulatory factor triggers the interaction with Golgi‐associated PDZ and coiled‐coil motif‐containing protein (GOPC) and induces the formation of the complex VPS34/Beclin1, thus inducing autophagy [11, 40, 41, 42]. Another strategy adopted by HPIV3 involves an early induction through the interaction with Tu translation elongation factor, mitochondrial (TUMF) and LC3 protein, mediating autophagosome formation [36] and a later induction of an abnormal accumulation of autophagosome, impeding the fusion with the lysosomes through interaction with the SNARE proteins (SNAP29 and STX17) and increasing the viral particles [43]. Interestingly, the activation mediated by NDV involves the induction of ER stress‐induced pathways, ultimately leading to the induction of immunogenic cell death. This mechanism has recently been investigated and exploited as an anticancer strategy in clinical trials, identifying NDV as an oncolytic virus [44, 45, 46].

#### 2.3.2 Retroviridae Family

Autophagy plays a controversial role in the life cycle of retroviruses, including HIV [47, 48, 49, 50]. On one hand, autophagy is induced by viral proteins as HIV‐negative regulatory factor (Nef) to exploit the autophagosomal membranes as a physical support for replication, and manipulating the machinery in later stages for viral assembly and egress, hindering at the same time the degradation of the newly formed virions [51]. On the other hand, autophagy plays a role in retrovirus inhibition by degrading viral components and activating host immune responses. Studies have focused on the induction of the PIK3K/mTOR pathway to suppress HIV replication [52, 53]. So, viruses have evolved to escape the antiviral effects of autophagy induction at various levels [54].

#### 2.3.3 Flaviviridae Family

Flaviviruses can initiate autophagy to offset virus‐induced stress, promote viral replication, and frequently initiate various stress responses in infected cells by creating double membrane structures that facilitate viral replication [55, 56]. HCV, Dengue virus (DENV), and Zika virus (ZIKV) [57, 58] induce autophagy to support the genome replication, while West Nile virus (WNV) growth seems to be independent from autophagy induction [59, 60, 61].

During HCV viral replication, autophagy is induced through multiple pathways, including the ER stress‐induced pathway. This latter is activated by the presence of viral proteins within the cellular cytoplasm, and it inhibits the mTOR pathway by inducing homeostasis through the unfolded protein response (UPR) and the AKT‐tuberous sclerosis complex (TSC) [11, 62]. Other mechanisms exploited by HCV and DENV include the promotion of lipidation of LC3 protein and the induction of ROS‐triggered phosphorylation of p62 through the ATF6, inositol‐requiring enzyme 1 (IRE1), or nuclear factor erythroid 2‐related factor 2 (Nrf2) pathways [11, 63, 64]. Nonetheless, HCV directly induces the formation of the autophagosome by interacting with various proteins or complexes, including Rab5, VPS34, ATG5, ATG10, and LC3 [11, 65]. HCV not only induces the autophagy machinery but also delays the fusion with the lysosomes at different steps through the deregulation of run domain Beclin‐1‐interacting and cysteine‐rich domain‐containing protein (RUBICON), UV radiation resistance‐associated gene protein (UVRAG), and UPR [66].

Other strategies exploited by DENV and ZKV involve the use of autophagosomal DMVs not only as a physical support for the genome replication, but also for the maturation of the particles and their egress from host cells. Studies have shown that DENV‐infected cells present the common viral envelope proteins (E viral proteins), capsid proteins, high mobility group box 1 protein (HMGB1), pre‐membrane protein/membrane protein (prM/M), nonstructural protein 1 (NS1), and the host LC3‐I and lipid droplets, whose presence is fundamental to promote replication and escape antibody neutralization, altering the host lipid metabolism as well [67, 68, 69]. Nonetheless, autophagy is also involved in the targeted degradation of DENV Ub‐dependently through the interaction of protein p62 with the capsid proteins, leading to inhibition of viral replication [70, 71].

#### 2.3.4 Herpesviridae Family

The negative modulation of autophagy by HSV is mediated through the interaction of several viral proteins; noteworthy are infected cell protein 34.5 (ICP34.5), US11, infected cell protein 0 (ICP0), insulin receptor substrate (IRS1), threonine‐tRNA ligase TRS1, and Akt‐like Ser/Thr kinase, with Beclin1 and ULK1 [11, 72, 73]. This type of autophagy modulation is also linked to the oncogenic potential of certain viruses belonging to this family, with the deregulation of this degradative pathway known to contribute to the development of some cancers. Reported modifications include the buildup of the cargo receptor sequestosome‐1 (p62/SQSTM1) with consequent cellular stress due to the interaction with Beclin1, the downregulation of Rab7 during the maturation step of the autophagosome, or the exploitation of mTOR1 for the virus protein synthesis, genome replication, and virion maturation [74, 75, 76, 77].

#### 2.3.5 Coronaviridae Family

Coronaviruses are known to induce autophagy to exploit the host DMVs, though the precise mechanisms of autophagosome induction still need to be elucidated, as well as the exact roles that the autophagy machinery plays in coronavirus replication, innate immune response, and disease pathogenesis [78]. Nonetheless, studies conducted on the MHV virus model demonstrate the induction of the autophagy machinery by coronavirus spp. favoring the viral replication. Even though some discrepancies have been reported, likely due to different viral strains and host cell types used [79], for example, studies have highlighted that in ATG5‐deficient cell lines in mouse models the viral replication is impaired when compared to studies conducted in embryonic stem cells expressing ATG5, confirming the importance of autophagy induction for viral replication [11]. However, other studies conducted in MHV‐infected primary murine embryonic fibroblasts document the irrelevance of the activation of the whole autophagy machinery, outlining a noncanonical utilization of LC3‐I acting as a coat protein rather than in its typical role in autophagosome formation [79, 80, 81].

Studies reported on SARS‐CoV infections have highlighted the enhancement of autophagosome formation and autophagy activation in host cells induced through various ways, among these are AMPK activation, mTOR inhibition, augmented levels of PI3P, and ER‐stress induction activation pathway, but also impairment of autophagosome maturation and fusion with lysosomes, mediated by either NSP6, papain‐like protease (PL^pro^) [82], open reading frame 8b (ORF8b), or PL^pro^‐TM [79]. Nonetheless, further clarifications on the connections between autophagy induction and SARS‐CoV replication are still required [83].

In contrast, literature suggests a negative regulation of autophagy by MERS‐CoV through autophagosome–lysosome fusion inhibition and a decrease in Beclin1 levels mediated through interference with the SNARE complex by NSP6 and PL^pro^‐TM [82]. Even though research has highlighted a growth in Beclin1 levels mediated by MERS‐CoV‐PL^pro^ similar to SARS‐CoV‐PL^pro^, the induction of autophagy in vitro upon treatment with kinase inhibitors targeting the VPS34, as well as the inhibition of the complex SKP2‐Beclin1, severely undermines MERS‐CoV replication [79, 84].

Finally, recent research suggests that SARS‐CoV‐2, similarly to SARS‐CoV, activates the autophagy machinery for its own exploitation during replication. This is achieved through mTORC1 inhibition, ER‐stress induced pathway, increased autophagosome formation with limited maturation [85], inhibition of the formation of VPS34 complex formation, inhibition of autophagosome–lysosome fusion [86], and lysosomal exocytosis, indicating the induction of an incomplete autophagy machinery. These mechanisms are triggered by SARS‐CoV‐2 nucleocapsid protein (viral N protein), NSP6, ORF8 protein, and ORF3a to hijack the autophagy machinery, to manage the autophagy‐induced inflammatory response, and to evade the host antiviral immunity [79, 87, 88, 89, 90, 91, 92].

#### 2.3.6 Picornaviridae Family

Studies have demonstrated the role of the Picornaviridae family in inducing autophagy through various pathways, including ULK1 activation. In particular, autophagosome membrane rearrangement is induced to provide a membranous scaffold for viral replication and a structural platform for the cytoplasmic delivery of mature virions and associated substances [93, 94]. As a matter of fact, research has shown that the inhibition of the autophagosome formation leads to both the inhibition of the viral RNA synthesis and the interference with the virus's life cycle, although a complete inhibition is not achieved since viral protein maturation and virion assembly may occur in compartments other than the DMVs. Nonetheless, picornavirus proteases also target the SNARE protein complex (examples are SNAP29 and pleckstrin homology and RUN domain containing M1 [PLEKHM1]) to block autophagy degradation.

Some EVs, specifically EV‐A71 or CVB3, also exploit the vesicles of the autophagy machinery to increase spread in the host by transferring entire clusters of viral particles in autophagosome‐like vesicles. Nonetheless, these vesicles can also be transmitted intact to the next host via fecal–oral routes, causing a more severe outcome [95, 96, 97, 98]. The regulation of this process is mediated by the inhibition of the mTORC1 by the viruses, upregulating autophagy and activating transcription factor EB (TFEB) that promotes viral release through secretory autophagy. Research papers report the induction of autophagy ad formation of autophagosomes in EV A71‐infected neuronal cells demonstrating the importance of this machinery for the viral replication [99], in accordance with studies conducted on EV71‐infected suckling mice [100], and as corroborated by the inhibition of the mitogen‐activated protein kinase/extracellular signal‐regulated kinase (MEK/ERK) pathway promoted by berberine, which downregulates the autophagy machinery [101]. The regulation of the autophagic machinery pursued by EV71 seems to be associated with Beclin1 regulation through interaction with the viral NSP 3D [102]. In addition to the exploitation of the host degradation pathways to carry on viral replication, EV71 also exploits the ERK signaling pathway to evade the immune system and regulate the production of interleukin‐6 (IL‐6) [103]. Different from EV71, CVB3 bypasses the ULK1/2 and the VPS34 complexes to induce autophagy. Instead, it initiates autophagy by the direct induction of the ubiquitination‐like conjugation system II [104].

#### 2.3.7 Orthomyxoviridae Family

Among the orthomyxoviruses, IAV induces autophagosome formation and prevents its maturation and fusion with the lysosome, managing an incomplete autophagy initiation to promote viral replication and virion assembly [105, 106, 107]. Studies reported the interaction of early‐ and late‐stage viral infection proteins, matrix protein 2 (M2), NS1, and the viral nucleoprotein (NP), with various autophagy signaling molecules and complexes [108, 109]. Among the involved mechanisms, noteworthy are the AKT/mTOR pathway, ATG16L1, Beclin1, impeding the fusion step, and the heat shock protein 90 (HSP90) alpha family class A member 1 expression [110, 111]. Moreover, the pharmacological inhibition of autophagy by modulating the VSP34 and mTOR pathways has been shown to reduce viral load and replication, confirming the crucial role of autophagy in viral infection [112, 113, 114, 115].

### 2.4 Crosstalk Between Autophagy and Immune Responses

As aforementioned, autophagy plays a crucial role in various immune responses, including the removal of invading pathogens, control of inflammation, antigen presentation, inflammatory cytokine secretion, and lymphocyte development [16, 31]. This is evident in autophagy defects correlated to autoimmune diseases, encompassing systemic lupus erythematosus, rheumatoid arthritis, psoriasis, diabetes, and multiple sclerosis [16]. The crosstalk between autophagy machinery and inflammatory signaling cascades is bidirectional, and autophagy can be activated and/or inhibited by type 1 Interferon (IFN‐I) [116] and Interferon γ [117], contributing to the innate antiviral immunity [118]. Autophagy also directly contributes to the elimination of invading pathogens through a mechanism known as xenophagy. Once entered within the cytosol, the invading pathogens are labeled with Ub chains and galectin, thus rendering them susceptible to selective autophagy [31].

## 3 Natural Products Modulating the Autophagic Machinery

Nature has historically served as a rich source of bioactive molecules, many of which have exhibited diverse pharmacological activities [119, 120, 121, 122]. Since these compounds represent the outcome of continuous evolutionary optimization, their structural complexity and chemical diversity offer several advantages in providing novel scaffolds for drug development, bypassing the synthetic challenges occasionally encountered with de novo‐generated libraries. The development and integration of advanced analytical techniques, such as genomics, metabolomics, and bioinformatics, facilitated the systematic exploration of natural reservoirs, unveiling previously unrecognized chemical entities and expanding the scope of potential drug candidates [123, 124]. In the context of autophagy modulation mediated by chemicals, a variety of natural products belonging to different structural classes have shown interesting properties in either the therapeutic activation or inhibition of the autophagic machinery [6, 125, 126]. Given the growing interest in autophagy modulation as a promising host‐targeting antiviral strategy for pathogen elimination, a curated list of phytocompounds with demonstrated antiviral activity through autophagy modulation is presented in Table 1 and thoroughly discussed in the following section.

**Table 1:** List of phytocompounds, their involvement in the autophagic machinery, and the viruses investigated for the overall activity on the modulation of autophagy.

Phytocompound	Overall activity on autophagy	Step	Autophagic modulatory mechanism	Viruses spp.	Ref.
**Terpenoids and steroids**					
(1) Wortmannin	Inhibitor	Nucleation	Inhibition of VPS34	IAV, ZIKV, USUV, MERS‐CoV, HCV	[127]
(2) Thapisagargin	Inhibitor	Fusion	Inhibition of autophagosome–lysosome fusion; Inhibition of the recruitment of Ras‐associated binding 7 (Rab7) protein	IAV, RSV, SARS‐CoV‐2, CoV229E, MERS‐CoV	[128, 129]
(3) Ursolic acid	Inducer	Initiation	Activation of Beclin‐1 and Akt/mTOR pathways	HBV	[130, 131]
(4) Celastrol	Dual activity: inducer and inhibitor	Fusion and degradation	Modulation of the ROS/JNK signaling pathway; destabilization of the androgen receptor through heat shock protein 90 β (HSP90β) inhibition or calpain activation: binding with vesicle‐associated membrane protein 7 (VAMP7) and Rab7 protein	HCV	[132, 133, 134]
(5) Cyclovirobuxine D	Inducer	Initiation	Regulation of the Akt/mTOR pathway	DENV ZIKV CVB3	[135, 136, 137]
**Nucleosides**					
(6) Tunicamycin	Inducer	Initiation	Increase in ER stress	HBV	[138, 139, 140]
**Saccharide**					
(7) Trehalose	Inducer	Initiation	Induction of mTOR‐independent autophagy	HRV‐16 HCMV HIV‐1	[141, 142, 143, 144]
**Glycosides**					
(8) Saikosaponin D	Leads to incomplete autophagy	Fusion	Inhibition of autophagosome–lysosome fusion	EV71	[145]
(9) Baicalin	Inducer	Initiation	Suppression of the mTOR signaling pathway	H3N2 CHIKV	[146]
(10) Pentagalloylglucose	Inducer	Initiation	Suppression of the mTOR signaling pathway	HSV‐1 IAV	[105, 147, 148]
(11) Tannic acid	Inducer	Initiation and nucleation	Modulator of the TLR4/NF‐κB and PI3K‐AKT‐mTOR pathways	HBV	[149]
(12) Apigetrin	Inducer	Nucleation	Increased conversion of LC3‐I to LC3‐II and promotion of autophagosome formation	IAV	[150]
(13) Ciliatoside A	Inducer	Initiation	Phosphorylation of AMPK and ULK1 and inhibition of mTOR phosphorylation	HBV	[151]
(14) Quercetin‐7‐*O*‐glucoside	Inhibitor	n.d.	Mechanism still to be elucidated	IAV IBV	[152]
**Flavanoids and Flavonoids**					
(15) Procyanidin	Inhibitor	Fusion	Inhibition of autophagosome accumulation	IAV	[153]
(16) Dihydromyricetin	Inducer	n.d.	Activation of NF‐κB and mitogen‐activated protein kinase (MAPK) signaling pathways	HBV	[154]
(17) Kurarinone	Inhibitor	n.d.	Mechanism still to be elucidated	HCoV‐OC43	[155]
(18) Kaempferol	Inducer	n.d.	Mechanism still to be elucidated	ASFV	[156]
(19) Deguelin	Inhibitor	Initiation and nucleation	Downregulation of Beclin1 expression	HCV	[157]
**Polyphenols**					
(20) Epigallocatechin‐3‐gallate	Inducer of complete autophagy	Nucleation and fusion	Upregulation of the AMPK signaling pathway; increased autophagosome formation and lysosomal acidification	HBV	[158]
(21) Deoxyshikonin	Inhibitor			RV	[159]
(22) Gallic acid	Inducer of complete autophagy	n.d.	Mechanism still to be elucidated	H1N1	[160]
(23) Urolithin A	Inducer	n.d.	Mechanism still to be elucidated	EV71	[161]
**Phenols**					
(24) Eugenol	Inhibitor	Initiation	Inhibition of the dissociation of Beclin 1 from BCL2	IAV	[162]
**Phenolic acids**					
(25) Lithospermic acid	Inducer of complete autophagy	Initiation	Increase of LAMP1 and LAMP2 expression impeding mTOR and AKT phosphorylation	HBV	[163]
(26) Rhein	Inhibitor	n.d.	Suppression of the TLR4, Akt, p38, JNK MAPK, and NF‐κB pathways	IAV	[164]
(27) Caffeic acid	Inhibitor	n.d.	Activation of the (p62)‐mediated autophagy	HCV	[165]
(28) Mycophenolic acid	Inhibitor	Initiation	Downregulation of the expression of key autophagy‐related genes	HCV DENV	[166, 167]
**Alkaloids**					
(29) Berberine	Dual effect: inducer and inhibitor	Nucleation	Modulation of the NF‐κB pathway; activation of AKT protein and JNK; inhibition of the phosphorylation of PI3K‐III	HSV‐1 EV71 IAV RSV	[168, 169]
(30) Berbamine	Inhibitor	Elongation	Inhibition of the binding of LC3 to LAMP1 BCL2‐interacting protein 3 (BNIP3)‐mediated autophagy mechanism	BVDV SARS‐CoV‐2	[170, 171]
(31) Evodiamine	Inhibition	Elongation	Inhibition of the accumulation of LC3‐II and p62; Inhibition of EFGP‐LC3 aggregation	IAV	[172]
**Biogenic amines**					
(32) Spermidine (33) Spermine	Inducer		Inhibition of Raf phosphorylation	SARS‐CoV‐2	[173]
(34) Melatonin	Inhibitor	Initiation	Inhibition by oxidative stress and ER stress reduction	RHDV JEV	[174, 175]
**Macrocycles**					
(35) Bafilomycin A1	Inhibitor	Fusion	Inhibition of the fusion of the autophagosome with the lysosome	ZIKV EBV SARS‐CoV‐2	[176, 177, 178]
(36) Rapamycin	Inducer	Initiation	Inhibition of mTOR pathway	TGEV ZIKV	[179, 180]

### 3.1 Terpenoids and Steroids

#### 3.1.1 Wortmannin

**Figure 3:**
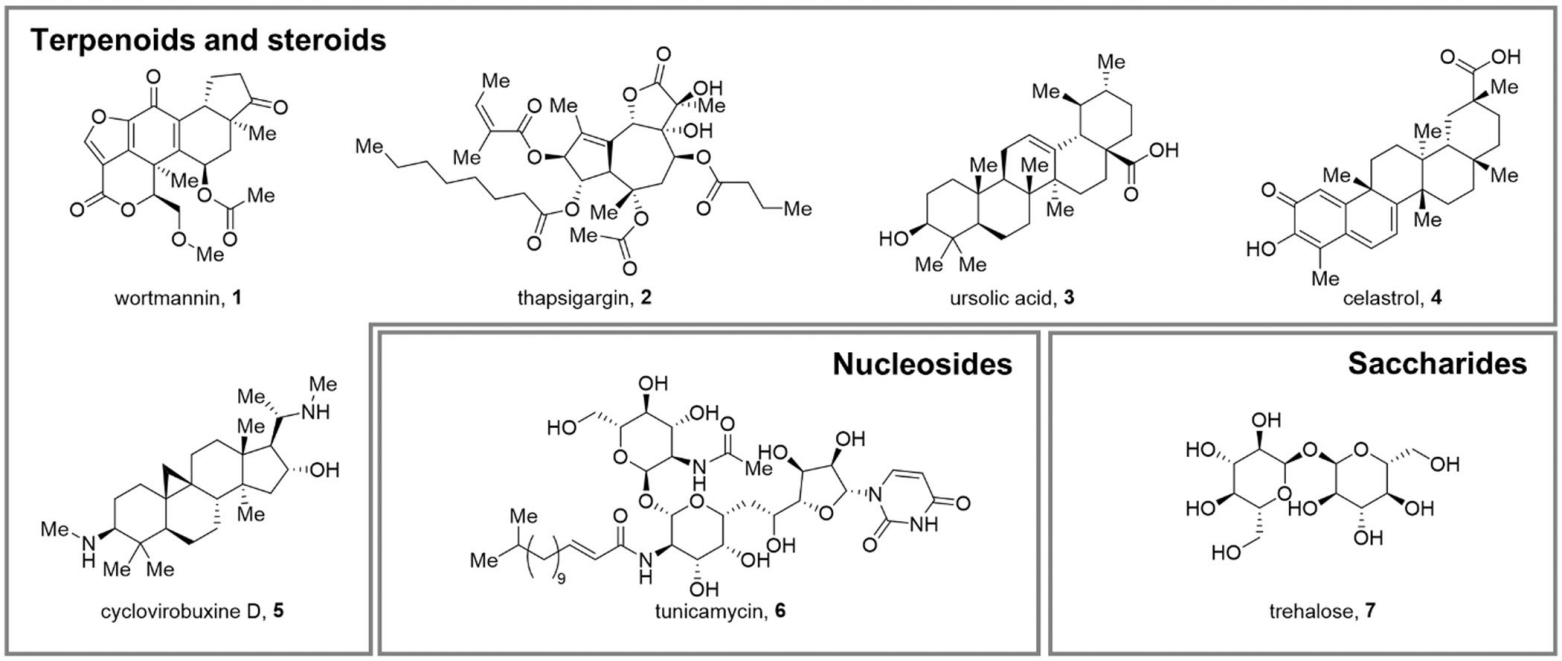
Chemical structures of the reported terpenoids, steroids, nucleosides, and saccharides **1‐7**.

Wortmannin (**1**, Figure 3) is a fungal metabolite isolated from *Penicillium funiculosum* and *Talaromyces wortmannii* [181]. This natural compound has garnered widespread interest owing to its potent inhibitory action on class III PI3K, displaying an IC_50_ of 4.2 nM. Exerting its effect through irreversible covalent modification of the catalytic subunit of PI3K, it can disrupt the autophagic cascade [127]. The compound's ability to hinder autophagic flux has been harnessed in diverse experimental settings to elucidate its fundamental autophagic mechanisms and investigate its implications in disease states, such as neurodegenerative disorders and cancer [182, 183]. The initial assessment of its inhibitory effects on autophagy in rat hepatoma cells not only underscored its effectiveness but also provided foundational insights into its mode of action [184]. Further corroboration of **1**'s utility in autophagy research is evident in Petiot et al.'s works, wherein **1** was exploited to decipher the involvement of class III PI3K in autophagosome formation [185]. Additionally, more recent investigations by Mizushima and Yoshimori have utilized **1** to decipher autophagic machinery dynamics [186]. Building on these foundations, **1** has now become a widely used reagent in a variety of biological assays, as either positive control or pretreatment to block autophagy. The pivotal discovery of the autophagy‐dependent nature of IAV infection has been proven by employing **1** as an autophagy inhibitor, evidencing that IAV‐induced autophagosome formation and autophagic flux are decreased in the presence of **1** [187]. In agreement with these findings, **1** was found capable of reducing the conversion of LC3‐I to LC3‐II in ZIKV‐infected human umbilical vein endothelial cells, thus reducing the viral progeny formation [188]. The same conclusions were drawn by Blázquez et al. while investigating the Usutu flavivirus infection in Vero cells and MERS‐infected hepatoma Huh7 cells [189, 190]. Compound **1** has also been exploited for the development of functionalized semisynthetic derivatives designed as pharmacological tools or probes. Of note, sonolisib (PX‐866) is a **1** analog showing improved pharmacodynamic and pharmacokinetic profiles, with its sub‐nanomolar IC_50_ against PI3K, lower cytotoxicity, higher biological stability, and oral bioavailability [191]. In addition, an activity‐based protein profiling (ABPP) clickable probe based on **1**, called wortmannin‐yne, was engineered to conduct kinase activity profiling studies in HCV‐infected Huh7.5 cells. The alkyne‐bearing probe undergoes biorthogonal click chemistry reactions in situ to label active kinases, enabling the elucidation of the HCV‐mediated kinome modulation [192].

#### 3.1.2 Thapsigargin

Thapsigargin (**2**, Figure 3) is a guaianolide belonging to the sesquiterpene lactones, isolated from *Thapsia garganica* roots. Along with the plant's traditional therapeutic application as an emetic or anti‐rheumatic, the skin irritant and cytotoxic properties of **2** have propelled drug discovery research, prompting further exploration into its underlying mechanism of action. Compound **2** was identified as a nanomolar‐potent sarcoplasmic/endoplasmic reticulum Ca^2+^ ATPase (SERCA) inhibitor (IC_50_ = 10–20 nM). The depletion of ER calcium stores, resulting in increased cytosolic calcium, triggers ER stress and activates the UPR. This protective mechanism halts protein translation, promoting the degradation of misfolded proteins and enhancing chaperone synthesis. In a pivotal study from 2011, **2** was found to not only induce ER stress but also act as an autophagy modulator. Indeed, it was able to hinder autophagosome‐lysosome fusion by impeding the recruitment of Rab7 protein, a critical factor for complete autophagic flux. **2**'s inhibitory effect on autophagy was proven to be unrelated to its impact on ER stress, as confirmed by the **2**‐induced inhibition of autophagic flux in mouse embryonic fibroblasts (MEF) cells deficient in the ER stress response, resulting from the genetic deletion of both isoforms of IRE‐1 (α and β) [128, 129]. Since then, **2** has been used in experimental settings as both ER stress inducer and autophagy inhibitor [193, 194]. Compound **2** also emerged as a significant host‐targeting antiviral against major human respiratory viruses (i.e., IAV, RSV, CoV‐OC43, and SARS‐CoV‐2) in human cells, surpassing the performance of remdesivir and ribavirin. Unlike traditional antivirals, **2** prompted a robust and extended antiviral response by inducing ER stress and UPR, activating various host defenses, including an enhanced type I/III interferon response. In mouse models, oral administration of **2** protected against lethal influenza virus infection, both pre‐ and postinfection [195, 196]. During the Coronavirus Disease 19 (COVID‐19) pandemic, Shaban et al. discovered that **2** inhibits the replication of autophagy‐dependent viruses, namely hCoV‐229E, MERS‐CoV, SARS‐CoV‐2, and IAV strain KAN‐1 in different cell lines. Proteome‐wide analyses identified key pathways and proteins affected by **2**, including essential ER quality control factors (HERPUD1), novel factors (Ub‐like modifier activating enzyme 6 [UBA6] and zinc finger protein [ZNF622]), and the autophagy regulator p62/SQSTM1. Compound **2** suppressed viral replication, impeding CoV‐induced selective autophagic flux by countering CoV‐induced suppressive effects on ER functions (e.g., increased levels of binding immunoglobulin protein [BiP], HERPUD1, and IRE‐1α). The authors attributed **2**'s sustained impact on blocking CoV‐mediated autophagic flux to the inhibition of autophagosome–lysosome fusion by upregulating p62/SQSTM1 and LC3B levels. Indeed, **2** induced a reprogramming of metabolic pathways and a dedicated network of proteins involved in ER stress, ER quality control, ER‐associated degradation, and autophagy. These effects underscore **2**'s potential as a therapeutic autophagy modulator against CoV infections [197, 198]. Building on this groundwork, the future exploration and development of **2** derivatives might result in an optimized family of potent antiviral compounds, thereby presenting a potential strategy for significant advancements in antiviral therapeutics. In parallel, anticancer drug discovery is actively harnessing the cytotoxic potential of **2** as mipsagargin, a peptide‐masked prodrug derivative of **2**, has entered clinical trials, demonstrating promising prospects for treating glioblastoma [199, 200, 201].

#### 3.1.3 Ursolic Acid

Ursolic acid (**3**, Figure 3) belongs to the triterpenoid family and is widely found in apples and other fruit peels, herbs, and spices. Its anticancer activity has been explored in liquid and solid tumors and can be ascribed to the inhibition of DNA replication and the induction of apoptosis and/or autophagy. It is noteworthy that **3** induces autophagy by regulating specific pathways, depending on the cell line. In PC3 prostate cancer cells it activated the Beclin‐1 and Akt/mTOR pathways [130], while in U87MG glioblastoma cells it was found to regulate the phosphorylated ERK/eukaryotic initiation factor 2α/C/EBP homologous protein (PERK/eIF2α/CHOP), calmodulin‐dependent kinase protein kinase (CaMMK)/AMPK/mTOR, and inositol‐requiring enzyme α (IRE‐1α)/Jun N‐terminal kinase (JNK) signaling pathways [131]. The autophagy modulation potential of **3** in infected cells has been proven in *Trypanosoma cruzi* infections but it has not been thoroughly investigated amidst viral infections yet. Nonetheless, a study on **3** highlighted its protective effect in HBV‐infected hepatoma cells by reducing HBV X protein (HBx)‐induced autophagy, while also reversing the associated anticancer drug resistance.

#### 3.1.4 Celastrol

Celastrol (**4**, Figure 3) is a pentacyclic nortriterpene quinone found in *Tripterygium wilfordii* and *Tripterygium regelii* root extracts, sharing structural similarities with **3**. Evidence highlighted its antibacterial, antioxidant, anti‐inflammatory, and anticancer activities [202]. In recent years, it has also proved to be one of the most potent antiobesity agents discovered so far, thus attracting the scientific community's interest [203]. Compound **4** can act as both an inducer and an inhibitor of autophagy, depending on the experimental conditions. In human glioma cells, it triggered apoptosis and autophagy by modulating the ROS/JNK signaling pathway, and in prostate cancer cells, it induced autophagy by destabilizing the androgen receptor through HSP90β inhibition or calpain activation [132, 133]. Extensive studies on osteosarcoma cells showcased a similar effect and were confirmed in a mouse xenograft model, which showed inhibition of tumor growth [204]. On the other hand, **4** is able to inhibit autophagy in preadipocytes, causing increased apoptosis by activating the mitochondrial‐mediated pathway. It inhibited the fusion of the autophagosome and lysosome by binding with VAMP7 and Rab7 protein [205]. In HCV‐triggered hepatocellular carcinoma, **4** was found to inhibit HCV translation by targeting the ATPase pocket of HSP90β, thus hampering internal ribosomal entry site‐mediated translation [206]. To date, the potential relationship between **4** and autophagy in the context of viral infections remains unclear, warranting further comprehensive investigation to elucidate the mechanisms underlying their interplay.

#### 3.1.5 Cyclovirobuxine D

Derived from the root of the medicinal plant *Buxus microphylla*, cyclovirobuxine D (**5**, Figure 3) is a steroidal alkaloid known for its positive impact on cardiovascular health [207, 208]. It is known to uphold beneficial effects in heart failure, arrhythmias, and myocardial ischemia [209, 210, 211]. Additionally, **5** demonstrated promising anticancer properties, inducing mitochondria‐mediated apoptosis in various cancer cells and triggering autophagy‐associated cell death by influencing the Akt/mTOR pathway in human breast cancer cells [135, 136]. This natural compound not only inhibited DENV replication in vitro in a dose‐dependent manner but also protected suckling mice from lethal DENV infection. Mechanistically, it has been shown that **5** modulated genes are associated with the cellular cholesterol pathway, enhancing cellular cholesterol synthesis and activating mTOR, thus impeding virus‐dependent autophagy. Notably, **5** also inhibited the replication of other positive‐strand RNA viruses, including ZIKV and CVB3 [212]. Building on these foundations, the development of semisynthetic derivatives could represent a promising broad‐spectrum antiviral strategy against positive‐strand RNA viruses dependent on autophagy for optimal replication.

### 3.2 Nucleosides

#### 3.2.1 Tunicamycin

Tunicamycin (**6**, Figure 3) can be described as a mixture of homologous nucleoside antibiotics (i.e., Tunicamycin A, B, C, and D) produced by several *Streptomyces* spp. Compound **6** was discovered to inhibit protein glycosylation through the inhibition of *N*‐acetylglucosamine‐1‐phosphate transferase. Hampering the synthesis of glycosylated proteins, it exerts an antibacterial and antifungal effect by disrupting the cell wall biosynthesis and may cause G1‐phase cell cycle arrest in human cells. Regarding viral infections, it impedes the glycosylation of viral proteins, thus reducing viral spread [213]. Altered protein glycosylation also leads to the accumulation of unfolded protein in the ER, ultimately increasing ER stress. Compound **6** is often employed in biological assays to either investigate the role of glycosylation in different cellular models or as an ER stress inducer. It is known that ER stress‐induced autophagy becomes relevant to guarantee cell survival when the primary ER‐associated protein degradation (ERAD) pathway gets saturated [138, 139]. Moreover, **6**‐induced autophagy in human cells has been thoroughly reported in the literature [128, 139, 195, 214, 215, 216]. The therapeutic potential of **6** as an autophagy modulator in viral infection has not been thoroughly explored yet. To date, only the “pro‐viral” effect of **6** on HBV‐infected HepG2.2.15 cells has been reported, in which **6** induced autophagosome formation while inhibiting autophagosome–lysosome fusion. As HBV infection is acknowledged to enhance ER stress and autophagy to secure viral replication and spreading, **6** seems to aid virus‐loaded exosome release [140]. Further investigations are needed to understand the properties of **6** in other types of infection.

### 3.3 Saccharides

#### 3.3.1 Trehalose

Trehalose (**7**, Figure 3) is a nonreducing glucose disaccharide found in plants, insects, and microorganisms. Spanning across various industries, the primary applications of **7** are as a food additive, a moisturizing agent in cosmetic formulations, and a key component in ophthalmic drops designed for the effective treatment of dry eye syndrome. Its widespread use is justified by its minimal cytotoxicity in a variety of mammalian cells [217]. This natural compound has also been acknowledged as an mTOR‐independent autophagy inducer for its ability to promote the recruitment of LC3‐II and the subsequent autophagosome formation, presumably via modulation of the AMPK/ULK1 pathway [218, 219]. According to Wu et al. **7**‐induced autophagy directly inhibited the expression of the antiviral interferon lambda 1 (IFN‐λ1), thus promoting HRV‐16 infection in normal human tracheobronchial epithelial cells [220]. Contrastingly, supplementary experimental data proposed the potential therapeutic efficacy of **7** against various viruses, presenting a promising alternative in infections, altering the sensitivity in mTOR kinase‐containing complexes to inhibitors. For instance, a study was conducted on HCMV‐infected *human* foreskin fibroblasts, *human* aortic endothelial cells, and neural cells derived from *human* embryonic stem cells. In the examined cell types, **7** triggered autophagy and suppressed HCMV gene expression, significantly impeding the viral spread [143]. More recent evidence revealed that **7** inhibits intracellular replication and viral entry of HIV‐1 infection in primary human macrophages and CD4^+^ T lymphocytes. The underlying mechanisms encompassed mTOR‐independent induction of autophagy, coupled with the downregulation of C‐C chemokine receptor type 5 (CCR5) in T cells and CD4 in both T cells and macrophages [221]. Since **7** has also garnered attention for its autophagy‐related protective effects against cardiovascular diseases, its potential was explored in the context of CVB3‐induced acute viral myocarditis. Compound **7** alleviated myocardial injury and inflammatory reactions in mice VMC‐B cells, by activating the AMPK/ULK1 signaling pathway, triggering autophagy [219]. The reported findings contribute to emphasizing the potential of **7** in the treatment of viral infections and virus‐related diseases.

### 3.4 Glycosides

#### 3.4.1 Saikosaponin D

**Figure 4:**
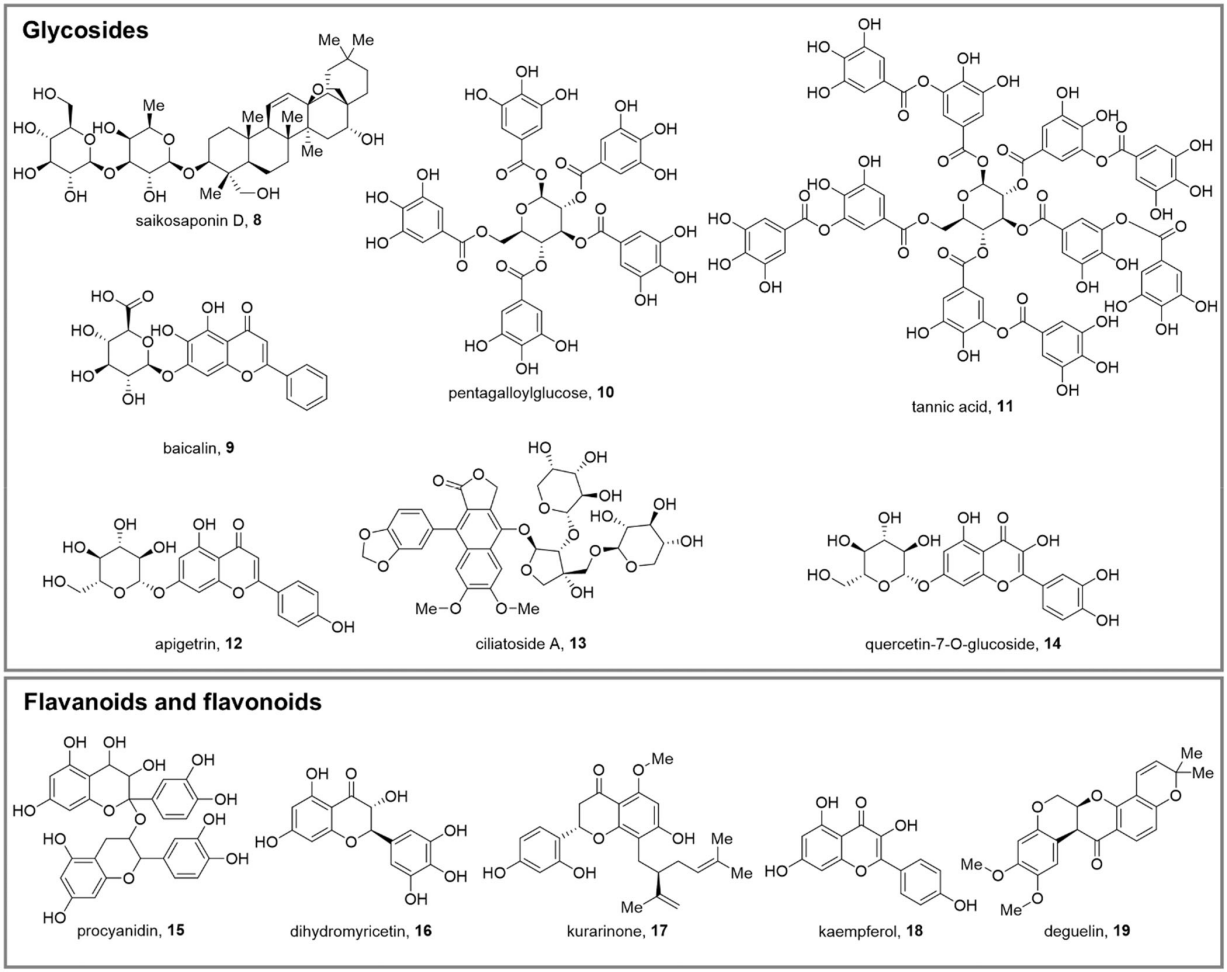
Chemical structures of the reported glycosides, flavonoids, and flavonoids **8–19**.

Saikosaponin D (**8**, Figure 4) is a triterpenoid saponin glycoside, isolated from *Bupleurum kunmingense* and *Bupleurum falcatum*. Alongside its application in traditional Chinese medicine for the treatment of depression, it emerged as an interesting anticancer agent and, notably, as a promising autophagy modulator [222, 223, 224]. In this context, it proved to effectively block autophagic degradation in MDA‐MB‐231 triple negative breast cancer cells by inhibiting autophagosome–lysosome fusion, leading to cell death and halted proliferation. The results of the study confirmed that treatment with **8** had no impact on the lysosomal acidic environment and lysosome‑associated membrane glycoprotein 1 and 2 (LAMP1 and LAMP2), but it could inhibit autophagosome‐lysosome fusion, disrupting co‐localization of LC3 and LAMP1. Moreover, there was no correlation between autophagy inhibition and **8**‐mediated apoptosis [225]. Further testing revealed that **8** also displayed antiproliferative effects on RG‐2 and U87‐MG glioma and U251 glioblastoma cell lines. The mode of action was clarified, as evidence suggested that **8** induced DNA damage within glioblastoma multiforme cells, triggering both apoptosis and autophagy through the activation of the ER stress‐signaling pathway [226]. Pertaining to the field of viral infections, triterpenoid saponins' autophagy modulation potential was explored in the treatment of hand‐foot‐mouth disease, exploiting EV71‐infected HeLa cells. Treatment with **8** or saikosaponin A inhibited autophagosome–lysosome fusion, leading to autophagosome accumulation. Additionally, the treatment increased lysosomal pH, induced nuclear localization of TFEB, and was found to require Rab5 for autophagosomal–lysosomal fusion blockage. Furthermore, the treatment significantly reduced EV‐A71 RNA replication, viral protein synthesis, and virus titers, correlating with inhibitory effects on autophagy. Notably, **8**‐induced LC3‐II puncta failed to co‐localize with lysosomal markers but exhibited strong co‐localization with autophagy reporter p62. The study also suggests that **8** and saikosaponin A may indirectly activate Rab5, contributing to lysosomal defects [145].

#### 3.4.2 Baicalin

Baicalin (**9**, Figure 4) is a flavone glycoside extracted from *Scutellaria baicalensis*, *Scutellaria lateriflora*, and *Scutellaria galericulata*. In traditional Chinese medicine, it is employed for its anxiolytic effects due to the positive allosteric modulation of the GABA‐A receptor [227, 228]. It also exerts antiviral, antiapoptosis, anti‐inflammatory, and antioxidative properties, while displaying low toxicity [229, 230, 231, 232]. Based on the idea of autophagy modulation as a strategy to fight viral infection, its potential was investigated to fight IAV infections, in which it inhibited H3N2‐induced autophagy by suppressing mTOR signaling in A549 and Ana‐1 cells. An initial significant increase in the number of autophagosomes in H3N2‐infected cells was observed, which was thwarted by treatment with **9** [146]. In another study, Oo et al. suggested a potential use of **9** to treat chikungunya virus (CHIKV) infections. Compound **9** both hampered the early stages of CHIVK infections and had potent prophylactic effects. The authors ascribed these effects to several mechanisms, including the blocking of endosome acidification resulting in a pronounced autophagic inhibitory effect, interactions with multiple CHIKV proteins, inhibition of replication complex, and interference with amplification of CHIKV positive‐strand RNAs. For the former, a downregulation of CHIVK‐mediated autophagy in HEK‐LC3 cells was reported. The authors observed a reduction in the number of LC3 puncta (indicative of autophagy) in CHIKV‐infected cells upon treatment with **9** compared to untreated infected cells. Western blot analysis confirmed decreased LC3‐I to LC3‐II conversion in **9**‐treated cells compared to untreated infected cells. Since autophagy is crucial for CHIKV replication and release, inhibiting it with **9** could restrict virus yield. Therefore, **9** downregulation of infection‐associated autophagy may limit further CHIKV replication and release from infected cells [233].

#### 3.4.3 Pentagalloylglucose

Pentagalloylglucose (**10**, Figure 4) is one of the most potent antioxidants among tannins, displaying antimicrobial, antidiabetic, anticancer, and anti‐inflammatory effects [234]. Considerable efforts were made to elucidate the mechanism underlying its broad‐spectrum antiviral activity [235, 236, 237, 238]. Among them, the link between the antiviral activity of **10** and autophagy modulation has been elucidated. Preliminary work in this field investigated an in vitro model of HSV1‐infected MRC‐5 fibroblasts, highlighting that **10** acted as an autophagy inducer by suppressing the mTOR signaling pathway, hence increasing autophagosome formation in a dose‐ and time‐dependent manner. This led to HSV‐1 virions' xenophagy and weakened infection spreading [147]. Following studies by the same group revealed that treatment with **10** boosted IAV‐induced autophagosome formation but inhibited their aggregation. These effects are reflected by the increased levels of green fluorescence protein (GFP)‐LC3 fluorescence puncta and reduced levels of aggregation. It was inferred that IAV strategically utilizes autophagy to boost the accumulation of essential viral components. This process has been related to modifications in Hsp90 induction and the mTOR/p70S6K signaling pathway [105]. Furthermore, the investigation into compound **10**'s antiviral mechanisms in RABV‐infected Kunming mice revealed its ability to suppress viral adsorption and entry. Notably, **10** also demonstrated a significant hampering of the autophagic process, leading to the inhibition of rabies virus (RABV) replication. This effect involved decreased LC3‐II levels and upregulation of the mTOR pathway. Inhibiting mTOR diminished **10**'s anti‐RABV efficacy, confirming mTOR's key role in **10**‐induced autophagy inhibition [148].

#### 3.4.4 Tannic Acid

Tannic acid (**11**, Figure 4), also known as gallotannic acid, is another example of a natural, widely occurring tannin. This compound exhibited antiviral activity by inhibiting viral proteases and disrupting the adsorption of the virus to host cell membranes. In a recent study on HBV‐expressing HepG2.2.15 cells, **11** suppressed HBV replication by activating the Toll‐like receptor 4 (TLR4)/NF‐κB and PI3K‐AKT‐mTOR pathways. Compound **11**‐induced activation of autophagy was linked to improved HBV clearance. Taken together, this evidence sheds light on the potential of tannins as autophagy modulators for the treatment of viral infections [149].

#### 3.4.5 Apigetrin

Apigetrin (apigenin‐7‐*O*‐glucoside) (**12**, Figure 4) is among the main active ingredients of *Ixeris sonchifolia* and *Teucrium gnaphalodes*. In traditional Chinese medicine, it is often employed to treat cardiovascular diseases and other inflammation‐related pathologies [150, 239, 240, 241]. Recently, in a human TLR4 promoter dual‐luciferase reporter assay system to screen ethnomedicines, **12** was selected based on its antiviral potential against IAV for further investigations. Experiments revealed that IAV infection increased the expression of autophagy genes, enhancing the conversion of LC3‐I to LC3‐II and promoting autophagosome formation. This did not result in complete autophagy, as the expression of IAV M2 protein impeded autophagosome–lysosome fusion, leading to autophagosome accumulation and autophagic flux blockade. In A549 cells transfected with an EGFP‐LC3 plasmid, treatment with **12** significantly inhibited IAV‐induced autophagosome accumulation, as evidenced by reduced fluorescence puncta. Compound **12** also diminished the conversion of LC3‐I to LC3‐II and the accumulation of p62, suggesting its role in reducing IAV‐triggered autophagosome accumulation and enhancing autophagic flux [242].

#### 3.4.6 Ciliatoside A

The lignan glycoside ciliatoside A (**13**, Figure 4) has been isolated from *Peristrophe japonica*, after finding that the plant ethanolic extract had a significant anti‐HBV effect, remarkably reducing HBsAg in the supernatant of HepG2.2.15 cells. Compound **13** demonstrated significant anti‐HBV activity without inducing toxicity in HBV‐infected cells and HBV recombinant‐cccDNA mice. The compound notably reduced HBsAg expression and cccDNA transcriptional activities. Mechanistic insights revealed that **13** induced the autophagy–lysosome pathway through AMPK/ULK1/mTOR axis regulation, leading to the inhibition of HBV transcription and replication. Further experiments demonstrated that **13** activated autophagy by promoting phosphorylation of AMPK and ULK1 while inhibiting mTOR phosphorylation in a dose‐dependent manner [151].

#### 3.4.7 Quercetin‐7‐*O*‐Glucoside

Quercetin stands out as one of the most ubiquitous flavonoids, as it is present in numerous fruits and vegetables. It can be found in nature both as aglycon or as one of the many flavonoid glycosides—such as rutin and quercitrin, with the latter usually being more bioavailable. In all forms, quercetin is suggested to exert antioxidant, anti‐inflammatory, antimicrobial, and antiviral effects [243, 244, 245, 246, 247]. In the framework of investigating its therapeutic potential through autophagy modulation, numerous studies have specifically examined its cytotoxic impact on oncovirus‐induced cancers via in vitro experiments, suggesting that quercetin can modulate the autophagic machinery [248, 249, 250]. As for the scope of this review, a study on its glycoside quercetin‐7‐*O*‐glucoside (**14,** Figure 4) revealed that its antiviral properties against IAV and IBV viruses are related to the interaction with viral RNA polymerase, blocking viral RNA synthesis. In addition, treatment with **14** significantly reduced ROS and hampered influenza virus‐induced autophagy. In influenza virus‐infected Madin‐Darby canine kidney (MDCK) cells, **14** effectively prevented the formation of acidic vesicular organelles (AVOs), indicative of autophagy, and normalized autophagy‐related gene expression, including ATG5, ATG7, and LCB‐3, to levels comparable to noninfected cells [152].

### 3.5 Flavanoids and Flavonoids

#### 3.5.1 Procyanidin

Catechin and epicatechin are prone to condensation. The oligomerization reaction yields condensed tannins known as procyanidin (**15**, Figure 4), which maintain suitable bioactivity as anti‐inflammatory agents and acceptable bioavailability. In 2012, Dai et al. designed a high‐throughput screening study on 83 medicinal plants employed in traditional Chinese medicine, in which 35 extracts were identified as anti‐IAV agents. Seven examples were not reported in the literature, and they comprise *Peucedanum praeruptorum*, *Lantana camara, Litsea cubeba, Ginkgo biloba, Vaccinium angustifolium, Vitis vinifera*, and *Cinnamomum cassia*. The latter 3 are known to be rich in **15**, hence their antiviral activity against IAV was thoroughly investigated. While **15** could not impede viral infection, it significantly inhibited IAV replication in a concentration‐dependent manner. The IAV‐induced increase in LC3‐II levels was reverted by **15**, confirming its inhibitory effect on autophagosome accumulation in MDCK cells. The results were also supported by the reduction of ATG5, ATG7, and ATG12 levels following treatment with **15**. Mechanistic investigations confirmed the inhibition of IAV‐induced autophagy, depicted by the reduced formation of the ATG5‐ATG12‐ATG16 trimer and the hampered dissociation of the Beclin1/B‐cell lymphoma 2 (BCL2) heterodimer [153].

#### 3.5.2 Dihydromyricetin

Also referred to as ampelopsin, dihydromyricetin (**16**, Figure 4) is a flavanolol extracted from *Ampelopsis, Pinus, Cedrus*, and *Salix* spp. [251]. While numerous studies have concentrated on assessing the antiviral properties of **16**, there is a limited body of research investigating its potential as a modulator of autophagy in the context of viral infections [252, 253, 254, 255]. Nonetheless, a study revealed a significant reduction in both HBV replication in HepG2.2.15 cells and secretion of HBsAg and HBeAg, following treatment with **16**. Notably, **16** suppressed the mRNA expression of various HBV RNAs. Concurrently, **16** increased the mRNA levels of inflammatory cytokines and antiviral effectors, while decreasing the expression of HNF4α, positively associated with HBV replication. Mechanistically, **16**'s inhibitory effects on HBV were attributed to the activation of NF‐κB and MAPK signaling pathways, alongside induced autophagy. The latter was detected by an increase of p62, Beclin‐1, and LC3‐II levels. The authors presumed that **16** might activate autophagy through an NF‐κB‐mediated pathway [154].

#### 3.5.3 Kurarinone

Kurarinone (**17**, Figure 4) is a prenylated flavanone isolated from *Sophora flavescens*. It displays a multifaceted pharmacological profile, showcasing both potent antioxidant and anti‐inflammatory effects while also demonstrating immunosuppressive and cytotoxic properties [256, 257]. The latter has been attributed to the activation of proapoptotic proteins through caspase‐dependent pathways. **17** was also found to sustain autophagic cell death by activating autophagy‐related proteins in *human* hepatocarcinoma cells. The COVID‐19 pandemic accelerated investigations into its antiviral potential. Notably, the research highlighted that **17** could inhibit the progression of hCoV‐OC43 infection in MRC‐5 lung cells in a dose‐dependent manner, with an IC_50_ of 3.458 ± 0.101 μM. **17** was demonstrated to interfere with virus‐induced autophagy, distinctly suppressing the virus‐induced cytopathic effect when compared to untreated cells. Although **17** could not impede viral entry, it disrupted HCoV‐OC43‐induced autophagy, leading to heightened expression of LC3‐I and LC3‐II, alongside the accumulation of p62/SQSTM1 protein. This disruption emerged as pivotal for **17** antiviral efficacy, especially during the initial infection stages, as the optimal effectiveness of **17** was observed when administered within 24 h of the initial infection. The induced p62/SQSTM1 protein emerged as a key component in inhibiting hCoV‐OC43 infection, mirroring its antiviral role observed in other viral infections. Further validation of this promising proof of concept in additional models and in vivo studies could unlock novel potential applications of **17** in clinical settings or prompt the development of optimized derivatives [155].

#### 3.5.4 Kaempferol

Kaempferol (**18**, Figure 4) is a naturally occurring flavonol, found in a plethora of dietary plants [258]. It is acknowledged in the medicinal chemistry field as a natural anti‐inflammatory and antioxidant agent, both in its aglycon and glycoside forms [259, 260, 261]. In a Vero cell‐based library screening involving 90 flavonoids conducted to assess their potential antiviral properties, nine compounds were identified as possessing antiviral activity against the ASFV Ba71V strain. Among these compounds, **18** emerged as the most potent inhibitor, displaying a dose‐dependent effect with a virostatic mechanism of action. Further investigations unveiled that **18** targeted both the entry and post‐entry stages of the African swine fever virus (ASFV) replication cycle. Moreover, it was discovered that **18** induced autophagy in ASFV‐infected Vero cells, establishing a correlation between autophagy induction and the antiviral activity of the compound. Importantly, this autophagic response was found to be partially alleviated by the addition of an autophagy inhibitor. Additionally, **18** exhibited a dose‐dependent inhibitory effect against a highly virulent ASFV Arm/07 isolate in porcine macrophages [156]. Further studies are necessary to enhance our understanding of its antiviral potential against human viruses, providing opportunities to investigate potential therapeutic applications.

#### 3.5.5 Deguelin

Sourced from legume barks, leaves, seeds, and roots, deguelin (**19**, Figure 4) demonstrates notable anticancer activity by triggering AKT/protein kinase B (PKB)‐mediated apoptosis and autophagy in carcinoma cells [262, 263, 264]. Liao et al. explored the antiviral potential of **19** in modulating autophagy in viral infection settings. HCV JFH‐1‐infected Huh7 cells treated with **19** exhibited a notable reduction in the number of GFP‐LC3‐positive dots—signifying autophagy suppression, resulting in significant dose‐ and time‐dependent inhibition of HCV JFH‐1 replication. This anti‐HCV activity was attributed to the downregulation of Beclin1 expression, a pivotal factor in autophagosome formation. Treatment with **19** inhibited the conversion of LC3B‐I to LC3B‐II and restrained the formation of GFP‐LC3 puncta, accompanied by an increase in p62 levels. Further mechanistic insights revealed that the modulation of Beclin1 played a crucial role, as overexpression or silencing of Beclin1 in **19**‐treated cells weakened or enhanced the inhibitory effect on autophagy, respectively. Hence, this study suggested that **19** anti‐HCV activity is linked to its ability to suppress cellular autophagy through Beclin1 downregulation [157].

### 3.6 Polyphenols

#### 3.6.1 Epigallocatechin‐3‐Gallate

**Figure 5:**
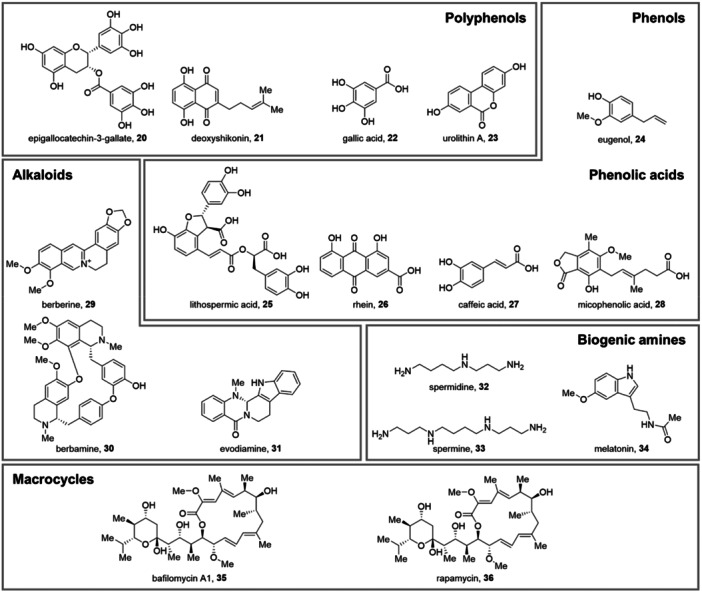
Chemical structures of the reported (poly)phenols, phenolic acids, alkaloids, biogenic amines, and macrocyclic compounds **20–36**.

Epigallocatechin gallate (**20**, Figure 5) is the most abundant catechin ester in green tea, chemically described as the epigallocatechin ester of gallic acid. Compound **20** is under investigation for its potential health benefits, and research suggests that it may have anti‐inflammatory, anticancer, cardioprotective, and neuroprotective properties [265, 266, 267, 268]. In one of Zhong et al.'s studies, the mechanism behind the antiviral effect of treatment with **20** on HBV‐expressing HepG2 and HepG2.2.15 cells was examined. They observed that HBV induced incomplete autophagy in hepatoma cells thereby promoting viral replication, while **20** increased autophagosome formation and lysosomal acidification, thus inducing a complete autophagic process. Similarly to starvation‐induced autophagy, it resulted in an unfavorable outcome for HBV replication. Evidence also showed that **20** increased p62 degradation and GFP‐LC3 processing in HBV‐transfected cells [158]. This finding aligns with previously reported data, which revealed that **20** stimulates autophagy through the upregulation of the AMPK signaling pathway in primary mouse hepatocytes [269].

#### 3.6.2 Deoxyshikonin

Deoxyshikonin (**21**, Figure 5) is an active ingredient isolated from *Lithospermum erythrorhizon* in 1988 for its antidermatophytic properties [270]. Since then, **21** demonstrated a diverse array of biological effects, showcasing its efficacy as an anticancer, anti‐inflammatory, antibacterial, and antiviral agent. Recently, a study investigated the effect and underlying mechanisms of **21** on RV replication, marking the first exploration of its role in this context. The compound was found to repress RV replication, concurrently mitigating RV‐induced autophagy and oxidative stress. Further examination of **21**'s influence on RV‐WA‐infected Caco‐2 cells revealed a reduction in total intracellular viral RNA levels and RV antigen levels. Elevated levels of autophagy‐related proteins induced by RV infection were countered by **21**, supporting its role in mitigating autophagy. Additionally, **21** was shown to reduce ROS levels and enhance glutathione levels, thereby repressing oxidative stress in RV‐infected cells. The investigation extended to in vivo assessments, in which **21** exhibited a protective function against RV‐induced mortality, diarrhea, and body weight loss in suckling mice. Reduced RV antigen levels, LC3‐II/LC3‐I ratio, and oxidative stress markers further endorsed **21**'s effectiveness in reducing RV replication in vivo. Evidence supported the pivotal role of sirtuin 1 (SIRT1)/forkhead box protein O1 (FOXO1)/Rab7 pathway modulation in **21**‐mediated reduction of RV replication, as indicated by the reversal of effects through SIRT1 overexpression [159].

#### 3.6.3 Gallic Acid

Gallic acid (**22**, Figure 5) is an antioxidant widely abundant in plants, belonging to the family of phenolic acids. It is the main component of the previously discussed gallotannins. The pleiotropic nature of this compound propelled research in different fields of drug discovery. Investigations have ranged from the development of semisynthetic derivatives to exploring its potential for treating or protecting against cancer, inflammatory‐based diseases, and infections [271]. Regarding the latter, **22** has recently been found to restore cellular viability in H1N1 influenza virus‐infected A549 cells, without cytotoxic effects. It also effectively reduced the viral activity of the H1N1 influenza virus, leading to a decrease in the production of key viral proteins (M1, M2, and NP). The study further revealed a significant reduction in autophagic activity, as evidenced by decreased LC3B‐II conversion and LC3B‐II/LC3B‐I ratio. The acridine orange staining assay confirmed a diminished accumulation of autophagosomes in H1N1 influenza virus‐infected cells, emphasizing the ability of **22** to modulate autophagy with antiviral effects [160].

#### 3.6.4 Urolithin A

Ellagitannins comprise a broad category of hydrolyzable tannins, commonly found in many dietary plants. Upon hydrolysis, ellagic acid is released, and within the colon, it is metabolized by gut microbiota, forming dibenzopyranone species, known as urolithins [272]. To date, 13 urolithins, along with their conjugated metabolites, have been described and found in different biological fluids. Recent studies have highlighted urolithins as the primary active form of ellagic acid in vivo. These compounds have garnered attention as interesting pleiotropic agents, mainly exerting anti‐inflammatory, antioxidant, anticancer, and neuro‐ and cardioprotective effects [273]. A 2022 study by Wang et al. revealed novel insights into the antiviral capabilities of urolithins A, B, and C, with urolithin A (**23**, Figure 5) standing out as a potent antiviral agent with low toxicity. The authors investigated the antiviral potential of **23** against EV71 infection. Results demonstrated a significant, dose‐dependent antiviral effect of **23**, effectively inhibiting proliferation and surpassing the efficacy of ribavirin in SK‐N‐SH cells. While **23** proved effective in treating EV71, it exerted neither prophylactic nor viricidal effects. The research also explored the influence of **23** on autophagy and apoptosis in EV71‐infected cells. Compound **23** induced both autophagy and apoptosis, as highlighted by the increased LC3‐II and cleaved‐caspase‐3 levels, contributing to viral clearance. However, **23** did not exhibit any antiviral effect against the influenza virus [161]. Further investigations are needed to understand compound **23**'s impact on the proliferation of other viruses.

### 3.7 Phenols

#### 3.7.1 Eugenol

Eugenol (**24**, Figure 5) is a volatile phenolic monoterpenoid found in spices like pepper, cinnamon, and nutmeg, but mainly clove (*Eugenia caryophyllata* or *Syzygium aromaticum*). Traditionally employed for its antimicrobial and analgesic properties, it has recently been studied for its anticancer and antiviral potential. As the result of a drug screening to identify ethnomedicines inhibiting the dissociation of Beclin1 from BCL2, clove extract emerged as the best‐performing. The effect was observed in uninfected and an IAV‐infected A549 cell line and in the latter, it could counter IAV‐triggered autophagy. In addition, it was demonstrated to inhibit IAV replication and prevent cell death in infected cells. The authors hypothesized that **24** inhibited the expression of autophagic genes by reducing oxidative stress and inhibiting different signaling pathways, including autophagy and ERK, and p38MAPK and IKK/NF‐κB cascades [162]. In combination with spermidine, **24** significantly increased autophagy in several cell lines. It was speculated that a supplement containing both bioactives could present a novel preventive treatment for SARS‐CoV‐2 infections, owing to the known association between chronic inflammation and more severe outcomes in COVID‐19 patients, and the role of defective autophagy in chronic inflammation [274]. Nonetheless, the precise antiviral efficacy of this combination is still unclear and requires further research.

### 3.8 Phenolic Acids

#### 3.8.1 Lithospermic Acid

Lithospermic acid (**25**, Figure 5) is an enantiomerically pure polyphenol extracted from *Lithospermum ruderale* and also found in other medicinal plants like *Salvia miltiorrhiza*, a traditional Chinese medicine used for treating diabetes, vascular diseases, and hepatitis B [275]. Building on this, the evaluation of **25**‐triggered effects on HBV infection was conducted. Compound **25** exhibited anti‐HBV activity in vitro and in vivo, in HepG2.2.15 cells and HBV‐HDI C57BL/6 mice. On a molecular level, **25** could improve lysosomal acidification and maturation, thereby reinstating the autophagic process impeded by HBV. Indeed, it is known that HBV hijacks the host's autophagic machinery by impairing lysosomal maturation, thereby blocking autophagic degradation. Lysosomal fluorescence staining assays and the reduction of p62 levels confirmed **25**‐induced autophagy activation in vivo. Furthermore, **25** was found to increase LAMP1 and LAMP2 expression and also influence the PI3K/AKT/mTOR signaling pathway—by impeding mTOR and AKT phosphorylation in mice but had no significant effects on p‐JNK, ATG7, and Beclin‐1 [163]. Future advancements in HBV treatment could rely on the combination of traditional antivirals with innovative agents that operate through distinct mechanisms, including autophagy‐modulating agents.

#### 3.8.2 Rhein (Cassic Acid)

Traditionally employed as a laxative, the rhubarb plant contains various anthraquinones that contribute to this therapeutic effect. Among them, rhein (**26**, Figure 5) was first isolated in 1895. It commonly occurs as rhein‐8‐glucoside in rhubarb species like *Rheum undulatum* and *Rheum palmatum*, but also in *Cassia reticulata*. In several ethnomedicines, it has been exploited for its antioxidant, anti‐inflammatory, and hepatoprotective effects [276]. In the framework of antiviral research, a study investigated the in vitro and in vivo anti‐IAV effects and mechanisms of action of **26**. Employing various assays, including plaque inhibition, qRT‐PCR, ELISA, and Western blot analysis, the research revealed that **26** significantly inhibits IAV adsorption and replication. It mitigated IAV‐induced oxidative stress, suppressing TLR4, Akt, p38, JNK‐MAPK, and NF‐κB pathways and reducing the production of inflammatory cytokines and matrix metalloproteinases in vitro. In vivo testing in mice confirmed compound **26**'s ability to enhance survival rates, attenuate pulmonary cytokines, and alleviate histopathological changes [164]. While the ability of **26** to regulate autophagy in human cells is well‐established [277], its impact on viral infections requires additional research. Future endeavors into this matter might uncover novel insights that could contribute to the development of innovative therapeutic interventions for viral diseases.

#### 3.8.3 Caffeic Acid

Caffeic acid (**27**, Figure 5) is a widely distributed polyphenol, identified in several plant sources. Compound **27** displays a plethora of biological functions, with its role as an antioxidant being particularly outstanding [278]. Exploiting this feature, **27** was explored as a potential antiviral agent against HCV, inducing oxidative stress and fitting the framework of applying antioxidants as a treatment for HCV. It emerged that **27** effectively inhibited HCV replication through multiple mechanisms of action. Compound **27** induced the expression of heme oxygenase‐1 (HO‐1), leading to the activation of the interferon alpha (IFNα) antiviral response and upregulated Nrf2. When Nrf2 was silenced, HO‐1 expression decreased, and HCV expression was restored. Further insights revealed that **27** reduced kelch‐like ECH‐associated protein 1 (Keap1) expression through p62/sequestosome1 (p62)‐mediated autophagy, as confirmed by the induction of LC3‐II. Hence, the study established the crucial role of p62‐mediated autophagy in **27**‐induced Keap1 downregulation. This ultimately modulated the Keap1/Nrf2 interaction, inducing HO‐1 and triggering an IFNα‐mediated antiviral response [165].

#### 3.8.4 Mycophenolic Acid

Mycophenolic acid (**28**, Figure 5) was isolated from *Penicillum brevicompactum* at the end of the 19th century. After the discovery of its antibacterial properties, it also proved to have antiviral, antifungal, and anticancer effects. In our days, it is used as an immunosuppressant medication to prevent organ transplantation rejection and treat autoimmune diseases. Its effects are linked to its potent and reversible inhibitory effect on inosine‐5′‐monophosphate dehydrogenase (IMPDH), hampering purine synthesis [279]. Compound **28** emerged as an interesting anti‐HCV agent, with the acknowledged antiviral mechanisms being guanosine depletion and IFN‐stimulated genes' upregulation [280, 281]. However, the combination of these mechanisms could not fully account for the observed inhibitory effect on HCV replication. Fang et al. went on to perform studies aimed at fully elucidating the mechanism underlying the anti‐HCV activity of **28** in Huh7 cells. Treatment with **28** demonstrated a suppressive effect on autophagy in Huh7 cells, evidenced by reduced levels of LC3B‐II, diminished autophagosome formation, and increased p62 levels compared to untreated cells. **28** not only inhibited constitutive autophagy but also attenuated **6**‐ or HCV‐induced autophagy and downregulated the expression of key ATGs (ATG3, ATG5, and ATG7). In the presence of bafilomycin A1, **28**‐treated cells exhibited decreased LC3B‐II levels and increased p62 levels, indicating a direct impact on autophagy suppression but not a link to enhanced autophagosome clearance. Overall, treatment with **28** significantly reduced HCV replication in a dose‐dependent manner, as evidenced by inhibited HCV RNA expression and diminished HCV core protein levels [166]. Another study focused on MPA's impact on DENV replication in stable BHK‐21/DENV2 replicon cells. Similarly to its effects on HCV‐infected Huh7 cells, **28** exhibited significant inhibitory effects on DENV2 subgenomic RNA replication. This was associated with altered autophagic marker levels, as indicated by decreased p62 and LC3‐II protein levels and upregulation of ATG4A, involved in LC3‐II delipidation. The suppression of ATG proteins associated with pre‐autophagosomal structure (PAS) formation and the enhancement of purine nucleotide salvage pathways were also observed [167]. These findings parallel those previously reported, suggesting a common mechanism of **28**'s antiviral action through autophagy modulation. The consistent reduction in viral titers across both studies underscores **28**'s potential as a broad‐spectrum antiviral agent and contributes to a deeper understanding of its role in host–virus interactions.

### 3.9 Alkaloids

#### 3.9.1 Berberine

Berberine (**29**, Figure 5) is a naturally occurring benzylisoquinoline alkaloid, present as a quaternary ammonium salt in several medicinal plants' roots and rhizomes. It historically derived its name from its extraction sources, primarily *Hydrastis canadensis* (goldenseal) and *Berberis vulgaris* (barberry). **29** showcases a plethora of beneficial effects, from its well‐known anti‐dysenteric, antiobesity, and anti‐hyperlipidemic properties to its anti‐inflammatory and antiproliferative activity [282]. **29** displays an ambivalent nature in modulating autophagy since it can both stimulate or inhibit the process. Numerous studies have investigated the role of **29** in modulating autophagy, uncovering its role as an inducer in mature adipocytes, and suggesting its application as a novel approach to prevent obesity. In the context of acute lymphoblastic leukemia, **29** exerts its influence by inactivating the AKT/mTORC1 signaling pathway, thereby triggering autophagic cell death and attenuating cancer progression [283]. In glioblastoma, **29** was able to reduce temozolomide resistance by inducing autophagy via activation of the ERK1/2 signaling pathway [284]. Significantly, **29**'s ability to induce autophagy leading to therapeutic effects might extend to the treatment of conditions like atherosclerosis and hepatic steatosis, as well as the prevention of pulmonary fibrosis. Conversely, **29**‐dependent autophagy inhibition was highlighted in myocardial hypoxia and ischemia‐reperfusion injury. One intriguing potential application involves combining **29**, which demonstrates dose‐dependent autophagy inhibition, with cisplatin in anticancer therapies. This synergistic approach mitigates the adverse effects of cisplatin‐induced kidney damage [285]. In recent years, the antiviral activity of **29** has been reported against herpes, influenza, and respiratory syncytial viruses. The main hypothesis regarding the mode of action of **29** in herpes simplex infection relies on its ability to modulate the NF‐κB pathway, thereby impeding viral proliferation [168]. In this context, the correlation between **29** and autophagy has been elucidated in EV71 infection. The findings suggested that **29** caused a dose‐dependent reduction of RNA and protein synthesis by downregulating the MEK/ERK signaling pathway and suppressed EV71‐induced autophagy by both AKT protein activation and JNK and PI3K‐III phosphorylation inhibition [101].

#### 3.9.2 Berbamine

Berbamine (**30**, Figure 5) is another bioactive compound found in *Berberis* spp., endowed with anticancer, immunomodulatory, and cardiovascular properties [286, 287, 288]. Traditionally used in Chinese medicine to enhance hematopoiesis and bolster the immune function of individuals undergoing cancer treatment, its primary effects can be ascribed to its dual nature as an apoptotic and autophagic inducer, alongside its inhibitory effect on calcium channels [289, 290]. The first investigations into its antiviral activities focused on bovine viral diarrhea virus (BVDV) infection. Results uncovered that **30** acted as a late‐stage autophagy inhibitor by impeding the binding of LC3 to LAMP1, thus suppressing viral replication. Moreover, a tight correlation between autophagy and BVDV spread was revealed, as treatment with rapamycin increased viral replication [170]. Further studies exploited **30** as a host‐directed therapy to limit intestinal SARS‐CoV‐2 dissemination. A significantly decreased acquisition of SARS‐CoV‐2 and its Omicron BA.2 and BA.5 variants by human intestinal epithelial cells was reported, and investigations confirmed the BCL2‐interacting protein 3 (BNIP3)‐mediated autophagy mechanism [171].

#### 3.9.3 Evodiamine

The quinazolinocarboline alkaloid evodiamine (**31**, Figure 5) was isolated from the dried fruit of *Evodia rutaecarpa*. It is acknowledged as a topoisomerase I inhibitor, capable of inducing apoptosis in different tumor cells [291]. Expanding upon their initial investigation on the anti‐IAV activity of procyanidin, Dai et al. introduced a follow‐up study that examined **31** in the same experimental settings, advancing our understanding of how natural compounds modulate autophagy and their impact on viral infections. Aiming to identify extracts able to inhibit the formation of the ATG5‐ATG12‐ATG16 complex, they performed a fluorescence complementation‐fluorescence resonance energy transfer (BiFC‐FRET) screening on 83 medicinal plants and selected *E. rutaecarpa* extract's main active ingredient **31** to evaluate its anti‐IAV activity in MDCK cells. The study demonstrated the inhibition of IAV replication by plaque inhibition assay. In the framework of autophagy modulation, **31** hampered the accumulation of LC3‐II and p62 and EFGP‐LC3 aggregation. Following treatment with **31**, lowered levels of autophagy‐associated genes (i.e., ATG5, ATG7, and ATG12) were observed, coupled with a reduction of IAV‐induced cytokine release. Remarkably, **31** demonstrated the ability to thwart increased AMPK and tuberous sclerosis complex 2 (TSC2) activities and downregulation of mTOR, which were prompted by IAV infection [172].

### 3.10 Biogenic Amines

#### 3.10.1 Spermidine and Spermine

Ubiquitous in ribosomes and tissues, spermidine is a pleiotropic aliphatic polyamine, a precursor to spermine and thermospermine. Spermidine (**32**, Figure 5) can promote longevity and delay or suppress the occurrence and severity of age‐related diseases. It can promote autophagy through inhibition of Raf phosphorylation, modulating the MAPK signaling pathway [292]. This ability to induce autophagy has been investigated in the context of neurodegenerative diseases, but its antiviral potential has been poorly examined thus far. A study on SARS‐Cov‐2‐mediated autophagy dysregulation highlighted that treatment with 100 μM **32** and spermine (**33**, Figure 5) inhibited the production of viral particles by 87% and 54% in VeroFM cells, respectively. SARS‐Cov‐2 is known to hijack the host cell by blocking the autophagic flux and reducing endogenous polyamine levels to ensure effective propagation. Research results indicated that **32** and **33** supplementation could restore autophagy in primary human lung cells and intestinal organoids, limiting SARS‐CoV‐2 replication [173].

#### 3.10.2 Melatonin

In vertebrates, melatonin (**34**, Figure 5) is released by the pineal gland in response to darkness. This neurohormone exerts its endogenous function by regulating the sleep–wake pattern, but it was found to also possess antioxidant and anti‐inflammatory properties. Frequently marketed as a supplement to address sleep‐related issues, **34** has recently garnered attention for its anticancer and antiviral potential. In a rabbit model of acute liver failure induced by the rabbit hemorrhagic disease virus (RHDV), **34** demonstrated protective effects. RHDV infection is known to induce a robust autophagic response, including increased expression of key autophagy markers (i.e. Beclin‐1, LC3‐II/LCI ratio, and ATG5‐ATG12‐ATG16 trimer complex), along with the formation of autophagosomes and autophagolysosomes. Administration of **34** inhibited this autophagic cascade, resulting in a lower late‐stage autophagic activity and an increased apoptosis. Mechanistically, **34**‐induced modulation of autophagy is interconnected with its attenuation of oxidative stress and ER stress. Importantly, **34**'s impact extended to inhibiting RHDV RNA replication, suggesting a link between autophagy suppression, ER stress, oxidative stress, and overall viral replication reduction [174]. More recently, a study examined compound **34**'s effects in JEV‐infected SK‐N‐SH neuroblastoma cells. Also in this experimental condition, autophagy modulation played a pivotal role. JEV triggered autophagy while concurrently impeding its normal functioning, as emphasized by the heightened SQSTM1/p62 levels. Treatment with **34** thwarted this autophagy impairment. Furthermore, JEV infection activated the serine/threonine protein phosphatase calcineurin (CaN), contributing to neuroinflammatory responses and neurotoxicity. Compound **34** inhibited CaN activity, thereby alleviating JEV‐induced neuroinflammation and autophagy. While this study unveiled **34**'s association with CaN alteration and autophagy in the context of JEV‐mediated neurotoxicity, further research is imperative to unravel the intricate mechanisms governing these interactions in vitro and/or in vivo [175].

### 3.11 Macrocycles

#### 3.11.1 Bafilomycin A1

Bafilomycin A1 (**35**, Figure 5) belongs to a family of macrolide antibiotics produced by several *Streptomycetes* strains. They are characterized by a 16‐membered lactone scaffold. Bafilomycins exhibit a diverse array of biological activities, encompassing anticancer, antiparasitic, and immunosuppressive effects [293, 294]. In particular, **35** specifically binds to the outer surface of the V0 domain of vacuolar‐type H^+^‐ATPase, a key proton pump that actively acidifies lysosomes and vacuoles, regulating pH levels to support essential cellular processes like degradation and recycling. This natural compound can be considered a late‐stage autophagy inhibitor since it hinders the fusion of the autophagosome with the lysosome. Its interaction with the V‐ATPase forbids lysosome acidification and lysosomal protease activation [293, 295]. While **35** is often employed in cell biological assays, its clinical use is limited by a poor toxicity profile [296]. Considering the vital role of lysosome acidification in the life cycle of several viruses, the antiviral activity of **35** has been evaluated in different infection conditions. Regarding ZIKV infections in Vero cells, fluorescence microscopy experiments revealed that the modulation of autophagy in A549 cells exploiting **35** exhibited no discernible impact on the quantity of ZIKV‐positive cells, and the following observed antiviral effects were not primarily ascribed to its influence on autophagy. The findings suggest a departure from studies associating direct modulation of autophagy with the ZIKV life cycle, with noted variations in experimental parameters, such as incubation periods and concentrations [176]. Various viruses, including EBV, interact with host autophagy to enhance their survival. EBV, a γ‐herpes virus linked to epithelial and lymphoid malignancies, establishes latent infections and periodically reactivates. In Burkitt's lymphoma cells, an autophagy increase during early EBV lytic activation was highlighted. In cells with late antigen defects, EBV suppresses autophagy as lytic protein levels rise. **35**‐mediated autophagy inhibition boosted EBV lytic gene expression, intracellular viral DNA, and progeny yield, suggesting the potential use of autophagy inhibitors for oncolytic viral therapy in EBV‐related lymphomas [177]. The potential of **35** was also investigated as a treatment option in the fight against SARS‐CoV‐2 and its variant strains. Results showed that **35**, at a concentration of 500 nM, effectively inhibited viral RNA synthesis of SARS‐CoV‐2 and its Beta and Delta variants. Utilizing a human lung xenograft mouse model, **35** demonstrated significant suppression of SARS‐CoV‐2 replication in lung tissues. Histopathological examination revealed **35**'s ability to alleviate viral‐induced inflammatory responses and cell damage. Even though its role in autophagy modulation was not reconfirmed in the specific experimental setting, immunohistochemistry analysis further supported the reduction of inflammatory exudation and infiltration in SARS‐CoV‐2‐infected human lung xenografts, suggesting that **35** may hold promise as a potential candidate drug for SARS‐CoV‐2 treatment [178].

#### 3.11.2 Rapamycin

Rapamycin (**36**, Figure 5) was isolated in 1972 from *Streptomyces hygroscopicus*. It is a potent macrolide autophagy inducer endowed with immunosuppressant properties due to its inhibitory effects on mTOR [297, 298]. While it is often utilized for the prevention of organ transplant rejection as a prophylactic treatment [299], recent studies have investigated its potential antiviral properties, which are intricately associated with its ability to modulate the autophagic processes. Guo et al. elucidated the relationship between autophagy and the transmissible gastroenteritis virus (TGEV) replication employing **36**. TGEV infection was found to activate autophagy in host cells, leading to an increase in autophagosome‐like vesicles. The replication of TGEV was dependent on this autophagic response, as indicated by elevated levels of the autophagosome marker protein LC3‐II. Inhibiting autophagy enhanced TGEV replication while activating autophagy using **36** reduced viral replication [179]. Critical insights into ZIKV infection pathogenesis were revealed by infecting human fetal neural stem cells (fNSCs). It was found that NS4A and NS4B ZIKV proteins inhibited the Akt‐mTOR pathway, disrupting neurogenesis and inducing autophagy. In this context, the induction of autophagy by **36** promoted ZIKV load in both fNSCs and HeLa cells [57]. The discovery of **36**'s immunosuppressive properties catalyzed the subsequent development of numerous derivatives, to be used as a therapy for transplant rejection. The first‐generation rapalogs include everolimus, ridaforolimus, umirolimus, and the pro‐drug temsirolimus, binding to FKBP‐rapamycin‐binding (FRB) domain. However, limitations such as incomplete target inhibition and side effects prompted the development of second‐generation rapalogs, designed to enhance specificity and efficacy. These newer compounds target the kinase domain, acting as ATP competitors, but their autophagy modulation effects within host‐targeting antiviral research remain to be investigated.

## 4 Challenges and Opportunities in Using Natural Products as Autophagy Modulators

Natural compounds display high complexity and variability and tend to defy Lipinski's rule of five, displaying higher molecular masses, hydrophilicity, and molecular rigidity, and a higher ratio of sp^3^ carbon and oxygen atoms to nitrogen and halogen atoms [300]. These features can be favorable in drug discovery, uncovering unexplored scaffolds and synthetically challenging chemical entities that have evolved to perform precise biological activities with eventually unique mechanisms of action, thus designating the exploration of natural products as an everlasting invaluable pursuit. Nonetheless, their clinical application is prevented by considerable drawbacks such as cytotoxicity, poor pharmacokinetics, and, more commonly, nonspecific side effects [301] as for wortmannin (**1**), tunicamycin (**6**), bafilomycin A1 (**35**), and rapamycin (**36**), which currently represent benchmark references in autophagy assays, together with other synthetic compounds such as 3‐methyladenine and chloroquine [301, 302]. Furthermore, while some natural products have been identified as autophagy modulators, there is often a lack of rigorous in vitro and in vivo studies to confirm their inhibitory mechanism, not to mention their efficacy and safety. Finally, given the broad biological activity usually presented by natural compounds and mixtures, it is important to consider the multiple interactions with various cellular pathways and the potential multi‐target biological effects uncovering the mechanism of action of said compounds.

## 5 Conclusion and Future Perspectives

Autophagy plays a crucial, yet controversial, role in the context of viral infections, as it can be exploited by the host as a first‐line defense mechanism but also by external pathogens as support for their replication and evasion from the host's innate response. The machinery is finely tuned through cascades and can be regulated by multiple modulators, rendering its exploitation a strategic antiviral weapon. As also confirmed by the ample use of this strategy against many pathologies that range from tumors to degenerative diseases and viral infections, in particular, considering the aftermath of recent pandemics such as the COVID‐19 outbreak and the persistent challenges posed by endemic infections, the pursuit of discovering new antiviral drugs is impelling. The lessons learned from the global response to these health crises emphasize the need for versatile and effective antiviral treatments to safeguard public health. In the context of antiviral drug discovery, host‐targeting antivirals represent a promising alternative, offering versatility and potential for broad‐spectrum activity as they may be less susceptible to drug resistance onset compared to traditional virus‐targeting antivirals. In recent years, autophagy modulation has emerged as a promising host‐targeting antiviral strategy for pathogen elimination, but its full potential has not yet been thoroughly explored. Moreover, the exploration of natural products as potent modulators presents a promising avenue for the development of novel therapeutic host‐targeting interventions against viral infections, as these natural sources have already been abundantly employed in the context of cancer treatment. This review aims to bridge this gap by presenting the state‐of‐the‐art of natural autophagy modulators in the context of viral infections. Considering the therapeutic potential of autophagy modulation in virally infected cells and leveraging the diverse array of natural autophagy modulators, this study conceives both the prospects of developing new and optimized derivatives and the repurposing of known autophagy modulators for testing in viral infections. Nonetheless, several aspects warrant further investigation; primarily, there is a need for a more comprehensive knowledge of the mechanisms by which some phytocompounds or extracts exert their autophagy modulation; understanding these mechanisms could reveal new potential targets for therapeutic strategies. Additionally, addressing challenges as off‐target effects and toxicity is pivotal for the development of synthetic and semisynthetic new compounds. Finally, the exploration of synergistic effects of combined autophagy modulators can pave the way for more effective strategies in the context of drug resistance.

## Data Availability

Data sharing is not applicable to this article as no datasets were generated or analyzed during the current study.

## References

[1] N. N. Noda and F. Inagaki , “Mechanisms of Autophagy,” Annual Review of Biophysics 44, no. 2015 (2015): 101–122, 10.1146/ANNUREV-BIOPHYS-060414-034248/CITE/REFWORKS. 25747593

[2] G. Mariño and C. López‐Otín , “Autophagy: Molecular Mechanisms, Physiological Functions and Relevance in Human Pathology,” Cellular and Molecular Life Sciences CMLS 61, no. 12 (2004): 1439–1454, 10.1007/s00018-004-4012-4. 15197469 PMC7079832

[3] V. Giansanti , A. Torriglia , and A. I. Scovassi , “Conversation Between Apoptosis and Autophagy: Is It Your Turn or Mine?,” Apoptosis 16, no. 4 (2011): 321–333, 10.1007/S10495-011-0589-X/FIGURES/4. 21404107

[4] T. Chen , S. Tu , L. Ding , M. Jin , H. Chen , and H. Zhou , “The Role of Autophagy in Viral Infections,” Journal of Biomedical Science 30 (2023): 5, 10.1186/s12929-023-00899-2. 36653801 PMC9846652

[5] Y. Liu , T. Zhou , J. Hu , et al., “Targeting Selective Autophagy as a Therapeutic Strategy for Viral Infectious Diseases,” Frontiers in Microbiology 13 (2022): 889835, 10.3389/fmicb.2022.889835. 35572624 PMC9096610

[6] M. A. A. Al‐Bari , Y. Ito , S. Ahmed , N. Radwan , H. S. Ahmed , and N. Eid , “Targeting Autophagy With Natural Products as a Potential Therapeutic Approach for Cancer.” International Journal of Molecular Sciences (MDPI, September 1, 2021), 10.3390/ijms22189807. PMC8467030 34575981

[7] S. Mushtaq , B. H. Abbasi , B. Uzair , and R. Abbasi , “Natural Products as Reservoirs of Novel Therapeutic Agents,” EXCLI Journal 17 (2018): 420–451, 10.17179/EXCLI2018-1174. 29805348 PMC5962900

[8] R. A. González‐polo , E. Pizarro‐estrella , S. M. S. Yakhine‐diop , et al., “Autophagy Networks in Inflammation,” in The Basics of Autophagy (2016), 3–20, 10.1007/978-3-319-30079-5_1.

[9] W. Wu , X. Luo , and M. Ren , “Clearance or Hijack: Universal Interplay Mechanisms Between Viruses and Host Autophagy From Plants to Animals,” Frontiers in Cellular and Infection Microbiology 11 (2022): 786348, 10.3389/FCIMB.2021.786348/BIBTEX. 35047417 PMC8761674

[10] W. Ye , C. Fan , K. Fu , et al., “The SAR and Action Mechanisms of Autophagy Inhibitors That Eliminate Drug Resistance,” European Journal of Medicinal Chemistry 244, no. August (2022): 114846, 10.1016/j.ejmech.2022.114846. 36283182

[11] T. Chen , S. Tu , L. Ding , M. Jin , H. Chen , and H. Zhou , “The Role of Autophagy in Viral Infections,” Journal of Biomedical Science 30, no. 1 (2023): 5, 10.1186/s12929-023-00899-2. 36653801 PMC9846652

[12] “Focusing on Autophagy,” Nature Cell Biology 12, no. 9 (2010): 813, 10.1038/ncb0910-813. 20811352

[13] G. Campiani , T. Khan , C. Ulivieri , et al., “Design and Synthesis of Multifunctional Microtubule Targeting Agents Endowed With Dual Pro‐Apoptotic and Anti‐Autophagic Efficacy,” European Journal of Medicinal Chemistry 235 (2022): 114274, 10.1016/J.EJMECH.2022.114274. 35344902

[14] T. Khan , N. Relitti , M. Brindisi , et al., “Autophagy Modulators for the Treatment of Oral and Esophageal Squamous Cell Carcinomas,” Medicinal Research Reviews 40, no. 3 (2020): 1002–1060, 10.1002/med.21646. 31742748

[15] M. Brindisi , C. Ulivieri , G. Alfano , et al., “Structure‐Activity Relationships, Biological Evaluation and Structural Studies of Novel Pyrrolonaphthoxazepines as Antitumor Agents,” European Journal of Medicinal Chemistry 162 (2019): 290–320, 10.1016/j.ejmech.2018.11.004. 30448418

[16] Z. Yang , J. J. Goronzy , and C. M. Weyand , “Autophagy in Autoimmune Disease,” Journal of Molecular Medicine 93, no. 7 (2015): 707–717, 10.1007/S00109-015-1297-8. 26054920 PMC4486076

[17] H. Sundaramurthi , S. L. Roche , G. L. Grice , et al., “Selective Histone Deacetylase 6 Inhibitors Restore Cone Photoreceptor Vision or Outer Segment Morphology in Zebrafish and Mouse Models of Retinal Blindness,” Frontiers in Cell and Developmental Biology 8 (2020): 689, 10.3389/fcell.2020.00689. 32984302 PMC7479070

[18] T. Nie , L. Zhu , and Q. Yang , “The Classification and Basic Processes of Autophagy,” Advances in Experimental Medicine and Biology 1208 (2021): 3–16, 10.1007/978-981-16-2830-6_1. 34260018

[19] G. Zaffagnini and S. Martens , “Mechanisms of Selective Autophagy,” Journal of Molecular Biology 428, no. 9 (2016): 1714–1724, 10.1016/J.JMB.2016.02.004. 26876603 PMC4871809

[20] Y. Liu , T. Zhou , J. Hu , et al., “Targeting Selective Autophagy as a Therapeutic Strategy for Viral Infectious Diseases.” Frontiers in Microbiology (Frontiers Media S.A., April 28, 2022), 10.3389/fmicb.2022.889835. PMC9096610 35572624

[21] K. R. Parzych and D. J. Klionsky , “An Overview of Autophagy: Morphology, Mechanism, and Regulation,” Antioxidants & Redox Signaling 20, no. 3 (2014): 460–473, 10.1089/ars.2013.5371. 23725295 PMC3894687

[22] Y. Ohsumi , “Molecular Dissection of Autophagy: Two Ubiquitin‐Like Systems,” Nature Reviews Molecular Cell Biology 2, no. 3 (2001): 211–216, 10.1038/35056522. 11265251

[23] E. Turco , D. Fracchiolla , and S. Martens , “Recruitment and Activation of the ULK1/Atg1 Kinase Complex in Selective Autophagy,” Journal of Molecular Biology 432, no. 1 (2020): 123–134, 10.1016/J.JMB.2019.07.027. 31351898 PMC6971721

[24] J. H. Hurley and L. N. Young , “Mechanisms of Autophagy Initiation,” Annual Review of Biochemistry 86, no. 2017 (2017): 225–244, 10.1146/ANNUREV-BIOCHEM-061516-044820/CITE/REFWORKS. PMC5604869 28301741

[25] N. Mizushima , “The Role of the Atg1/ULK1 Complex in Autophagy Regulation,” Current Opinion in Cell Biology 22, no. 2 (2010): 132–139, 10.1016/J.CEB.2009.12.004. 20056399

[26] M. Zachari and I. G. Ganley , “The Mammalian ULK1 Complex and Autophagy Initiation,” Essays in Biochemistry 61, no. 6 (2017): 585–596, 10.1042/EBC20170021. 29233870 PMC5869855

[27] E. Itakura , C. Kishi , K. Inoue , and N. Mizushima , “Beclin 1 Forms Two Distinct Phosphatidylinositol 3‐Kinase Complexes With Mammalian Atg14 and UVRAG,” Molecular Biology of the Cell 19, no. 12 (2008): 5360–5372, 10.1091/MBC.E08-01-0080. 18843052 PMC2592660

[28] D. Siva Sankar and J. Dengjel , “Protein Complexes and Neighborhoods Driving Autophagy,” Autophagy 17, no. 10 (2021): 2689–2705, 10.1080/15548627.2020.1847461. 33183148 PMC8526019

[29] H. Suzuki , T. Osawa , Y. Fujioka , and N. N. Noda , “Structural Biology of the Core Autophagy Machinery,” Current Opinion in Structural Biology 43 (2017): 10–17, 10.1016/J.SBI.2016.09.010. 27723509

[30] N. Mizushima , A. Kuma , Y. Kobayashi , et al., “Mouse Apg16L, a Novel WD‐Repeat Protein, Targets to the Autophagic Isolation Membrane With the Apg12‐Apg5 Conjugate,” Journal of Cell Science 116, no. 9 (2003): 1679–1688, 10.1242/JCS.00381. 12665549

[31] I. Dikic and Z. Elazar , “Mechanism and Medical Implications of Mammalian Autophagy,” Nature Reviews Molecular Cell Biology 19, no. 6 (2018): 349–364, 10.1038/s41580-018-0003-4. 29618831

[32] A. Rozières , C. Viret , and M. Faure , “Autophagy in Measles Virus Infection,” Viruses 9, no. 12 (2017): 359, 10.3390/V9120359. 29186766 PMC5744134

[33] C. Richetta , I. P. Grégoire , P. Verlhac , et al., “Sustained Autophagy Contributes to Measles Virus Infectivity,” PLoS Pathogens 9, no. 9 (2013): e1003599, 10.1371/JOURNAL.PPAT.1003599. 24086130 PMC3784470

[34] Y. Jiang , Y. Qin , and M. Chen , “Host–Pathogen Interactions in Measles Virus Replication and Anti‐Viral Immunity,” Viruses 8, no. 11 (2016): 308, 10.3390/V8110308. 27854326 PMC5127022

[35] X. Fu , Y. Ming , C. Li , et al., “ *Siniperca chuatsi* Rhabdovirus (SCRV) Induces Autophagy via PI3K/Akt‐MTOR Pathway in CPB Cells,” Fish & Shellfish Immunology 102 (2020): 381–388, 10.1016/J.FSI.2020.04.064. 32360913 PMC7252040

[36] B. Ding , L. Zhang , Z. Li , et al., “The Matrix Protein of Human Parainfluenza Virus Type 3 Induces Mitophagy That Suppresses Interferon Responses,” Cell Host & Microbe 21, no. 4 (2017): 538–547.e4, 10.1016/j.chom.2017.03.004. 28407488

[37] Y. Han , C. Wang , K. Lu , et al., “Bovine Parainfluenza Type 3 Virus Induces Incomplete Autophagy to Promote Viral Replication by Activated Beclin1 In Vitro,” Veterinary Microbiology 290 (2024): 109972, 10.1016/J.VETMIC.2023.109972. 38183839

[38] Y. Zhang , S. Wu , J. Lv , et al., “Peste des Petits Ruminants Virus Exploits Cellular Autophagy Machinery for Replication,” Virology 437, no. 1 (2013): 28–38, 10.1016/J.VIROL.2012.12.011. 23318276

[39] B. Yang , Q. Xue , J. Guo , et al., “Autophagy Induction by the Pathogen Receptor NECTIN4 and Sustained Autophagy Contribute to Peste des Petits Ruminants Virus Infectivity,” Autophagy 16, no. 5 (2020): 842–861, 10.1080/15548627.2019.1643184. 31318632 PMC7144873

[40] S. Ren , Z. U. Rehman , M. Shi , et al., “Syncytia Generated by Hemagglutinin‐Neuraminidase and Fusion Proteins of Virulent Newcastle Disease Virus Induce Complete Autophagy by Activating AMPK‐mTORC1‐ULK1 Signaling,” Veterinary Microbiology 230 (2019): 283–290, 10.1016/J.VETMIC.2019.01.002. 30658866

[41] P. E. Joubert , G. Meiffren , I. P. Grégoire , et al., “Autophagy Induction by the Pathogen Receptor CD46,” Cell Host & Microbe 6, no. 4 (2009): 354–366, 10.1016/j.chom.2009.09.006. 19837375

[42] G. Meiffren , P. E. Joubert , I. P. Grégoire , P. Codogno , C. Rabourdin‐Combe , and M. Faure , “Pathogen Recognition by the Cell Surface Receptor CD46 Induces Autophagy,” Autophagy 6, no. 2 (2010): 299–300, 10.4161/AUTO.6.2.11132. 20087059

[43] B. Ding , G. Zhang , X. Yang , et al., “Phosphoprotein of Human Parainfluenza Virus Type 3 Blocks Autophagosome‐Lysosome Fusion to Increase Virus Production,” Cell Host & Microbe 15, no. 5 (2014): 564–577, 10.1016/j.chom.2014.04.004. 24832451

[44] M. Keshavarz , F. Solaymani‐Mohammadi , S. M. Miri , and A. Ghaemi , “Oncolytic Paramyxoviruses‐Induced Autophagy; A Prudent Weapon for Cancer Therapy,” Journal of Biomedical Science 26, no. 1 (2019): 48, 10.1186/S12929-019-0542-9. 31217023 PMC6585078

[45] J. H. Cheng , Y. J. Sun , F. Q. Zhang , et al., “Newcastle Disease Virus NP and P Proteins Induce Autophagy via the Endoplasmic Reticulum Stress‐Related Unfolded Protein Response,” Scientific Reports 6, no. 1 (2016): 24721, 10.1038/srep24721. 27097866 PMC4838823

[46] M. I. Rozilah , K. Yusoff , S. L. Chia , and S. Ismail , “Autophagy Inhibition Suppresses Newcastle Disease Virus‐Induced Cell Death by Inhibiting Viral Replication in Human Breast Cancer Cells,” Virology 590 (2024): 109957, 10.1016/J.VIROL.2023.109957. 38100982

[47] C. Dinkins , J. Arko‐Mensah , and V. Deretic , “Autophagy and HIV,” Seminars in Cell & Developmental Biology 21, no. 7 (2010): 712–718, 10.1016/J.SEMCDB.2010.04.004. 20403451 PMC3108047

[48] R. Nardacci , F. Ciccosanti , C. Marsella , G. Ippolito , M. Piacentini , and G. M. Fimia , “Role of Autophagy in HIV Infection and Pathogenesis,” Journal of Internal Medicine 281, no. 5 (2017): 422–432, 10.1111/JOIM.12596. 28139864

[49] M. L. Gougeon and M. Piacentini , “New Insights on the Role of Apoptosis and Autophagy in HIV Pathogenesis,” Apoptosis 14, no. 4 (2009): 501–508, 10.1007/S10495-009-0314-1/METRICS. 19199038

[50] G. R. Campbell and S. A. Spector , “Inhibition of Human Immunodeficiency Virus Type‐1 Through Autophagy,” Current Opinion in Microbiology 16, no. 3 (2013): 349–354, 10.1016/J.MIB.2013.05.006. 23747172 PMC3742638

[51] S. W. Tang , A. Ducroux , K. T. Jeang , and C. Neuveut , “Impact of Cellular Autophagy on Viruses: Insights From Hepatitis B Virus and Human Retroviruses,” Journal of Biomedical Science 19, no. 1 (2012): 92, 10.1186/1423-0127-19-92. 23110561 PMC3495035

[52] C. F. Daussy , B. Beaumelle , and L. Espert , “Autophagy Restricts HIV‐1 Infection,” Oncotarget 6, no. 25 (2015): 20752–20753, 10.18632/ONCOTARGET.5123. 26309074 PMC4673226

[53] G. R. Campbell , R. S. Bruckman , S. D. Herns , S. Joshi , D. L. Durden , and S. A. Spector , “Induction of Autophagy by PI3K/MTOR and PI3K/MTOR/BRD4 Inhibitors Suppresses HIV‐1 Replication,” Journal of Biological Chemistry 293, no. 16 (2018): 5808–5820, 10.1074/JBC.RA118.002353. 29475942 PMC5912471

[54] R. Cabrera‐Rodríguez , S. Pérez‐Yanes , J. Estévez‐Herrera , et al., “The Interplay of HIV and Autophagy in Early Infection,” Frontiers in Microbiology 12 (2021): 661446, 10.3389/FMICB.2021.661446/BIBTEX. 33995324 PMC8113651

[55] M. H. Song , Y. Sun , and X. B. Qiu , “Hijacking Autophagy for Infection by Flaviviruses,” Virus Research 347 (2024): 199422, 10.1016/J.VIRUSRES.2024.199422. 38901564 PMC11252935

[56] P. Y. Ke , “The Multifaceted Roles of Autophagy in Flavivirus‐Host Interactions,” International Journal of Molecular Sciences 19, no. 12 (2018): 3940, 10.3390/IJMS19123940. 30544615 PMC6321027

[57] Q. Liang , Z. Luo , J. Zeng , et al., “Zika Virus NS4A and NS4B Proteins Deregulate Akt‐MTOR Signaling in Human Fetal Neural Stem Cells to Inhibit Neurogenesis and Induce Autophagy,” Cell Stem Cell 19, no. 5 (2016): 663–671, 10.1016/j.stem.2016.07.019. 27524440 PMC5144538

[58] B. R. Sahoo , A. Pattnaik , A. S. Annamalai , R. Franco , and A. K. Pattnaik , “Mechanistic Target of Rapamycin Signaling Activation Antagonizes Autophagy to Facilitate Zika Virus Replication,” Journal of Virology 94, no. 22 (2020): e01575‐20, 10.1128/JVI.01575-20 . PMC7592218 32878890

[59] R. Vandergaast and B. L. Fredericksen , “West Nile Virus (WNV) Replication Is Independent of Autophagy in Mammalian Cells,” PLoS One 7, no. 9 (2012): e45800, 10.1371/JOURNAL.PONE.0045800. 23029249 PMC3448696

[60] E. Beatman , R. Oyer , K. D. Shives , et al., “West Nile Virus Growth Is Independent of Autophagy Activation,” Virology 433, no. 1 (2012): 262–272, 10.1016/J.VIROL.2012.08.016. 22939285 PMC3444629

[61] S. Kobayashi , S. Kawai , Y. Fukuda , et al., “Rab27a Promotes Degradation of West Nile Virus E Protein in the Lysosome,” iScience 27, no. 4 (2024): 109539, 10.1016/j.isci.2024.109539. 38715944 PMC11075059

[62] H. Huang , R. Kang , J. Wang , G. Luo , W. Yang , and Z. Zhao , “Hepatitis C Virus Inhibits AKT‐Tuberous Sclerosis Complex (TSC), the Mechanistic Target of Rapamycin (MTOR) Pathway, Through Endoplasmic Reticulum Stress to Induce Autophagy,” Autophagy 9, no. 2 (2013): 175–195, 10.4161/AUTO.22791. 23169238 PMC3552882

[63] Y. R. Lee , S. H. Kuo , C. Y. Lin , et al., “Dengue Virus‐Induced ER Stress Is Required for Autophagy Activation, Viral Replication, and Pathogenesis Both In Vitro and In Vivo,” Scientific Reports 8, no. 1 (2018): 489, 10.1038/s41598-017-18909-3. 29323257 PMC5765116

[64] J. Wang , R. Kang , H. Huang , et al., “Hepatitis C Virus Core Protein Activates Autophagy Through EIF2AK3 and ATF6 UPR Pathway‐Mediated MAP1LC3B and ATG12 Expression,” Autophagy 10, no. 5 (2014): 766–784, 10.4161/AUTO.27954. 24589849 PMC5119055

[65] G. C. Das and F. B. Hollinger , “GSK‐3β as a Potential Coordinator of Anabolic and Catabolic Pathways in Hepatitis C Virus Insulin Resistance,” Intervirology 67 (2024): 6–18, 10.1159/000535787. 38104537 PMC10794973

[66] L. Wang , Y. Tian , and J. J. Ou hsiung J., “HCV Induces the Expression of Rubicon and UVRAG to Temporally Regulate the Maturation of Autophagosomes and Viral Replication,” PLoS Pathogens 11, no. 3 (2015): e1004764, 10.1371/JOURNAL.PPAT.1004764. 25807108 PMC4373777

[67] S. Y. Wu , Y. L. Chen , Y. R. Lee , et al., “The Autophagosomes Containing Dengue Virus Proteins and Full‐Length Genomic RNA Are Infectious,” Viruses 13, no. 10 (2021): 2034, 10.3390/V13102034/S1. 34696464 PMC8540618

[68] N. S. Heaton and G. Randall , “Dengue Virus‐Induced Autophagy Regulates Lipid Metabolism,” Cell Host & Microbe 8, no. 5 (2010): 422–432, 10.1016/j.chom.2010.10.006. 21075353 PMC3026642

[69] J. E. McLean , A. Wudzinska , E. Datan , D. Quaglino , and Z. Zakeri , “Flavivirus NS4A‐Induced Autophagy Protects Cells Against Death and Enhances Virus Replication,” Journal of Biological Chemistry 286, no. 25 (2011): 22147–22159, 10.1074/jbc.M110.192500. 21511946 PMC3121359

[70] Y. Wu , T. Zhou , J. Hu , et al., “Autophagy Activation Induces P62‐Dependent Autophagic Degradation of Dengue Virus Capsid Protein During Infection,” Frontiers in Microbiology 13 (2022): 889693, 10.3389/FMICB.2022.889693/BIBTEX. 35865923 PMC9294600

[71] P. Metz , A. Chiramel , L. Chatel‐Chaix , et al., “Dengue Virus Inhibition of Autophagic Flux and Dependency of Viral Replication on Proteasomal Degradation of the Autophagy Receptor P62,” Journal of Virology 89, no. 15 (2015): 8026–8041, 10.1128/JVI.00787-15/ASSET/572CB9EE-2DD4-4266-9FCB-5060DF7A6527/ASSETS/GRAPHIC/ZJV9990906320008.JPEG. 26018155 PMC4505648

[72] M. Lussignol , C. Queval , M.‐F. Bernet‐Camard , et al., “The Herpes Simplex Virus 1 Us11 Protein Inhibits Autophagy Through Its Interaction With the Protein Kinase PKR,” Journal of Virology 87, no. 2 (2013): 859–871, 10.1128/JVI.01158-12/ASSET/84F3116A-64A0-427F-8116-9934AF4B2948/ASSETS/GRAPHIC/ZJV9990971280008.JPEG. 23115300 PMC3554085

[73] M. T. Vo and Y. B. Choi , “Herpesvirus Regulation of Selective Autophagy,” Viruses 13, no. 5 (2021): 820, 10.3390/V13050820. 34062931 PMC8147283

[74] M. Cirone , “EBV and KSHV Infection Dysregulates Autophagy to Optimize Viral Replication, Prevent Immune Recognition and Promote Tumorigenesis,” Viruses 10, no. 11 (2018): 599, 10.3390/V10110599. 30384495 PMC6266050

[75] R. M. Rubio and I. Mohr , “Inhibition of ULK1 and Beclin1 by an α‐Herpesvirus Akt‐Like Ser/Thr Kinase Limits Autophagy to Stimulate Virus Replication,” Proceedings of the National Academy of Sciences 116, no. 52 (2019): 26941–26950, 10.1073/PNAS.1915139116. PMC6936557 31843932

[76] S. Ning and L. Wang , “How Oncogenic Viruses Exploit P62‐Mediated Selective Autophagy for Cancer Development,” Annals of Immunology & Immunotherapy 3, no. 1 (2021): 134. 34632457 PMC8496745

[77] S. Ning and L. Wang , “The Multifunctional Protein P62 and Its Mechanistic Roles in Cancers,” Current Cancer Drug Targets 19, no. 6 (2019): 468–478, 10.2174/1568009618666181016164920. 30332964 PMC8052633

[78] Y. Mohamud , Y. C. Xue , H. Liu , et al., “The Papain‐Like Protease of Coronaviruses Cleaves ULK1 to Disrupt Host Autophagy,” Biochemical and Biophysical Research Communications 540 (2021): 75–82, 10.1016/J.BBRC.2020.12.091. 33450483 PMC7836930

[79] Z. Zhao , K. Lu , B. Mao , et al., “The Interplay Between Emerging Human Coronavirus Infections and Autophagy,” Emerging Microbes & Infections 10, no. 1 (2021): 196–205, 10.1080/22221751.2021.1872353. 33399028 PMC7872537

[80] H. Maier and P. Britton , “Involvement of Autophagy in Coronavirus Replication,” Viruses 4, no. 12 (2012): 3440–3451, 10.3390/V4123440. 23202545 PMC3528273

[81] K. Miller , M. E. McGrath , Z. Hu , et al., “Coronavirus Interactions With the Cellular Autophagy Machinery,” Autophagy 16, no. 12 (2020): 2131–2139, 10.1080/15548627.2020.1817280. 32964796 PMC7755319

[82] X. Chen , K. Wang , Y. Xing , et al., “Coronavirus Membrane‐Associated Papain‐Like Proteases Induce Autophagy Through Interacting With Beclin1 to Negatively Regulate Antiviral Innate Immunity,” Protein & Cell 5, no. 12 (2014): 912–927, 10.1007/S13238-014-0104-6. 25311841 PMC4259884

[83] E. M. Cottam , H. J. Maier , M. Manifava , et al., “Coronavirus Nsp6 Proteins Generate Autophagosomes From the Endoplasmic Reticulum via an Omegasome Intermediate,” Autophagy 7, no. 11 (2011): 1335–1347, 10.4161/AUTO.7.11.16642. 21799305 PMC3242798

[84] N. C. Gassen , D. Niemeyer , D. Muth , et al., “SKP2 Attenuates Autophagy Through Beclin1‐Ubiquitination and Its Inhibition Reduces MERS‐Coronavirus Infection,” Nature Communications 10, no. 1 (2019): 5770, 10.1038/s41467-019-13659-4. PMC6920372 31852899

[85] P. Hou , X. Wang , H. Wang , et al., “The ORF7a Protein of SARS‐CoV‐2 Initiates Autophagy and Limits Autophagosome‐Lysosome Fusion via Degradation of SNAP29 to Promote Virus Replication,” Autophagy 19, no. 2 (2023): 551–569, 10.1080/15548627.2022.2084686. 35670302 PMC9851267

[86] Y. Zhang , H. Sun , R. Pei , et al., “The SARS‐CoV‐2 Protein ORF3a Inhibits Fusion of Autophagosomes With Lysosomes,” Cell Discovery 7, no. 1 (2021): 31, 10.1038/s41421-021-00268-z. 33947832 PMC8096138

[87] D. Chen and H. Zhang , “Autophagy in Severe Acute Respiratory Syndrome Coronavirus 2 Infection,” Current Opinion in Physiology 29 (2022): 100596, 10.1016/J.COPHYS.2022.100596. 36187896 PMC9514017

[88] D. Chen , Q. Zheng , L. Sun , et al., “ORF3a of SARS‐CoV‐2 Promotes Lysosomal Exocytosis‐Mediated Viral Egress,” Developmental Cell 56, no. 23 (2021): 3250–3263.e5, 10.1016/j.devcel.2021.10.006. 34706264 PMC8502680

[89] V. Paunovic , L. Vucicevic , M. Misirkic Marjanovic , et al., “Autophagy Receptor P62 Regulates SARS‐CoV‐2‐Induced Inflammation in COVID‐19,” Cells 12, no. 9 (2023): 1282, 10.3390/CELLS12091282/S1. 37174682 PMC10177105

[90] P. Jiao , W. Fan , X. Ma , et al., “SARS‐CoV‐2 Nonstructural Protein 6 Triggers Endoplasmic Reticulum Stress‐Induced Autophagy to Degrade STING1,” Autophagy 19, no. 12 (2023): 3113–3131, 10.1080/15548627.2023.2238579. 37482689 PMC10621274

[91] W. Su , X. Yu , and C. Zhou , “SARS‐CoV‐2 ORF3a Induces Incomplete Autophagy via the Unfolded Protein Response,” Viruses 13, no. 12 (2021): 2467, 10.3390/V13122467/S1. 34960736 PMC8706696

[92] X. Hui , L. Zhang , L. Cao , et al., “SARS‐CoV‐2 Promote Autophagy to Suppress Type I Interferon Response,” Signal Transduction and Targeted Therapy 6 (2021): 180, 10.1038/s41392-021-00574-8. 33966045 PMC8105701

[93] M. P. Taylor and K. Kirkegaard , “Potential Subversion of Autophagosomal Pathway by Picornaviruses,” Autophagy 4, no. 3 (2008): 286–289, 10.4161/AUTO.5377. 18094610

[94] M. Mauthe , N. Dinesh Kumar , P. Verlhac , N. van de Beek , and F. Reggiori , “HSBP1 Is a Novel Interactor of FIP200 and ATG13 That Promotes Autophagy Initiation and Picornavirus Replication,” Frontiers in Cellular and Infection Microbiology 11 (2021): 745640, 10.3389/FCIMB.2021.745640/BIBTEX. 34869056 PMC8634480

[95] Y. Mohamud and H. Luo , “The Intertwined Life Cycles of Enterovirus and Autophagy,” Virulence 10, no. 1 (2019): 470–480, 10.1080/21505594.2018.1551010. 30475087 PMC6550542

[96] L. Huang and J. Yue , “The Interplay of Autophagy and Enterovirus,” Seminars in Cell & Developmental Biology 101 (2020): 12–19, 10.1016/J.SEMCDB.2019.08.001. 31563390 PMC7102577

[97] X. Xi , X. Zhang , B. Wang , et al., “The Interplays Between Autophagy and Apoptosis Induced by Enterovirus 71.” PLoS ONE (Public Library of Science, February 20, 2013), 10.1371/journal.pone.0056966. PMC3577684 23437282

[98] J. Lai , I. C. Sam , and Y. Chan , “The Autophagic Machinery in Enterovirus Infection,” Viruses 8, no. 2 (2016): 32, 10.3390/V8020032. 26828514 PMC4776187

[99] J. Y. Lin and H. I. Huang , “Autophagy Is Induced and Supports Virus Replication in Enterovirus A71‐Infected Human Primary Neuronal Cells,” Scientific Reports 10, no. 1 (2020): 15234, 10.1038/s41598-020-71970-3. 32943650 PMC7499237

[100] B. Wang , Y. Zhu , L. Liu , et al., “Enterovirus 71 Induces Autophagy in Mice via MTOR Inhibition and ERK Pathway Activation,” Life Sciences 271 (2021): 119188, 10.1016/J.LFS.2021.119188. 33581126

[101] H. Wang , K. Li , L. Ma , et al., “Berberine Inhibits Enterovirus 71 Replication by Downregulating the MEK/ERK Signaling Pathway and Autophagy,” Virology Journal 14, no. 1 (2017): 2, 10.1186/s12985-016-0674-4. 28081706 PMC5234143

[102] Q. Xiang , P. Wan , G. Yang , et al., “Beclin1 Binds to Enterovirus 71 3D Protein to Promote the Virus Replication,” Viruses 12 (2020): 756, 10.3390/v12070756. 32674313 PMC7411969

[103] L. Cao , X. Zhang , S. Yuan , K. Cheng , and X. Zhang , “Autophagy Induced by Enterovirus 71 Regulates the Production of IL‐6 Through the P38MAPK and ERK Signaling Pathways,” Microbial Pathogenesis 131 (2019): 120–127, 10.1016/J.MICPATH.2019.03.028. 30910719

[104] Y. Mohamud , J. Shi , H. Tang , et al., “Coxsackievirus Infection Induces a Non‐Canonical Autophagy Independent of the ULK and PI3K Complexes,” Scientific Reports 10, no. 1 (2020): 19068, 10.1038/s41598-020-76227-7. 33149253 PMC7642411

[105] G. Liu , M. Zhong , C. Guo , et al., “Autophagy Is Involved in Regulating Influenza A Virus RNA and Protein Synthesis Associated With Both Modulation of Hsp90 Induction and MTOR/P70S6K Signaling Pathway,” International Journal of Biochemistry & Cell Biology 72 (2016): 100–108, 10.1016/j.biocel.2016.01.012. 26794463

[106] A. Zhou , W. Zhang , X. Dong , M. Liu , H. Chen , and B. Tang , “The Battle for Autophagy Between Host and Influenza A Virus,” Virulence 13, no. 1 (2022): 46–59. 34967267 10.1080/21505594.2021.2014680 PMC9794007

[107] R. Zhang , X. Chi , S. Wang , B. Qi , X. Yu , and J. L. Chen , “The Regulation of Autophagy by Influenza A Virus,” BioMed Research International 2014, no. 1 (2014): 498083, 10.1155/2014/498083. 24779013 PMC3980786

[108] O. P. Zhirnov and H. D. Klenk , “Influenza A Virus Proteins NS1 and Hemagglutinin Along With M2 Are Involved in Stimulation of Autophagy in Infected Cells,” Journal of Virology 87, no. 24 (2013): 13107–13114, 10.1128/jvi.02148-13. 24027311 PMC3838240

[109] R. Beale , H. Wise , A. Stuart , B. J. Ravenhill , P. Digard , and F. Randow , “A LC3‐Interacting Motif in the Influenza A Virus M2 Protein Is Required to Subvert Autophagy and Maintain Virion Stability,” Cell Host & Microbe 15, no. 2 (2014): 239–247, 10.1016/j.chom.2014.01.006. 24528869 PMC3991421

[110] R. Wang , Y. Zhu , J. Zhao , et al., “Autophagy Promotes Replication of Influenza A Virus In Vitro,” Journal of Virology 93, no. 4 (2019): e01984–18, 10.1128/jvi.01984-18. 30541828 PMC6363991

[111] Y. Wang , P. Sharma , M. Jefferson , et al., “Non‐Canonical Autophagy Functions of ATG16L1 in Epithelial Cells Limit Lethal Infection by Influenza A Virus,” EMBO Journal 40, no. 6 (2021): e105543, 10.15252/embj.2020105543. 33586810 PMC7957399

[112] Z. Zhou , X. Jiang , D. Liu , et al., “Autophagy Is Involved in Influenza A Virus Replication,” Autophagy 5, no. 3 (2009): 321–328, 10.4161/auto.5.3.7406. 19066474

[113] J. Ma , Q. Sun , R. Mi , and H. Zhang , “Avian Influenza A Virus H5N1 Causes Autophagy‐Mediated Cell Death Through Suppression of MTOR Signaling,” Journal of Genetics and Genomics 38, no. 11 (2011): 533–537, 10.1016/J.JGG.2011.10.002. 22133684

[114] H. Zhu , L. Han , X. Shi , et al., “Baicalin Inhibits Autophagy Induced by Influenza A Virus H3N2,” Antiviral Research 113 (2015): 62–70, 10.1016/j.antiviral.2014.11.003. 25446340

[115] X. Wang , T. Zheng , L. Lin , et al., “Influenza A Virus Induces Autophagy by Its Hemagglutinin Binding to Cell Surface Heat Shock Protein 90AA1,” Frontiers in Microbiology 11 (2020): 566348, 10.3389/FMICB.2020.566348/BIBTEX. 33117314 PMC7575715

[116] Y. Tian , M. L. Wang , and J. Zhao , “Crosstalk Between Autophagy and Type I Interferon Responses in Innate Antiviral Immunity,” Viruses 11, no. 2 (2019): 132, 10.3390/V11020132. 30717138 PMC6409909

[117] C. Richetta and M. Faure , “Autophagy in Antiviral Innate Immunity,” Cellular Microbiology 15, no. 3 (2013): 368–376, 10.1111/CMI.12043. 23051682

[118] P. Y. Ke , “Autophagy and Antiviral Defense,” IUBMB Life 74, no. 4 (2022): 317–338, 10.1002/iub.2582. 34859938

[119] D. A. Dias , S. Urban , and U. Roessner , “A Historical Overview of Natural Products in Drug Discovery,” Metabolites 2, no. 2 (April 2012): 303–336, 10.3390/metabo2020303. 24957513 PMC3901206

[120] G. Carullo , F. Sciubba , P. Governa , et al., “Mantonico and Pecorello Grape Seed Extracts: Chemical Characterization and Evaluation of In Vitro Wound‐Healing and Anti‐Inflammatory Activities,” Pharmaceuticals 13, no. 5 (2020): 97, 10.3390/PH13050097. 32423026 PMC7281466

[121] G. Carullo , A. Ahmed , A. Trezza , et al., “A Multitarget Semi‐Synthetic Derivative of the Flavonoid Morin With Improved In Vitro Vasorelaxant Activity: Role of CaV1.2 and KCa1.1 Channels,” Biochemical Pharmacology 185 (2021): 114429, 10.1016/J.BCP.2021.114429. 33513341

[122] S. Mazzotta , P. Governa , V. Borgonetti , et al., “Pinocembrin and Its Linolenoyl Ester Derivative Induce Wound Healing Activity in HaCaT Cell Line Potentially Involving a GPR120/FFA4 Mediated Pathway,” Bioorganic Chemistry 108 (2021): 104657, 10.1016/J.BIOORG.2021.104657. 33556697

[123] A. T. Aborode , W. A. Awuah , T. Mikhailova , et al., “OMICs Technologies for Natural Compounds‐Based Drug Development,” Current Topics in Medicinal Chemistry 22, no. 21 (2022): 1751–1765, 10.2174/1568026622666220726092034. 35894473

[124] N. E. Thomford , D. A. Senthebane , A. Rowe , et al., “Natural Products for Drug Discovery in the 21st Century: Innovations for Novel Drug Discovery,” International Journal of Molecular Sciences 19, no. 6 (January 2018): 1578, 10.3390/ijms19061578. 29799486 PMC6032166

[125] M. A. Rahman , M. R. Rahman , T. Zaman , et al., “Emerging Potential of Naturally Occurring Autophagy Modulators Against Neurodegeneration,” Current Pharmaceutical Design 26, no. 7 (2020): 772–779, 10.2174/1381612826666200107142541. 31914904

[126] L. Fu , C. Liu , J. Zhang , and H. Yu , “Plant Natural Products as Autophagy Modulators to Improve Potential Cancer Therapy,” Studies in Natural Products Chemistry 77 (2023): 339–363, 10.1016/B978-0-323-91294-5.00010-5.

[127] M. Z. Y. Choo and C. L. L. Chai , “The Polypharmacology of Natural Products in Drug Discovery and Development,” Annual Reports in Medicinal Chemistry 61 (2023): 55–100, 10.1016/BS.ARMC.2023.10.002.

[128] I. G. Ganley , P. M. Wong , N. Gammoh , and X. Jiang , “Distinct Autophagosomal‐Lysosomal Fusion Mechanism Revealed by Thapsigargin‐Induced Autophagy Arrest,” Molecular Cell 42, no. 6 (2011): 731–743, 10.1016/J.MOLCEL.2011.04.024. 21700220 PMC3124681

[129] R. L. Wiseman , Y. Zhang , K. P. K. Lee , et al., “Flavonol Activation Defines an Unanticipated Ligand‐Binding Site in the Kinase‐RNase Domain of IRE1,” Molecular Cell 38, no. 2 (2010): 291–304, 10.1016/J.MOLCEL.2010.04.001. 20417606 PMC2864793

[130] S. W. Shin , S. Y. Kim , and J.‐W. Park , “Autophagy Inhibition Enhances Ursolic Acid‐Induced Apoptosis in PC3 Cells,” Biochimica et Biophysica Acta (BBA)—Molecular Cell Research 1823, no. 2 (2012): 451–457, 10.1016/j.bbamcr.2011.10.014. 22178132

[131] S. Deng , M. K. Shanmugam , A. P. Kumar , C. T. Yap , G. Sethi , and A. Bishayee , “Targeting Autophagy Using Natural Compounds for Cancer Prevention and Therapy,” Cancer 125, no. 8 (2019): 1228–1246, 10.1002/CNCR.31978. 30748003

[132] X. Liu , P. Zhao , X. Wang , et al., “Correction to: Celastrol Mediates Autophagy and Apoptosis via the ROS/JNK and Akt/mTOR Signaling Pathways in Glioma Cells,” Journal of Experimental & Clinical Cancer Research 38, no. 1 (2019): 284, 10.1186/s13046-019-1173-4. 31266528 PMC6604141

[133] J. Guo , X. Huang , H. Wang , and H. Yang , “Celastrol Induces Autophagy by Targeting AR/MiR‐101 in Prostate Cancer Cells,” PLoS One 10, no. 10 (2015): e0140745, 10.1371/journal.pone.0140745. 26473737 PMC4608724

[134] C. Liu , N. Li , M. Peng , et al., “Celastrol Directly Binds With VAMP7 and RAB7 to Inhibit Autophagy and Induce Apoptosis in Preadipocytes,” Frontiers in Pharmacology 14 (2023): 1094584, 10.3389/FPHAR.2023.1094584/BIBTEX. 36959859 PMC10027750

[135] J. Lu , D. Sun , S. Gao , Y. Gao , J. Ye , and P. Liu , “Cyclovirobuxine D Induces Autophagy‐Associated Cell Death via the Akt/MTOR Pathway in MCF‐7 Human Breast Cancer Cells,” Journal of Pharmacological Sciences 125, no. 1 (2014): 74–82, 10.1254/JPHS.14013FP. 24758922

[136] C. Zeng , T. Zou , J. Qu , X. Chen , S. Zhang , and Z. Lin , “Cyclovirobuxine D Induced‐Mitophagy Through the P65/BNIP3/LC3 Axis Potentiates Its Apoptosis‐Inducing Effects in Lung Cancer Cells,” International Journal of Molecular Sciences 22, no. 11 (2021): 5820, 10.3390/IJMS22115820. 34072333 PMC8199090

[137] K. Wang , J. Zhang , Y. Ge , C. Dong , and J. Dai , “Cyclovirobuxine D Inhibits Dengue Virus Replication by Impeding the Complete Autophagy in a Cholesterol‐Dependent Manner,” Science Bulletin 66, no. 3 (2021): 284–296, 10.1016/J.SCIB.2020.08.035. 36654334

[138] T. Yorimitsu , U. Nair , Z. Yang , and D. J. Klionsky , “Endoplasmic Reticulum Stress Triggers Autophagy,” Journal of Biological Chemistry 281, no. 40 (2006): 30299–30304, 10.1074/JBC.M607007200. 16901900 PMC1828866

[139] M. Ogata , S. Hino , A. Saito , et al., “Autophagy Is Activated for Cell Survival After Endoplasmic Reticulum,” Molecular and Cellular Biology 26, no. 24 (2006): 9220–9231, 10.1128/MCB.01453-06. 17030611 PMC1698520

[140] X. Wang , Z. Wei , B. Cheng , et al., “Endoplasmic Reticulum Stress Promotes HBV Production by Enhancing Use of the Autophagosome/Multivesicular Body Axis,” Hepatology 75, no. 2 (2022): 438–454, 10.1002/HEP.32178. 34580902

[141] B. Wei , F. Lu , Q. Kong , Y. Huang , K. Huang , and W. Wu , “Trehalose Induces B Cell Autophagy to Alleviate Myocardial Injury via the AMPK/ULK1 Signalling Pathway in Acute Viral Myocarditis Induced by Coxsackie Virus B3,” International Journal of Biochemistry & Cell Biology 146 (2022): 106208, 10.1016/J.BIOCEL.2022.106208. 35381374

[142] Q. Wu , D. Jiang , C. Huang , L. F. Van Dyk , L. Li , and H. W. Chu , “Trehalose‐Mediated Autophagy Impairs the Anti‐Viral Function of Human Primary Airway Epithelial Cells,” PLoS One 10, no. 4 (2015): e0124524, 10.1371/JOURNAL.PONE.0124524. 25879848 PMC4400043

[143] J.‐P. Belzile , M. Sabalza , M. Craig , E. Clark , C. S. Morello , and D. H. Spector , “Trehalose, an MTOR‐Independent Inducer of Autophagy, Inhibits Human Cytomegalovirus Infection in Multiple Cell Types,” Journal of Virology 90, no. 3 (2016): 1259–1277, 10.1128/jvi.02651-15. 26559848 PMC4719619

[144] P. Rawat , S. Hon , C. Teodorof‐Diedrich , and S. A. Spector , “Trehalose Inhibits Human Immunodeficiency Virus Type 1 Infection in Primary Human Macrophages and CD4+ T Lymphocytes Through Two Distinct Mechanisms,” Journal of Virology 94, no. 17 (2020): e00237‐20, 10.1128/JVI.00237-20. 32554696 PMC7431788

[145] C. Li , L. Huang , W. Sun , et al., “Saikosaponin D Suppresses Enterovirus A71 Infection by Inhibiting Autophagy,” Signal Transduction and Targeted Therapy 4, no. 1 (2019): 4, 10.1038/s41392-019-0037-x. 30820356 PMC6385247

[146] H. Zhu , L. Han , X. Shi , et al., “Baicalin Inhibits Autophagy Induced by Influenza A Virus H3N2,” Antiviral Research 113 (2015): 62–70, 10.1016/J.ANTIVIRAL.2014.11.003. 25446340

[147] Y. Pei , Z. P. Chen , H. Q. Ju , et al., “Autophagy Is Involved in Anti‐Viral Activity of Pentagalloylglucose (PGG) Against Herpes Simplex Virus Type 1 Infection In Vitro,” Biochemical and Biophysical Research Communications 405, no. 2 (2011): 186–191, 10.1016/j.bbrc.2011.01.006. 21216235

[148] Z. Tu , W. Gong , Y. Zhang , Y. Feng , Y. Liu , and C. Tu , “Inhibition of Rabies Virus by 1,2,3,4,6‐Penta‐O‐Galloyl‐β‐D‐Glucose Involves MTOR‐Dependent Autophagy,” Viruses 10, no. 4 (2018): 201, 10.3390/v10040201. 29673174 PMC5923495

[149] X. Wang , B. Hu , H. Hu , et al., “Tannic Acid Suppresses HBV Replication via the Regulation of NF‐ΚB, MAPKs, and Autophagy in HepG2.2.15 Cells,” Journal of Agricultural and Food Chemistry 71, no. 29 (2023): 11069–11079, 10.1021/acs.jafc.3c00863. 37450882

[150] S. E. Ha , P. Bhagwan Bhosale , H. H. Kim , et al., “Apigetrin Abrogates Lipopolysaccharide‐Induced Inflammation in L6 Skeletal Muscle Cells Through NF‐ΚB/MAPK Signaling Pathways,” Current Issues in Molecular Biology 44, no. 6 (2022): 2635–2645, 10.3390/cimb44060180. 35735621 PMC9221909

[151] R. Fang , T. Ming , J. P. L. Ng , et al., “Ciliatoside A, Isolated From Peristrophe Japonica, Inhibits HBsAg Expression and CccDNA Transcription by Inducing Autophagy,” Antiviral Research 209 (2023): 105482, 10.1016/j.antiviral.2022.105482. 36496141

[152] E. Gansukh , Z. Kazibwe , M. Pandurangan , G. Judy , and D. H. Kim , “Probing the Impact of Quercetin‐7‐O‐Glucoside on Influenza Virus Replication Influence,” Phytomedicine 23, no. 9 (2016): 958–967, 10.1016/j.phymed.2016.06.001. 27387404

[153] J. Dai , G. Wang , W. Li , et al., “High‐Throughput Screening for Anti‐Influenza A Virus Drugs and Study of the Mechanism of Procyanidin on Influenza A Virus‐Induced Autophagy,” SLAS Discovery 17, no. 5 (2012): 605–617, 10.1177/1087057111435236. 22286278

[154] X. Wang , H. Hu , B. Hu , et al., “Dihydromyricetin Inhibits Hepatitis B Virus Replication by Activating NF‐ΚB, MAPKs, and Autophagy in HepG2.2.15 Cells,” Molecular Biology Reports 50, no. 2 (2023): 1403–1414, 10.1007/s11033-022-07971-4. 36474061

[155] J. S. Min , D. E. Kim , Y. H. Jin , and S. Kwon , “Kurarinone Inhibits Hcov‐Oc43 Infection by Impairing the Virus‐Induced Autophagic Flux in Mrc‐5 Human Lung Cells,” Journal of Clinical Medicine 9, no. 7 (2020): 2230, 10.3390/jcm9072230. 32674356 PMC7408680

[156] E. Arabyan , A. Hakobyan , T. Hakobyan , et al., “Flavonoid Library Screening Reveals Kaempferol as a Potential Antiviral Agent Against African Swine Fever Virus,” Frontiers in Microbiology 12 (2021): 736780, 10.3389/fmicb.2021.736780. 34745038 PMC8567988

[157] W. Liao , X. Liu , Q. Yang , et al., “Deguelin Inhibits HCV Replication Through Suppressing Cellular Autophagy via Down Regulation of Beclin1 Expression in Human Hepatoma Cells,” Antiviral Research 174 (2020): 104704, 10.1016/J.ANTIVIRAL.2020.104704. 31917237

[158] L. Zhong , J. Hu , W. Shu , B. Gao , and S. Xiong , “Epigallocatechin‐3‐Gallate Opposes HBV‐Induced Incomplete Autophagy by Enhancing Lysosomal Acidification, Which Is Unfavorable for HBV Replication,” Cell Death & Disease 6, no. 5 (2015): e1770, 10.1038/cddis.2015.136. 25996297 PMC4669713

[159] H. Huang , D. Liao , B. He , R. Pu , Y. Cui , and G. Zhou , “Deoxyshikonin Inhibited Rotavirus Replication by Regulating Autophagy and Oxidative Stress Through SIRT1/FoxO1/Rab7 Axis,” Microbial Pathogenesis 178 (2023): 106065, 10.1016/j.micpath.2023.106065. 36907361

[160] C. C. Chang , H. L. You , H. J. Su , I. L. Hung , C. W. Kao , and S. T. Huang , “Anti‐Influenza A (H1N1) Virus Effect of Gallic Acid Through Inhibition of Virulent Protein Production and Association With Autophagy,” Food Science & Nutrition 12 (2023): 1605–1615, 10.1002/fsn3.3852. 38455214 PMC10916620

[161] S. Wang , J. Qiao , Y. Chen , L. Tian , and X. Sun , “Urolithin A Inhibits Enterovirus 71 Replication and Promotes Autophagy and Apoptosis of Infected Cells In Vitro,” Archives of Virology 167, no. 10 (2022): 1989–1997, 10.1007/s00705-022-05471-1. 35790643

[162] J. P. Dai , X. F. Zhao , J. Zeng , et al., “Drug Screening for Autophagy Inhibitors Based on the Dissociation of Beclin1‐Bcl2 Complex Using BiFC Technique and Mechanism of Eugenol on Anti‐Influenza A Virus Activity,” PLoS One 8, no. 4 (2013): e61026, 10.1371/JOURNAL.PONE.0061026. 23613775 PMC3628889

[163] S. Zhu , H. Wen , W. Wang , Y. Chen , F. Han , and W. Cai , “Anti‐Hepatitis B Virus Activity of Lithospermic Acid, a Polyphenol From Salvia Miltiorrhiza, In Vitro and In Vivo by Autophagy Regulation,” Journal of Ethnopharmacology 302 (2023): 115896, 10.1016/j.jep.2022.115896. 36334815

[164] Q. W. Wang , Y. Su , J. T. Sheng , et al., “Anti‐Influenza A Virus Activity of Rhein Through Regulating Oxidative Stress, TLR4, Akt, MAPK, and NF‐ΚB Signal Pathways,” PLoS One 13, no. 1 (2018): e0191793, 10.1371/journal.pone.0191793. 29385192 PMC5791991

[165] J. Shen , G. Wang , and J. Zuo , “Caffeic Acid Inhibits HCV Replication via Induction of IFNα Antiviral Response Through P62‐Mediated Keap1/Nrf2 Signaling Pathway,” Antiviral Research 154 (2018): 166–173, 10.1016/j.antiviral.2018.04.008. 29656059

[166] S. Fang , J. Su , B. Liang , et al., “Suppression of Autophagy by Mycophenolic Acid Contributes to Inhibition of HCV Replication in Human Hepatoma Cells,” Scientific Reports 7 (2017): 44039, 10.1038/srep44039. 28276509 PMC5343675

[167] N. R. Manchala , R. Dungdung , P. Trivedi , U. Unniyampurath , and R. Pilankatta , “Mycophenolic Acid (MPA) Modulates Host Cellular Autophagy Progression in Sub Genomic Dengue Virus‐2 Replicon Cells,” Microbial Pathogenesis 137 (2019): 103762, 10.1016/j.micpath.2019.103762. 31560972

[168] S. Song , M. Qiu , Y. Chu , et al., “Downregulation of Cellular C‐Jun N‐Terminal Protein Kinase and NF‐ΚB Activation by Berberine May Result in Inhibition of Herpes Simplex Virus Replication,” Antimicrobial Agents and Chemotherapy 58, no. 9 (2014): 5068–5078, 10.1128/AAC.02427-14. 24913175 PMC4135844

[169] H. Wang , K. Li , L. Ma , et al., “Berberine Inhibits Enterovirus 71 Replication by Downregulating the MEK/ERK Signaling Pathway and Autophagy,” Virology Journal 14, no. 1 (2017): 2, 10.1186/s12985-016-0674-4. 28081706 PMC5234143

[170] J. Wang , G. Yang , L. Zhang , et al., “Berbamine Hydrochloride Inhibits Bovine Viral Diarrhea Virus Replication via Interfering in Late‐Stage Autophagy,” Virus Research 321 (2022): 198905, 10.1016/j.virusres.2022.198905. 36064041

[171] A. P. M. Cloherty , A. G. Rader , K. S. Patel , et al., “Berbamine Suppresses Intestinal SARS‐CoV‐2 Infection via a BNIP3‐Dependent Autophagy Blockade,” Emerging Microbes & Infections 12, no. 1 (2023): 2195020, 10.1080/22221751.2023.2195020. 36951188 PMC10114999

[172] J. P. Dai , W. Z. Li , X. F. Zhao , et al., “A Drug Screening Method Based on the Autophagy Pathway and Studies of the Mechanism of Evodiamine Against Influenza A Virus,” PLoS One 7, no. 8 (2012): e42706, 10.1371/journal.pone.0042706. 22900043 PMC3416798

[173] N. C. Gassen , J. Papies , T. Bajaj , et al., “SARS‐CoV‐2‐Mediated Dysregulation of Metabolism and Autophagy Uncovers Host‐Targeting Antivirals,” Nature Communications 12, no. 1 (2021): 3818, 10.1038/s41467-021-24007-w. PMC8217552 34155207

[174] B. San‐Miguel , I. Crespo , D. Vallejo , et al., “Melatonin Modulates the Autophagic Response in Acute Liver Failure Induced by the Rabbit Hemorrhagic Disease Virus,” Journal of Pineal Research 56, no. 3 (2014): 313–321, 10.1111/jpi.12124. 24499270 PMC7166588

[175] J. H. Moon , J. M. Hong , J. W. Seol , B. Y. Park , S. K. Eo , and S. Y. Park , “Melatonin Inhibits Japanese Encephalitis Virus Replication and Neurotoxicity via Calcineurin‐Autophagy Pathways,” BMC Neuroscience 24, no. 1 (2023): 59, 10.1186/s12868-023-00832-1. 37932682 PMC10629071

[176] C. Sabino , M. Basic , D. Bender , F. Elgner , K. Himmelsbach , and E. Hildt , “Bafilomycin A1 and U18666A Efficiently Impair ZIKV Infection,” Viruses 11, no. 6 (2019): 524, 10.3390/v11060524. 31174294 PMC6630673

[177] A. De Leo , F. Colavita , F. Ciccosanti , G. M. Fimia , P. M. Lieberman , and E. Mattia , “Inhibition of Autophagy in EBV‐Positive Burkitt's Lymphoma Cells Enhances EBV Lytic Genes Expression and Replication,” Cell Death & Disease 6 (2015): e1876, 10.1038/cddis.2015.156. PMC4650432 26335716

[178] C. Zhang , B. Wei , Z. Liu , et al., “Bafilomycin A1 Inhibits SARS‐CoV‐2 Infection in a Human Lung Xenograft Mouse Model,” Virology Journal 20, no. 1 (2023): 18, 10.1186/s12985-023-01971-x. 36721152 PMC9887234

[179] L. Guo , H. Yu , W. Gu , et al., “Autophagy Negatively Regulates Transmissible Gastroenteritis Virus Replication,” Scientific Reports 6 (2016): 23864, 10.1038/srep23864. 27029407 PMC4814908

[180] Q. Liang , Z. Luo , J. Zeng , et al., “Zika Virus NS4A and NS4B Proteins Deregulate Akt‐MTOR Signaling in Human Fetal Neural Stem Cells to Inhibit Neurogenesis and Induce Autophagy,” Cell Stem Cell 19, no. 5 (2016): 663–671, 10.1016/j.stem.2016.07.019. 27524440 PMC5144538

[181] S. A. Sieber , T. Böttcher , I. Staub , and R. Orth , “Small Molecules as Versatile Tools for Activity‐Based Protein Profiling Experiments.” Comprehensive Natural Products II (Elsevier, 2010), 629–674, 10.1016/B978-008045382-8.00159-3.

[182] S. S. Petanceska and S. Gandy , “The Phosphatidylinositol 3‐Kinase Inhibitor Wortmannin Alters the Metabolism of the Alzheimer's Amyloid Precursor Protein,” Journal of Neurochemistry 73, no. 6 (1999): 2316–2320, 10.1046/J.1471-4159.1999.0732316.X. 10582589

[183] F. Teranishi , N. Takahashi , N. Gao , et al., “Phosphoinositide 3‐Kinase Inhibitor (Wortmannin) Inhibits Pancreatic Cancer Cell Motility and Migration Induced by Hyaluronan In Vitro and Peritoneal Metastasis In Vivo,” Cancer Science 100, no. 4 (2009): 770–777, 10.1111/J.1349-7006.2009.01084.X. 19469020 PMC11158346

[184] E. F. C. Blommaart , U. Krause , J. P. M. Schellens , H. Vreeling‐Sindelárová , and A. J. Meijer , “The Phosphatidylinositol 3‐Kinase Inhibitors Wortmannin and LY294002 Inhibit Autophagy in Isolated Rat Hepatocytes,” European Journal of Biochemistry 243, no. 1–2 (1997): 240–246, 10.1111/j.1432-1033.1997.0240a.x. 9030745

[185] A. Petiot , E. Ogier‐Denis , E. F. C. Blommaart , A. J. Meijer , and P. Codogno , “Distinct Classes of Phosphatidylinositol 3‐Kinases Are Involved in Signaling Pathways That Control Macroautophagy in HT‐29 Cells,” Journal of Biological Chemistry 275 (2000): 12360, 10.1016/s0021-9258(19)80895-5. 10625637

[186] N. Mizushima and T. Yoshimori , “How to Interpret LC3 Immunoblotting,” Autophagy 3, no. 6 (2007): 542–545, 10.4161/auto.4600. 17611390

[187] Z. Zhou , X. Jiang , D. Liu , et al., “Autophagy Is Involved in Influenza A Virus Replication,” Autophagy 5, no. 3 (2009): 321–328, 10.4161/auto.5.3.7406. 19066474

[188] H. Peng , B. Liu , T. Yves , et al., “Zika Virus Induces Autophagy in Human Umbilical Vein Endothelial Cells,” Viruses 10, no. 5 (2018): 259, 10.3390/v10050259. 29762492 PMC5977252

[189] A. B. Blázquez , E. Escribano‐Romero , T. Merino‐Ramos , J. C. Saiz , and M. A. Martín‐Acebes , “Infection With Usutu Virus Induces an Autophagic Response in Mammalian Cells,” PLoS Neglected Tropical Diseases 7, no. 10 (2013): e2509, 10.1371/journal.pntd.0002509. 24205422 PMC3812092

[190] J. Kindrachuk , B. Ork , B. J. Hart , et al., “Antiviral Potential of ERK/MAPK and PI3K/AKT/MTOR Signaling Modulation for Middle East Respiratory Syndrome Coronavirus Infection as Identified by Temporal Kinome Analysis,” Antimicrobial Agents and Chemotherapy 59, no. 2 (2015): 1088–1099, 10.1128/AAC.03659-14. 25487801 PMC4335870

[191] N. T. Ihle , R. Williams , S. Chow , et al., “Molecular Pharmacology and Antitumor Activity of PX‐866, a Novel Inhibitor of Phosphoinositide‐3‐Kinase Signaling,” Molecular Cancer Therapeutics 3, no. 7 (2004): 763–772. 15252137

[192] G. F. Desrochers , A. R. Sherratt , D. R. Blais , et al., “Profiling Kinase Activity During Hepatitis C Virus Replication Using a Wortmannin Probe,” ACS Infectious Diseases 1, no. 9 (2015): 443–452, 10.1021/acsinfecdis.5b00083. 27617927

[193] Q. Chen , J. Thompson , Y. Hu , A. Das , and E. J. Lesnefsky , “Metformin Attenuates ER Stress‐Induced Mitochondrial Dysfunction,” Translational Research 190 (2017): 40–50, 10.1016/J.TRSL.2017.09.003. 29040818 PMC5705457

[194] K. Kucharewicz , M. Dudkowska , A. Zawadzka , et al., “Simultaneous Induction and Blockade of Autophagy by a Single Agent,” Cell Death & Disease. 2018 93 9, no. 3 (2018): 353, 10.1038/s41419-018-0383-6. 29500364 PMC5834631

[195] L. V. Goulding , J. Yang , Z. Jiang , et al., “Thapsigargin at Non‐Cytotoxic Levels Induces a Potent Host Antiviral Response That Blocks Influenza A Virus Replication,” Viruses 12, no. 10 (2020): 1093, 10.3390/V12101093. 32992478 PMC7600819

[196] S. Al‐Beltagi , C. A. Preda , L. V. Goulding , et al., “Thapsigargin Is a Broad‐Spectrum Inhibitor of Major Human Respiratory Viruses: Coronavirus, Respiratory Syncytial Virus and Influenza A Virus,” Viruses 13, no. 2 (2021): 234, 10.3390/V13020234. 33546185 PMC7913267

[197] M. S. Shaban , C. Müller , C. Mayr‐Buro , et al., “Multi‐Level Inhibition of Coronavirus Replication by Chemical ER Stress,” Nature Communications 12, no. 1 (2021): 5536, 10.1038/s41467-021-25551-1. PMC8452654 34545074

[198] M. S. Shaban , C. Mayr‐Buro , J. Meier‐Soelch , et al., “Thapsigargin: Key to New Host‐Directed Coronavirus Antivirals?,” Trends in Pharmacological Sciences 43, no. 7 (2022): 557–568, 10.1016/J.TIPS.2022.04.004. 35534355 PMC9013669

[199] D. Piccioni , T. Juarez , B. Brown , L. Rose , V. Allgood , and S. Kesari , “ATCT‐18 Phase II Study of Mipsagargin (G‐202), a PSMA‐Activated Prodrug Targeting the Tumor Endothelium, in Adult Patients With Recurrent or Progressive Glioblastoma,” supplement, Neuro‐Oncology 17, no. Suppl 5 (2015): v5.2‐v5, 10.1093/NEUONC/NOV206.18.

[200] D. Mahalingam , J. Peguero , P. Cen , et al., “A Phase II, Multicenter, Single‐Arm Study of Mipsagargin (G‐202) as a Second‐Line Therapy Following Sorafenib for Adult Patients With Progressive Advanced Hepatocellular Carcinoma,” Cancers 11, no. 6 (2019): 833, 10.3390/CANCERS11060833. 31212948 PMC6627768

[201] J. T. Isaacs , W. N. Brennen , S. B. Christensen , and S. R. Denmeade , “Mipsagargin: The Beginning—Not the End—of Thapsigargin Prodrug‐Based Cancer Therapeutics,” Molecules 26, no. 24 (2021): 7469, 10.3390/MOLECULES26247469. 34946547 PMC8707208

[202] J. Song , G. N. He , and L. Dai , “A Comprehensive Review on Celastrol, Triptolide and Triptonide: Insights on Their Pharmacological Activity, Toxicity, Combination Therapy, New Dosage Form and Novel Drug Delivery Routes,” Biomedicine & Pharmacotherapy 162 (2023): 114705, 10.1016/J.BIOPHA.2023.114705. 37062220

[203] Y. Zhao , N. L. Hansen , Y.‐T. Duan , et al., “Biosynthesis and Biotechnological Production of the Anti‐Obesity Agent Celastrol,” Nature Chemistry 15, no. 9 (2023): 1236–1246, 10.1038/s41557-023-01245-7. 37365337

[204] H. Y. Li , J. Zhang , L. L. Sun , et al., “Celastrol Induces Apoptosis and Autophagy via the ROS/JNK Signaling Pathway in Human Osteosarcoma Cells: An In Vitro and In Vivo Study,” Cell Death & Disease 6, no. 1 (2015): e1604, 10.1038/cddis.2014.543. 25611379 PMC4669742

[205] C. Liu , N. Li , M. Peng , et al., “Celastrol Directly Binds With VAMP7 and RAB7 to Inhibit Autophagy and Induce Apoptosis in Preadipocytes,” Frontiers in Pharmacology 14 (2023): 1094584, 10.3389/fphar.2023.1094584. 36959859 PMC10027750

[206] S. Chen , Z. Li , J. Xu , et al., “Celastrol Attenuates Hepatitis C Virus Translation and Inflammatory Response in Mice by Suppressing Heat Shock Protein 90β,” Acta Pharmacologica Sinica 44, no. 8 (2023): 1637–1648, 10.1038/s41401-023-01067-w. 36882503 PMC10374583

[207] G. Gao , L. Fu , Y. Xu , et al., “Cyclovirobuxine D Ameliorates Experimental Diabetic Cardiomyopathy by Inhibiting Cardiomyocyte Pyroptosis via NLRP3 In Vivo and In Vitro,” Frontiers in Pharmacology 13 (2022): 906548, 10.3389/FPHAR.2022.906548. 35865939 PMC9294384

[208] X. Wang , H. Wu , J. An , et al., “Cyclovirobuxine D Alleviates Aldosterone‐Induced Myocardial Hypertrophy by Protecting Mitochondrial Function Depending on the Mutual Regulation of Nrf2‐SIRT3,” Biomedicine & Pharmacotherapy 167 (2023): 115618, 10.1016/J.BIOPHA.2023.115618. 37793277

[209] D. Hu , X. Liu , Y. Wang , and S. Chen , “Cyclovirobuxine D Ameliorates Acute Myocardial Ischemia by K(ATP) Channel Opening, Nitric Oxide Release and Anti‐Thrombosis,” European Journal of Pharmacology 569, no. 1–2 (2007): 103–109, 10.1016/J.EJPHAR.2007.04.038. 17555743

[210] C. Bailly and J. Zhang , “A New Horizon for the Steroidal Alkaloid Cyclovirobuxine D (Huangyangning) and Analogues: Anticancer Activities and Mechanism of Action,” Journal of Traditional Chinese Medical Sciences 7, no. 4 (2020): 337–344, 10.1016/J.JTCMS.2020.10.002.

[211] B. Yu , T. H. Fang , G. H. Lü , H. Q. Xu , and J. F. Lu , “Beneficial Effect of Cyclovirobuxine D on Heart Failure Rats Following Myocardial Infarction,” Fitoterapia 82, no. 6 (2011): 868–877, 10.1016/J.FITOTE.2011.04.016. 21575690

[212] K. Wang , J. Zhang , Y. Ge , C. Dong , and J. Dai , “Cyclovirobuxine D Inhibits Dengue Virus Replication by Impeding the Complete Autophagy in a Cholesterol‐Dependent Manner,” Science Bulletin 66, no. 3 (2021): 284–296, 10.1016/j.scib.2020.08.035. 36654334

[213] A. A. Dawood and M. A. Altobje , “Inhibition of N‐Linked Glycosylation by Tunicamycin May Contribute to the Treatment of SARS‐CoV‐2,” Microbial Pathogenesis 149 (2020): 104586, 10.1016/j.micpath.2020.104586. 33091582 PMC7573633

[214] E. M. Buckingham , J. E. Carpenter , W. Jackson , and C. Grose , “Autophagy and the Effects of Its Inhibition on Varicella‐Zoster Virus Glycoprotein Biosynthesis and Infectivity,” Journal of Virology 88, no. 2 (2014): 890–902, 10.1128/JVI.02646-13. 24198400 PMC3911683

[215] J. Zhang , L. Yao , S. Li , M. Ferdous , and P. Zhao , “ER Stress Induces Myocardial Dysfunction and Cardiac Autophagy in Sestrin2 Knockout Mice,” American Journal of Translational Research 14, no. 8 (2022): 5800–5811. 36105021 PMC9452346

[216] S. Nasrah , A. Radi , J. K. Daberkow , et al., “MAGED2 Depletion Promotes Stress‐Induced Autophagy by Impairing the CAMP/PKA Pathway,” International Journal of Molecular Sciences 24, no. 17 (2023): 13433, 10.3390/IJMS241713433/S1. 37686237 PMC10488052

[217] S. Ohtake and Y. J. Wang , “Trehalose: Current Use and Future Applications,” Journal of Pharmaceutical Sciences 100, no. 6 (2011): 2020–2053, 10.1002/JPS.22458. 21337544

[218] M. Jang , R. Park , H. Kim , et al., “AMPK Contributes to Autophagosome Maturation and Lysosomal Fusion,” Scientific Reports 8, no. 1 (2018): 12637, 10.1038/S41598-018-30977-7. 30140075 PMC6107659

[219] B. Wei , F. Lu , Q. Kong , Y. Huang , K. Huang , and W. Wu , “Trehalose Induces B Cell Autophagy to Alleviate Myocardial Injury via the AMPK/ULK1 Signalling Pathway in Acute Viral Myocarditis Induced by Coxsackie Virus B3,” International Journal of Biochemistry & Cell Biology 146 (2022): 106208, 10.1016/j.biocel.2022.106208. 35381374

[220] Q. Wu , D. Jiang , C. Huang , L. F. Van Dyk , L. Li , and H. W. Chu , “Trehalose‐Mediated Autophagy Impairs the Anti‐Viral Function of Human Primary Airway Epithelial Cells,” PLoS One 10, no. 4 (2015): e0124524, 10.1371/journal.pone.0124524. 25879848 PMC4400043

[221] P. Rawat , S. Hon , C. Teodorof‐Diedrich , and S. A. Spector , “Trehalose Inhibits Human Immunodeficiency Virus Type 1 Infection in Primary Human Macrophages and CD4 + T Lymphocytes Through Two Distinct Mechanisms,” Journal of Virology 94, no. 17 (2020): e00237‐20, 10.1128/jvi.00237-20. 32554696 PMC7431788

[222] J. Su , Y. W. Pan , S. Q. Wang , X. Z. Li , F. Huang , and S. P. Ma , “Saikosaponin‐d Attenuated Lipopolysaccharide‐Induced Depressive‐Like Behaviors via Inhibiting Microglia Activation and Neuroinflammation,” International Immunopharmacology 80 (2020): 106181, 10.1016/J.INTIMP.2019.106181. 31926446

[223] T. Gao , T. Wang , L. Wu , et al., “Saikosaponin‐d Alleviates Depression by Promoting NLRP3 Ubiquitination and Inhibiting Inflammasome Activation,” International Immunopharmacology 127 (2024): 111324, 10.1016/J.INTIMP.2023.111324. 38070467

[224] V. K. Wong , T. Li , B. Y. Law , et al., “Saikosaponin‐d, a Novel SERCA Inhibitor, Induces Autophagic Cell Death in Apoptosis‐Defective Cells,” Cell Death & Disease 4, no. 7 (2013): e720, 10.1038/CDDIS.2013.217. 23846222 PMC3730398

[225] R. Fu , L. Zhang , Y. Li , et al., “Saikosaponin D Inhibits Autophagosome‐Lysosome Fusion and Induces Autophagy‐Independent Apoptosis in MDA‐MB‐231 Breast Cancer Cells,” Molecular Medicine Reports 22, no. 2 (2020): 1026–1034, 10.3892/mmr.2020.11155. 32468000 PMC7339770

[226] G. Liu , Y. Guan , Y. Liu , et al., “Saikosaponin D Inducing Apoptosis and Autophagy Through the Activation of Endoplasmic Reticulum Stress in Glioblastoma,” BioMed Research International 2022 (2022): 5489553, 10.1155/2022/5489553. 36467888 PMC9715330

[227] J. F. Liao , W. Y. Hung , and C. F. Chen , “Anxiolytic‐Like Effects of Baicalein and Baicalin in the Vogel Conflict Test in Mice,” European Journal of Pharmacology 464, no. 2–3 (2003): 141–146, 10.1016/S0014-2999(03)01422-5. 12620506

[228] Z. Xu , F. Wang , S. Tsang , et al., “Anxiolytic‐Like Effect of Baicalin and Its Additivity With Other Anxiolytics,” Planta Medica 72, no. 02 (2006): 189–192, 10.1055/s-2005-873193. 16491459

[229] S. Singh , A. Meena , and S. Luqman , “Baicalin Mediated Regulation of Key Signaling Pathways in Cancer,” Pharmacological Research 164 (2021): 105387, 10.1016/j.phrs.2020.105387. 33352232

[230] Y. Li , K. Song , H. Zhang , et al., “Anti‐Inflammatory and Immunomodulatory Effects of Baicalin in Cerebrovascular and Neurological Disorders,” Brain Research Bulletin 164 (2020): 314–324, 10.1016/j.brainresbull.2020.08.016. 32858128

[231] Y. Wen , Y. Wang , C. Zhao , B. Zhao , and J. Wang , “The Pharmacological Efficacy of Baicalin in Inflammatory Diseases,” International Journal of Molecular Sciences 24, no. 11 (2023): 9317, 10.3390/ijms24119317. 37298268 PMC10253382

[232] K. Li , Y. Liang , A. Cheng , et al., “Antiviral Properties of Baicalin: A Concise Review,” Revista Brasileira de Farmacognosia 31, no. 4 (2021): 408–419, 10.1007/s43450-021-00182-1. 34642508 PMC8493948

[233] A. Oo , K. Rausalu , A. Merits , et al., “Deciphering the Potential of Baicalin as an Antiviral Agent for Chikungunya Virus Infection,” Antiviral Research 150 (2018): 101–111, 10.1016/j.antiviral.2017.12.012. 29269135

[234] A. Das Mahapatra , P. Bhowmik , Banerjee , et al., “Ethnomedicinal Wisdom,” in *New Look to Phytomedicine* (Elsevier, 2019), 35–61, 10.1016/B978-0-12-814619-4.00003-3.

[235] G. Liu , S. Xiong , Y. F. Xiang , et al., “Antiviral Activity and Possible Mechanisms of Action of Pentagalloylglucose (PGG) Against Influenza A Virus,” Archives of Virology 156, no. 8 (2011): 1359–1369, 10.1007/s00705-011-0989-9. 21479599

[236] P. Behrendt , P. Perin , N. Menzel , et al., “Pentagalloylglucose, a Highly Bioavailable Polyphenolic Compound Present in Cortex Moutan, Efficiently Blocks Hepatitis C Virus Entry,” Antiviral Research 147 (2017): 19–28, 10.1016/j.antiviral.2017.09.006. 28923507

[237] R. H. Chen , L. J. Yang , S. Hamdoun , et al., “1,2,3,4,6‐Pentagalloyl Glucose, a RBD‐ACE2 Binding Inhibitor to Prevent SARS‐CoV‐2 Infection,” Frontiers in Pharmacology 12 (2021): 634176, 10.3389/fphar.2021.634176. 33897423 PMC8058605

[238] H. Ge , G. Liu , Y. F. Xiang , et al., “The Mechanism of Poly‐Galloyl‐Glucoses Preventing Influenza A Virus Entry Into Host Cells,” PLoS One 9, no. 4 (2014): e94392, 10.1371/journal.pone.0094392. 24718639 PMC3981784

[239] H. S. Lim , O. S. Kim , B. Y. Kim , and S. J. Jeong , “Apigetrin From *Scutellaria baicalensis* Georgi Inhibits Neuroinflammation in BV‐2 Microglia and Exerts Neuroprotective Effect in HT22 Hippocampal Cells,” Journal of Medicinal Food 19, no. 11 (2016): 1032–1040, 10.1089/jmf.2016.0074. 27845861

[240] H. Guo , M. Li , and L. J. Xu , “Apigetrin Treatment Attenuates LPS‐Induced Acute Otitis Media Though Suppressing Inflammation and Oxidative Stress,” Biomedicine & Pharmacotherapy 109 (2019): 1978–1987, 10.1016/j.biopha.2018.07.022. 30551453

[241] Y. Zhu , S. Di , W. Hu , et al., “A New Flavonoid Glycoside (APG) Isolated From *Clematis tangutica* Attenuates Myocardial Ischemia/Reperfusion Injury via Activating PKCε Signaling,” Biochimica et Biophysica Acta (BBA)—Molecular Basis of Disease 1863, no. 3 (2017): 701–711, 10.1016/j.bbadis.2016.12.013. 28024940

[242] M. He , Z. Ren , M. U. Goraya , et al., “Anti‐Influenza Drug Screening and Inhibition of Apigetrin on Influenza A Virus Replication via TLR4 and Autophagy Pathways,” International Immunopharmacology 124 (2023): 110943, 10.1016/j.intimp.2023.110943. 37804654

[243] A. Anand David , R. Arulmoli , and S. Parasuraman , “Overviews of Biological Importance of Quercetin: A Bioactive Flavonoid,” Pharmacognosy Reviews 10, no. 20 (2016): 84, 10.4103/0973-7847.194044. 28082789 PMC5214562

[244] G. Carullo , M. Perri , F. Manetti , F. Aiello , M. C. Caroleo , and E. Cione , “Quercetin‐3‐Oleoyl Derivatives as New GPR40 Agonists: Molecular Docking Studies and Functional Evaluation,” Bioorganic & Medicinal Chemistry Letters 29, no. 14 (2019): 1761–1764, 10.1016/j.bmcl.2019.05.018. 31104992

[245] G. Carullo , P. Governa , A. Leo , et al., “Quercetin‐3‐Oleate Contributes to Skin Wound Healing Targeting FFA1/GPR40,” ChemistrySelect 4, no. 29 (2019): 8429–8433, 10.1002/slct.201902572.

[246] G. Carullo , S. Mazzotta , A. Koch , et al., “New Oleoyl Hybrids of Natural Antioxidants: Synthesis and In Vitro Evaluation as Inducers of Apoptosis in Colorectal Cancer Cells,” Antioxidants 9, no. 11 (2020): 1077, 10.3390/antiox9111077. 33153029 PMC7692320

[247] G. Carullo , A. Ahmed , A. Trezza , et al., “Design, Synthesis and Pharmacological Evaluation of Ester‐Based Quercetin Derivatives as Selective Vascular KCa1.1 Channel Stimulators,” Bioorganic Chemistry 105 (2020): 104404, 10.1016/j.bioorg.2020.104404. 33142229

[248] M. Granato , C. Rizzello , M. A. Romeo , et al., “Concomitant Reduction of C‐Myc Expression and PI3K/AKT/MTOR Signaling by Quercetin Induces a Strong Cytotoxic Effect Against Burkitt's Lymphoma,” International Journal of Biochemistry & Cell Biology 79 (2016): 393–400, 10.1016/j.biocel.2016.09.006. 27620077

[249] M. Granato , C. Rizzello , M. S. Gilardini Montani , et al., “Quercetin Induces Apoptosis and Autophagy in Primary Effusion Lymphoma Cells by Inhibiting PI3K/AKT/MTOR and STAT3 Signaling Pathways,” Journal of Nutritional Biochemistry 41 (2017): 124–136, 10.1016/j.jnutbio.2016.12.011. 28092744

[250] M. Granato , M. S. Gilardini Montani , C. Zompetta , et al., “Quercetin Interrupts the Positive Feedback Loop Between STAT3 and IL‐6, Promotes Autophagy, and Reduces ROS, Preventing EBV‐Driven B Cell Immortalization,” Biomolecules 9, no. 9 (2019): 482, 10.3390/biom9090482. 31547402 PMC6769872

[251] H. Li , Q. Li , Z. Liu , et al., “The Versatile Effects of Dihydromyricetin in Health,” Evidence‐Based Complementary and Alternative Medicine 2017 (2017): 1–10, 10.1155/2017/1053617. PMC5602609 28947908

[252] T. Xiao , Y. Wei , M. Cui , et al., “Effect of Dihydromyricetin on SARS‐CoV‐2 Viral Replication and Pulmonary Inflammation and Fibrosis,” Phytomedicine 91 (2021): 153704, 10.1016/j.phymed.2021.153704. 34419736 PMC8349562

[253] Y. Tian , H. Sang , M. Liu , et al., “Dihydromyricetin Is a New Inhibitor of Influenza Polymerase PB2 Subunit and Influenza‐Induced Inflammation,” Microbes and Infection 22, no. 6–7 (2020): 254–262, 10.1016/j.micinf.2020.05.021. 32554102

[254] W. Sun , S. Liu , A. Lu , F. Yang , and J. Duan , “In Vitro Anti‐PRV Activity of Dihydromyricetin From *Ampelopsis grossedentata* ,” Natural Product Research 36, no. 17 (2022): 4442–4445, 10.1080/14786419.2021.1982935. 34583588

[255] X. Zhao , Y. Chen , W. Zhang , et al., “Dihydromyricetin Inhibits Pseudorabies Virus Multiplication In Vitro by Regulating NF‐ΚB Signaling Pathway and Apoptosis,” Veterinary Sciences 10, no. 2 (2023): 111, 10.3390/vetsci10020111. 36851415 PMC9961748

[256] J. Li , X. Zhang , X. Shen , et al., “Phytochemistry and Biological Properties of Isoprenoid Flavonoids From *Sophora flavescens* Ait,” Fitoterapia 143 (2020): 104556, 10.1016/j.fitote.2020.104556. 32194169

[257] S. Kumar , K. S. Prajapati , M. Shuaib , P. P. Kushwaha , H. S. Tuli , and A. K. Singh , “Five‐Decade Update on Chemopreventive and Other Pharmacological Potential of Kurarinone: A Natural Flavanone.” Frontiers in Pharmacology (Frontiers Media S.A., September 27, 2021), 10.3389/fphar.2021.737137. PMC8502857 34646138

[258] D. Singh , K. Kumari , and S. Ahmed , “Natural Herbal Products for Cancer Therapy.” In *Understanding Cancer* (Elsevier, 2022), 257–268, 10.1016/B978-0-323-99883-3.00010-X.

[259] N. Sharma , S. Biswas , N. Al‐Dayan , A. S. Alhegaili , and M. Sarwat , “Antioxidant Role of Kaempferol in Prevention of Hepatocellular Carcinoma,” Antioxidants 10, no. 9 (2021): 1419, 10.3390/antiox10091419. 34573051 PMC8470426

[260] J. Wang , X. Fang , L. Ge , et al., “Antitumor, Antioxidant and Anti‐Inflammatory Activities of Kaempferol and Its Corresponding Glycosides and the Enzymatic Preparation of Kaempferol,” PLoS One 13, no. 5 (2018): e0197563, 10.1371/journal.pone.0197563. 29771951 PMC5957424

[261] H. J. Lim , R. Prajapati , S. H. Seong , H. A. Jung , and J. S. Choi , “Antioxidant and Antineuroinflammatory Mechanisms of Kaempferol‐3‐O‐β‐d‐Glucuronate on Lipopolysaccharide‐Stimulated BV2 Microglial Cells Through the Nrf2/HO‐1 Signaling Cascade and MAPK/NF‐κB Pathway,” ACS Omega 8, no. 7 (2023): 6538–6549, 10.1021/acsomega.2c06916. 36844518 PMC9948190

[262] J.‐H. Lee , D.‐H. Lee , H.‐S. Lee , J.‐S. Choi , K.‐W. Kim , and S.‐S. Hong , “Deguelin Inhibits Human Hepatocellular Carcinoma by Antiangiogenesis and Apoptosis,” Oncology Reports 20 (2008): 129–134, 10.3892/or.20.1.129. 18575727

[263] Y. Baba and Y. Kato , “Deguelin, a Novel Anti‐Tumorigenic Agent in Human Esophageal Squamous Cell Carcinoma,” EBioMedicine 26 (2017): 10, 10.1016/j.ebiom.2017.11.010. 29157837 PMC5832608

[264] Y. Baba , M. Fujii , T. Maeda , A. Suzuki , S. Yuzawa , and Y. Kato , “Deguelin Induces Apoptosis by Targeting Both EGFR‐Akt and IGF1R‐Akt Pathways in Head and Neck Squamous Cell Cancer Cell Lines,” BioMed Research International 2015 (2015): 1–9, 10.1155/2015/657179. PMC4449895 26075254

[265] X. Y. Wei , Y. F. Zeng , Q. H. Guo , et al., “Cardioprotective Effect of Epigallocatechin Gallate in Myocardial Ischemia/Reperfusion Injury and Myocardial Infarction: A Meta‐Analysis in Preclinical Animal Studies,” Scientific Reports 13, no. 1 (2023): 14050, 10.1038/s41598-023-41275-2. 37640837 PMC10462709

[266] B. H. Chen , C. H. Hsieh , S. Y. Tsai , C. Y. Wang , and C. C. Wang , “Anticancer Effects of Epigallocatechin‐3‐Gallate Nanoemulsion on Lung Cancer Cells Through the Activation of AMP‐Activated Protein Kinase Signaling Pathway,” Scientific Reports 10, no. 1 (2020): 5163, 10.1038/s41598-020-62136-2. 32198390 PMC7083948

[267] A. R. Khalatbary and H. Ahmadvand , “Anti‐Inflammatory Effect of the Epigallocatechin Gallate Following Spinal Cord Trauma in Rat,” Iranian Biomedical Journal 15, no. 1–2 (2011): 31–37. 21725497 PMC3639740

[268] N. A. Singh , A. K. A. Mandal , and Z. A. Khan , “Potential Neuroprotective Properties of Epigallocatechin‐3‐Gallate (EGCG).” Nutrition Journal (BioMed Central Ltd., June 7, 2016), 10.1186/s12937-016-0179-4. PMC4897892 27268025

[269] J. Zhou , B. L. Farah , R. A. Sinha , et al., “Epigallocatechin‐3‐Gallate (EGCG), a Green Tea Polyphenol, Stimulates Hepatic Autophagy and Lipid Clearance,” PLoS One 9, no. 1 (2014): e87161, 10.1371/journal.pone.0087161. 24489859 PMC3906112

[270] G. Honda , F. Sakakibara , K. Yazaki , and M. Tabata , “Isolation of Deoxyshikonin, an Antidermatophytic Principle From *Lithospermum erythrorhizon* Cell Cultures,” Journal of Natural Products 51, no. 1 (1988): 152–154, 10.1021/np50055a025. 3373224

[271] B. Badhani , N. Sharma , and R. Kakkar , “Gallic Acid: A Versatile Antioxidant With Promising Therapeutic and Industrial Applications,” RSC Advances 5, no. 35 (2015): 27540–27557, 10.1039/C5RA01911G.

[272] F. A. Tomás‐Barberán , A. Gil‐Izquierdo , and D. A. Moreno , “Bioavailability and Metabolism of Phenolic Compounds and Glucosinolates,” in *Designing Functional Foods* (Elsevier, 2009), 194–229, 10.1533/9781845696603.1.194.

[273] R. García‐Villalba , J. A. Giménez‐Bastida , A. Cortés‐Martín , et al., “Urolithins: A Comprehensive Update on Their Metabolism, Bioactivity, and Associated Gut Microbiota.” Molecular Nutrition and Food Research (John Wiley and Sons Inc, November 1, 2022), 10.1002/mnfr.202101019. PMC9787965 35118817

[274] F. Truzzi , A. Whittaker , E. D'amen , et al., “Wheat Germ Spermidine and Clove Eugenol in Combination Stimulate Autophagy In Vitro Showing Potential in Supporting the Immune System Against Viral Infections,” Molecules 27, no. 11 (2022): 3425, 10.3390/molecules27113425. 35684363 PMC9182079

[275] C.‐Y. Su , Q.‐L. Ming , K. Rahman , T. Han , and L.‐P. Qin , “Salvia Miltiorrhiza: Traditional Medicinal Uses, Chemistry, and Pharmacology,” Chinese Journal of Natural Medicines 13, no. 3 (2015): 163–182, 10.1016/S1875-5364(15)30002-9. 25835361

[276] Y.‐X. Zhou , W. Xia , W. Yue , C. Peng , K. Rahman , and H. Zhang , “Rhein: A Review of Pharmacological Activities,” Evidence‐Based Complementary and Alternative Medicine 2015 (2015): 1–10, 10.1155/2015/578107. PMC4491579 26185519

[277] L. Cheng , Q. Chen , R. Pi , and J. Chen , “A Research Update on the Therapeutic Potential of Rhein and Its Derivatives,” European Journal of Pharmacology 899 (2021): 173908, 10.1016/j.ejphar.2021.173908. 33515540

[278] C. Magnani , V. L. B. Isaac , M. A. Correa , and H. R. N. Salgado , “Caffeic Acid: A Review of Its Potential Use for Medications and Cosmetics,” Analytical Methods 6 (2014): 3203–3210.

[279] R. Bentley , “Mycophenolic Acid: A One Hundred Year Odyssey From Antibiotic to Immunosuppressant,” Chemical Reviews 100, no. 10 (2000): 3801–3826, 10.1021/cr990097b. 11749328

[280] M. Ramos‐Casals and J. Font , “Mycophenolate Mofetil in Patients With Hepatitis C Virus Infection,” supplement, Lupus 14, no. 3_suppl (2005): 64–72, 10.1191/0961203305LU2122OA. 15803936

[281] S. D. Henry , H. J. Metselaar , R. C. B. Lonsdale , et al., “Mycophenolic Acid Inhibits Hepatitis C Virus Replication and Acts in Synergy With Cyclosporin A and Interferon‐α,” Gastroenterology 131, no. 5 (2006): 1452–1462, 10.1053/j.gastro.2006.08.027. 17101321

[282] P. Patel , “A Bird's Eye View on a Therapeutically ‘Wonder Molecule’: Berberine,” Phytomedicine Plus (August 2021), 10.1016/j.phyplu.2021.100070.

[283] J. Liu , P. Liu , T. Xu , et al., “Berberine Induces Autophagic Cell Death in Acute Lymphoblastic Leukemia by Inactivating AKT/mTORC1 Signaling,” Drug Design, Development and Therapy 14 (2020): 1813–1823, 10.2147/DDDT.S239247. 32494123 PMC7229801

[284] H. Qu , X. Song , Z. Song , et al., “Retracted Article: Berberine Reduces Temozolomide Resistance by Inducing Autophagy via the ERK1/2 Signaling Pathway in Glioblastoma,” Cancer Cell International 20, no. 1 (2020): 592, 10.1186/s12935-020-01693-y. 33298057 PMC7727240

[285] R. Mohammadinejad , Z. Ahmadi , S. Tavakol , and M. Ashrafizadeh , “Berberine as a Potential Autophagy Modulator,” Journal of Cellular Physiology 234 (2019): 14914–14926, 10.1002/jcp.28325. 30770555

[286] C. M. Zhang , L. Gao , Y. J. Zheng , and H. T. Yang , “Berbamine Protects the Heart From Ischemia/Reperfusion Injury by Maintaining Cytosolic Ca2+ Homeostasis and Preventing Calpain Activation,” Circulation Journal 76, no. 8 (2012): 1993–2002, 10.1253/circj.CJ-11-1431. 22664727

[287] P. Parhi , S. Suklabaidya , and S. Kumar Sahoo , “Enhanced Anti‐Metastatic and Anti‐Tumorigenic Efficacy of Berbamine Loaded Lipid Nanoparticles In Vivo,” Scientific Reports 7, no. 1 (2017): 5806, 10.1038/s41598-017-05296-y. 28724926 PMC5517447

[288] Y. Ren , L. Lu , T. B. Guo , et al., “Novel Immunomodulatory Properties of Berbamine Through Selective Down‐Regulation of STAT4 and Action of IFN‐γ in Experimental Autoimmune Encephalomyelitis,” Journal of Immunology 181, no. 2 (2008): 1491–1498, 10.4049/jimmunol.181.2.1491. 18606704

[289] R. Upton , “ *Hydrastis canadensis* (Goldenseal) and Other Berberine‐Containing Botanicals,” in *Textbook of Natural Medicine* (Elsevier, 2020), 648–657.e3, 10.1016/B978-0-323-43044-9.00086-8.

[290] R. Vitello , H. Taouba , M. Derand , and J.‐F. Liégeois , “The Bis(1,2,3,4‐Tetrahydroisoquinoline) Alkaloids Cepharanthine and Berbamine Are Ligands of SK Channels,” ACS Medicinal Chemistry Letters 15, no. 2 (2024): 215–220, 10.1021/acsmedchemlett.3c00452. 38352826 PMC10860169

[291] C. Sheng , Z. Miao , and W. Zhang , “Topoisomerase I Inhibitors Derived From Natural Products: Structure–Activity Relationships and Antitumor Potency,” Studies in Natural Products Chemistry 47 (2016): 1–28, 10.1016/B978-0-444-63603-4.00001-2.

[292] N. Minois , “Molecular Basis of the ‘Anti‐Aging’ Effect of Spermidine and Other Natural Polyamines—A Mini‐Review,” Gerontology 60, no. 4 (2014): 319–326, 10.1159/000356748. 24481223

[293] M. Hayashi , H. Yamada , T. Mitamura , T. Horii , A. Yamamoto , and Y. Moriyama , “Vacuolar H+‐ATPase Localized in Plasma Membranes of Malaria Parasite Cells, *Plasmodium falciparum*, Is Involved in Regional Acidification of Parasitized Erythrocytes,” Journal of Biological Chemistry 275, no. 44 (2000): 34353–34358, 10.1074/jbc.M003323200. 10915784

[294] B. Whitton , H. Okamoto , G. Packham , and S. J. Crabb , “Vacuolar ATPase as a Potential Therapeutic Target and Mediator of Treatment Resistance in Cancer,” Cancer Medicine 7, no. 8 (2018): 3800–3811, 10.1002/cam4.1594. 29926527 PMC6089187

[295] J. J. Shacka , B. J. Klocke , and K. A. Roth , “Autophagy, Bafilomycin and Cell Death: The ‘A‐B‐Cs’ of Plecomacrolide‐Induced Neuroprotection,” Autophagy 2, no. 3 (2006): 228–230, 10.4161/auto.2703. 16874105

[296] N. Yuan , L. Song , S. Zhang , et al., “Bafilomycin A1 Targets Both Autophagy and Apoptosis Pathways in Pediatric B‐Cell Acute Lymphoblastic Leukemia,” Haematologica 100, no. 3 (2015): 345–356, 10.3324/haematol.2014.113324. 25512644 PMC4349273

[297] L. M. Ballou and R. Z. Lin , “Rapamycin and MTOR Kinase Inhibitors,” Journal of Chemical Biology 1, no. 1–4 (2008): 27–36, 10.1007/s12154-008-0003-5. 19568796 PMC2698317

[298] J. Li , S. G. Kim , and J. Blenis , “Rapamycin: One Drug, Many Effects,” Cell Metabolism 19, no. 3 (2014): 373–379, 10.1016/j.cmet.2014.01.001. 24508508 PMC3972801

[299] R. N. Saunders , M. S. Metcalfe , and M. L. Nicholson , “Rapamycin in Transplantation: A Review of the Evidence,” Kidney International 59, no. 1 (2001): 3–16, 10.1046/j.1523-1755.2001.00460.x. 11135052

[300] R. Bade , H. F. Chan , and J. Reynisson , “Characteristics of Known Drug Space. Natural Products, Their Derivatives and Synthetic Drugs,” European Journal of Medicinal Chemistry 45, no. 12 (2010): 5646–5652, 10.1016/j.ejmech.2010.09.018. 20888084

[301] S. Barth , D. Glick , and K. F. Macleod , “Autophagy: Assays and Artifacts,” Journal of Pathology 221 (June 2010): 117–124, 10.1002/path.2694. 20225337 PMC2989884

[302] I. Orhon and F. Reggiori , “Assays to Monitor Autophagy Progression in Cell Cultures,” Cells 6 (2017): 20, 10.3390/cells6030020. 28686195 PMC5617966

